# Aberrant methylation patterns in colorectal cancer: a meta-analysis

**DOI:** 10.18632/oncotarget.14590

**Published:** 2017-01-10

**Authors:** Danielle Fernandes Durso, Maria Giulia Bacalini, Ítalo Faria do Valle, Chiara Pirazzini, Massimiliano Bonafé, Gastone Castellani, Ana Maria Caetano Faria, Claudio Franceschi, Paolo Garagnani, Christine Nardini

**Affiliations:** ^1^ Department of Experimental, Diagnostic and Specialty Medicine, Alma Mater Studiorum-University of Bologna, Bologna, Italy; ^2^ National Counsel of Technological and Scientific Development (CNPq), ministry of science technology and innovation (MCTI), Brasilia, Brazil; ^3^ IRCCS Institute of Neurological Sciences, Bologna, Italy; ^4^ CAPES Foundation, Ministry of Education of Brazil–Brasília (DF), Brazil; ^5^ Department of Physics and Astronomy, University of Bologna, Bologna, Italy; ^6^ Biochemistry and Immunology Department, Biological Sciences Institute, Federal University of Minas Gerais, Belo Horizonte, Brazil; ^7^ Interdepartmental Center “L. Galvani”, University of Bologna, Bologna, Italy; ^8^ Applied Biomedical Research Center, S. Orsola-Malpighi Polyclinic, Bologna, Italy; ^9^ Personal Genomics S.r.l., Verona, Italy

**Keywords:** DNA methylation, colorectal cancer, differential analysis, network analysis, infinium human methylation 450

## Abstract

Colorectal cancer is among the leading causes of cancer death worldwide. Despite numerous molecular characterizations of the phenomenon, the exact dynamics of its onset and progression remain elusive. Colorectal cancer onset has been characterized by changes in DNA methylation profiles, that, owing to the stability of their patterns, are promising candidates to shed light on the molecular events laying at the base of this phenomenon.

To exploit this stability and reinforce it, we conducted a meta-analysis on publicly available DNA methylation datasets generated on: normal colorectal, adenoma (ADE) and adenocarcinoma (CRC) samples using the Illumina 450k array, in the systems medicine frame, searching for tumor gene episignatures, to produce a carefully selected list of potential drivers, markers and targets of the disease. The analysis proceeds from a differential meta-analysis of the methylation profiles using an analytical pipeline recently developed by our group [1], through network reconstruction, topological and functional analyses, to finally highlight relevant epigenomic features. Our results show that genes already highlighted for their genetic or transcriptional alteration in colorectal cancer are also differentially methylated, reinforcing -regardless of the level of cellular control- their role in the complex of alterations involved in tumorigenesis.

These findings were finally validated in an independent cohort from The Cancer Genome Atlas (TCGA).

## INTRODUCTION

Colorectal cancer is one of the main causes of death from cancer worldwide [2]. The development of the disease [3] is described as a progression from a pre-malignant lesion (adenomatous polyp or adenoma, ADE) arising in the normal colon epithelium, with the potential to further degenerate into a malignant lesion (colorectal adenocarcinoma, CRC) which in turn can spread to the surrounding tissues and systemically (metastasis, MET).

Evidences accumulated in the past two decades indicate that profound genetic and epigenetic changes occur in colon epithelial cells during colorectal tumorigenesis’ onset and progression [4–6]. Microsatellite and chromosomal instability have been related to the increase in genomic mutations rates that contribute to the tumor evolution [7]. Among the epigenetic modifications that are deregulated in colorectal cancer, DNA methylation has attracted great attention, thanks, also, to the rise of cost-effective genome-wide profiling methods such as the Illumina Infinium HumanMethylation27 (HM27) and HumanMethylation450 (HM450) microarrays.

In humans, DNA methylation consists of the covalent addition of a methyl group at the 5-carbon of the cytosine ring and occurs mainly in CpG dinucleotides [8]. The distribution of CpG dinucleotides and their methylation status varies widely across the genome: in the bulk of the genome CpG dinucleotides are underrepresented and tend to be pervasively methylated, while regions of high CpG density, termed CpG islands, are often found at gene promoters’ sites in a non-methylated status.

Colorectal cancer, like other solid tumors, is characterized by a profound remodeling of normal DNA methylation patterns [9, 10]. Widespread hypomethylation, up to one-third of the genes and of the bulk of the genome, has already been described three decades ago [11, 12]. More recently, Timp et al. found that large (hundreds of kb) hypomethylation blocks are a universal characteristic of colorectal cancers and other solid tumors [13]. Overall, DNA hypomethylation can contribute to tumor initiation and progression by promoting genomic instability and abnormal genes’ activation [14]. In addition, aberrant DNA hypermethylation of specific CpG islands has also been observed to occur in colorectal cancer. The CpG island methylator phenotype (CIMP) was described in a subset of colorectal cancers for the first time in 1999 [15] and was subsequently refined as hypermethylation of the five genes *CACNA1G*, *IGF2*, *NEUROG1*, *RUNX3* and *SOCS1* [16]. CIMP-positive tumors can be inspected with specific assays (Methylight) and have fundamental clinicopathological differences compared to CIMP-negative cases [15, 17–20], indicating the profound clinical impact of these alterations. More recently, methylation array platforms (HM27 and HM450) have been largely used to identify differentially methylated regions (DMRs) in adenoma and colorectal cancer [21–26], highlighting subtypes that are different in terms of clinical phenotype [27, 28] and response to treatment [10].

Despite the progresses made in characterizing the epigenomic landscape of colorectal cancer, little is still known on the pathways affected by aberrant methylation and that could be deregulated in the progression of the disease. To contribute to this research, we first selected a number of robust DMRs by aggregating 3 original studies on colorectal cancer-referred hereafter as the Naumov [25], Luo [26] and Timp [13] datasets - in a meta-analysis with the specific aim to extract robust results, secured by the independent reproducibility across different datasets. Finally, we used a fourth independent dataset, from the consortium The Cancer Genome Atlas (TCGA, [29]) to validate our previous findings.

To this aim we adopted a recently proposed analytical pipeline specifically tailored on the HM450 architecture [1], based on a multivariate approach that favors the selection of regions of adjacent CpG sites with concordant changes in DNA methylation levels [1]. The selected DMRs were further interpreted in the frame of *systems medicine* [30], using networks analysis, capable to capture the complex biological relationships that exists among the elements of a list of DMRs. From there, the identification of relevant molecules in a systemic context permits to highlight informative markers or drivers of the progression of the disease.

## RESULTS AND DISCUSSION

The starting point of our meta-analysis is the collection of three original studies on colorectal cancer, referred as the Naumov [25], Luo [26] and Timp [13] datasets briefly described below.

In Naumov et al. [25] the identification of DMRs was achieved with the comparison between normal tissue N1 and CRC (N1xCRC), using three options of the Illumina Methylation Analyzer (IMA, [31]) to identify DMRs: a site level test (15667 DMRs), a region-level gene-based test (2954 DMRs) and a region-level island-based test (3084 DMRs). Luo et al. [26] used cluster analysis to identify distinct epiphenotypes in adenomas (ADE) and adenocarcinomas (CRC). Using this approach, they identified two methylator phenotypes in ADE (Adenoma-High and Adenoma-Low) and three methylator phenotypes in CRC (Methyl-High, Methyl-Intermediate and Methyl-Low). Finally, Timp et al. used the *Bumphunter* algorithm to identify large (median length on the order of hundreds of kb) DMRs distinctive of solid tumors, including colon cancer and normal tissues, thus demonstrating that large hypomethylated blocks are a universal feature of solid tumors appearing early in tumor progression.

### Identification of DMRs between normal colorectal tissue, ADE and CRC

Table 1 shows the results of the differential analysis. The comparison N1xN2 outputted a limited number of DMRs (42, mapping on 57 genes) common to the Naumov and Luo datasets. Unsupervised hierarchical clustering using the selected DMRs showed that samples cluster primarily according to the dataset of origin (Supplementary Figure 1). We concluded that differences between tissues N1 and N2 are subtle and mainly cohort-dependent, and we did not take this comparison into further account.

**Table 1:** DMRs identified in each comparison

Selected BOPs	N1xN2	N1xADE	N1xCRC
Naumov	7868	-	10062
Luo	277	13426	5011
Timp	-	3210	6282
Shared BOPs	42	2657	2185
Shared genes	57	2180	1902
(% hypermethylated DMRs)	83	55	85

The number of DMRs identified in each dataset is reported along with the number of shared DMRs among all datasets for the same comparison and the corresponding number of genes. The percentage of hypermethylated DMRs among the shared ones is also reported.

**Figure 1:**
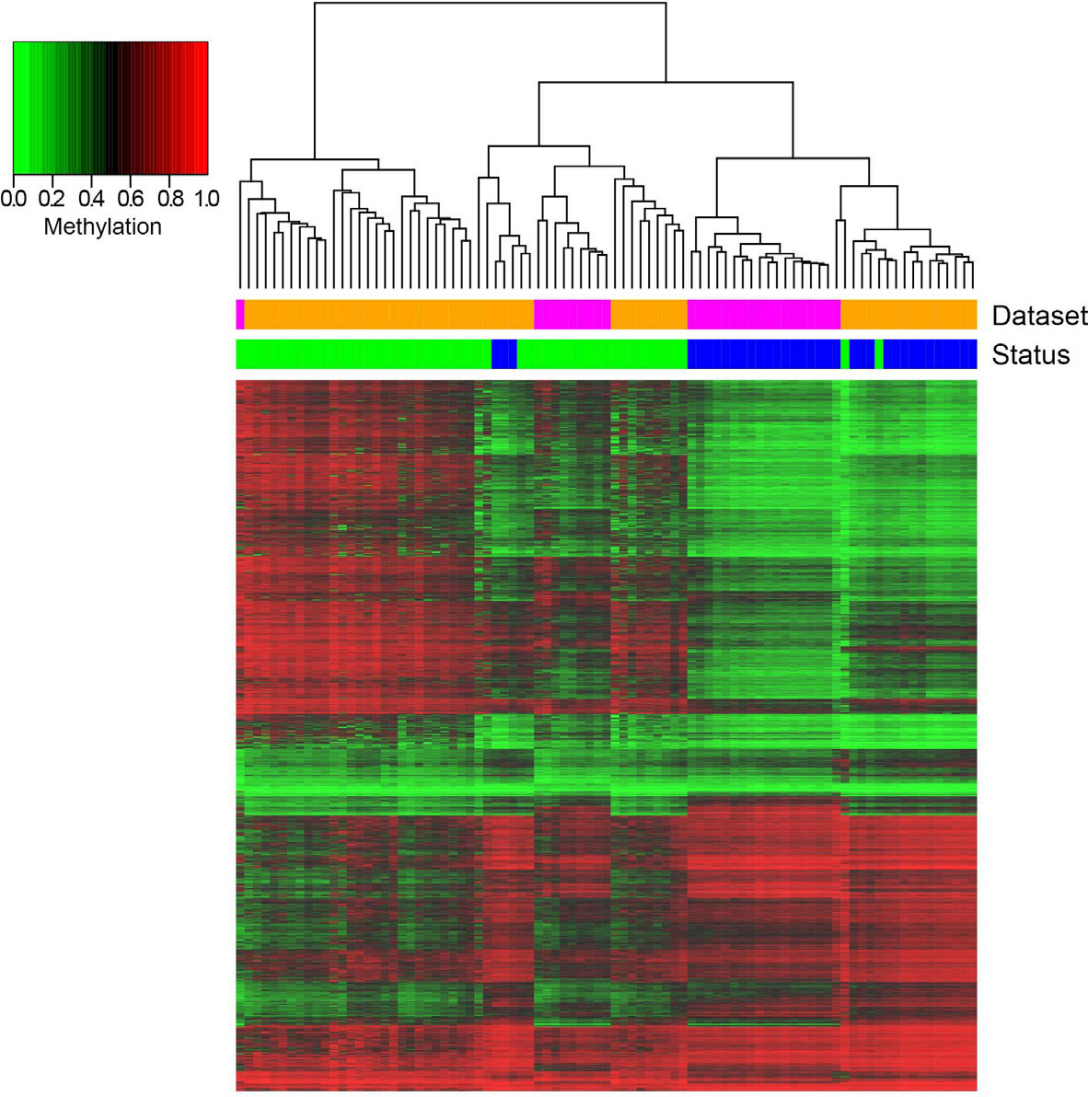
Hierarchical clustering of DMRs resulting from the comparison N1xADE and heatmap representation of their methylation values Columns correspond to samples, rows correspond to DMRs (for graphical purposes only the top significantly differential CpG of each BOP is reported). Color bars indicate the status of the samples (blue: N1; green: ADE) and the dataset of origin (orange: Luo; magenta: Timp).

In the N1xADE comparison we identified 2657 DMRs mapping on 2180 genes that were common to the Luo and Timp datasets. Unsupervised hierarchical clustering shows a clear separation of ADE versus N1 samples (Figure 1) with around half of the DMRs being hypermethylated in ADE. Notably, for these probes, ADE samples show two distinct patterns of intermediate and high hypermethylation, confirming the presence of the distinctive epiphenotypes previously described by Luo et al. [26].

**Figure 2:**
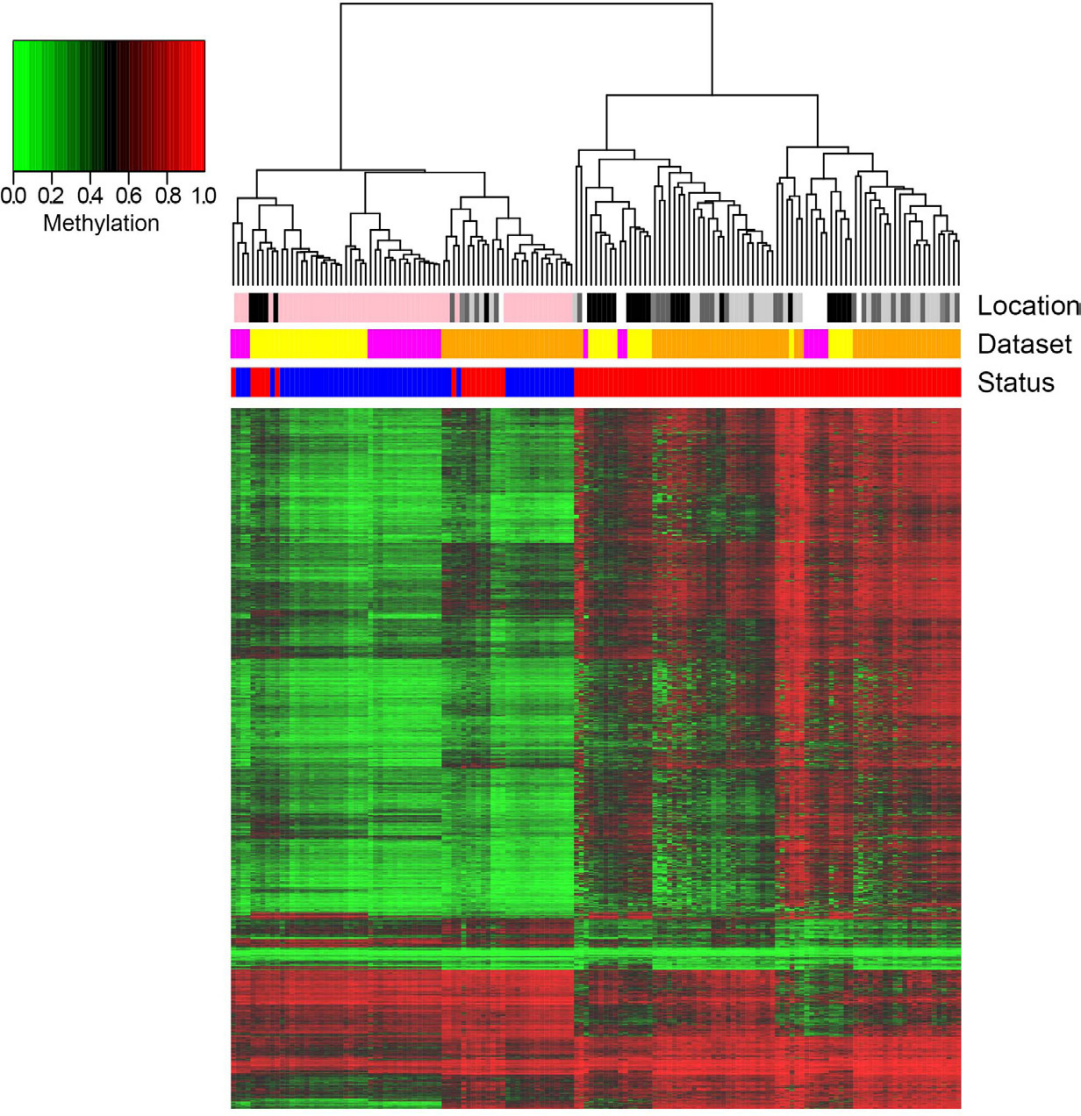
Hierarchical clustering of DMRs resulting from the comparison N1xCRC and heatmap representation of their methylation values Columns correspond to samples, rows correspond to DMRs (for graphical purposes only the top significantly differential CpG of each BOP is reported). Color bars indicate the status of the samples (blue: N1; red: CRC), the dataset of origin (yellow: Naumov; orange: Luo; magenta: Timp) and the localization of the tumor (white: unknown; light grey: distal; grey: transverse; dark grey: proximal; black: rectal; pink: normal colorectal mucosa).

N1xCRC results in 2185 DMRs mapping on 1902 genes largely overlapping with the previous findings by Naumov et al. (See Supplementay File S1). A direct comparison with the results of Timp and coworkers was not possible, as the authors’ focus was on the identification of large hypomethylated blocks and the lists of differentially methylated CpG islands were not reported. Among the identified DMRs 85% are hypermethylated in tumors. A clear separation between N1 and CRC samples was observed after unsupervised clustering analysis (Figure 2). Importantly, like in the N1xADE comparison, we confirm the epiphenotypes previously described by Luo et al., showing that CRC samples cluster in 3 groups. The first 2 groups are clearly distinct from N1 and are characterized respectively by intermediate and high levels of hypermethylation. In the third group, CRC samples have a methylation profile more similar to N1 with whom they cluster according to the dataset of origin. It is worth mentioning that, overall, samples from the same dataset tend to cluster together. This behavior can probably be ascribed to a batch effect in the generation and/or the processing of HM450 data, although we cannot exclude the presence of a biological component related for example to the method used to obtain the biological samples or to the different geographic origin of the patients.

### Inference on colorectal cancer driver mechanisms–network analysis

**Figure 3:**
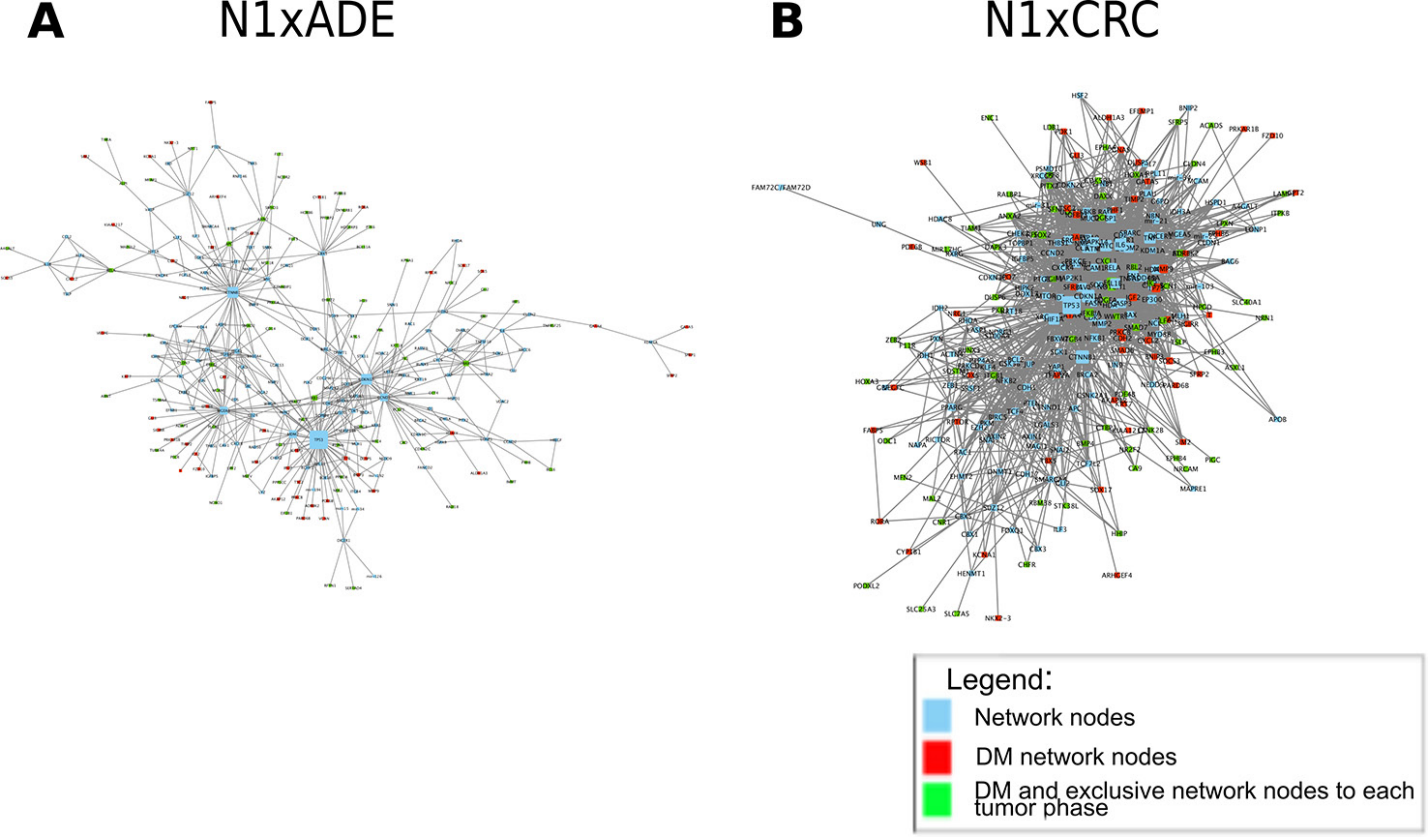
Network analysis Networks were derived with the IPA software by using differentially methylated genes in the comparisons of cancer-free patient normal tissues (N1) with ADE and CRC. Panels A and B represent networks characterized by {nodes, edges} as follows A: {257, 500}; B: {275, 1994}.

To explore further the biological mechanisms associated to the differentially methylated data and infer potential epigenetic drivers of the tumor lesion, we derived for each comparison the corresponding network (Figure 3, Supplementary File 3), and further analyzed each of those in three steps.

The first step highlights the hubs of the network, to identify biological key players [32]. Owing to the common cellular type used by the network reconstruction software we identified, as expected, a set of common nodes/genes/proteins (141), and in particular a set of hubs common to the N1xADE and N1xCRC networks (Supplementary File 5). Among them we highlight tumor suppressor p53 (*TP53*), cyclin-dependent kinase inhibitor 1A (*CDKN1A*) catenin beta-1 (*CTNNB1*) and cyclin D1 (*CCND1*), crucially involved in cell proliferation and differentiation, relevant in both tumor onset and progression.

The second analysis aims at the identification of distinctive functions characterizing each phase. For this, enrichment analysis on the nodes unique to each network (134 and 116 for N1xCRC and N1xADE, respectively) was performed (Supplementary File 5). No conclusive information could be derived from this analysis, both phases share, expectedly, a number of cancer related pathways, in addition to functions associated to early structural modification of the tissue and to the emergence of mesenchymal phenotypes (focal adhesion, WNT). This triggers the necessity of a third step of analysis, focusing on the differentially methylated hubs (DMH), distinctive of each phase.

DMHs represent 13.8 and 18.2% respectively of the total hubs in the N1xADE and N1xCRC networks, respectively (Supplementary File 4). This mildly increasing trend is in line with the numerous studies reporting a growing number of aberrant methylation features in cancers progression [33, 34]. In particular, the methylation levels in key regulator genes increases from normal to proliferative profiles, culminating with the methylation of hubs in N1xCRC involved in tumor progression, *e.g*. *NOTCH1* and *SOX2*. Following PANTHER protein class classification, we observed that most of the identified DM hub genes codify for transcription factors, signaling molecules, and cell adhesion proteins (Supplementary File 4).

In the following we describe the functions associated to some of the better known of such hubs on the basis of available literature (Table 2).

**Table 2:** The table shows the DMH in the N1xADE and N1xCRC networks ranked by increasing degree

N1xADE	N1xCRC
*SMAD2*	***IGFBP3, !***
*H2AFY*	***SOX2***
*KRT18*	***SMAD7***
***TP73***	***IGF2***
*AXIN2*	***ETS1***
***RELA***	***NOTCH1***
*RB1*	***MMP9***
*BAX*	***TP73***
	***NFKBIA***
	***EGFR***

Hypermethylated genes are displayed in bold and underlined genes are phase-specific.

### N1xADE

*AXIN2* is an important gene involved in the WNT signaling [35]. The gene Keratin 18, Type I (*KRT18*) encodes for a structural protein expressed in epithelial tissues and has been already suggested as a colorectal cancer marker [36, 37], hence confirming its importance and suggesting a mechanism of epigenetic aberration in colorectal tumors.

The hypermethylated gene V-Rel Avian Reticuloendotheliosis Viral Oncogene Homolog A (*RELA*) complexes with Nuclear Factor Of Kappa Light Polypeptide Gene Enhancer In B-Cells 1 (*NFKB1*) and forms the NFKB transcription factor. Aberrant NFKB signaling plays a role in colorectal cancer initiation and progression [38] as well as corollary processes including inflammation, immunity, differentiation, cell growth and apoptosis.

### N1xCRC

All the DM hub genes in the N1xCRC comparison are hypermethylated. They include genes that codify for transcriptional factors (*TP73* and *SOX2*) and signaling molecules (*NOTCH1*, *NFKBIA* and *EGFR*). All these genes have been known to act in deregulating important pathways driving cells to tumorigenesis, although the exact correlation between the methylation and the biological effect remains to be elucidated [33]. An important hypermethylated hub is represented by the gene that expresses the insulin-like growth factor-binding protein 3 (IGFBP3), which belongs to the IGFBPs protein class, deeply implicated in colorectal tumorigenesis [39]. Geoges et al. [40], have shown that both *IGFBP3* and *IGFBP7* areupregulated in 68 human CRC samples, an observation further refined by Hinoue et al. [41] describing a CIMP-specific epigenetic silencing of IGF-binding protein 7 (IGFBP7). Furthermore, Wajapeyee et al. [55] discussed the connection between IGFBP7 and the expression in CIMP-positive tumors of the mutated *BRAFV600E* (with *BRAF* being a DM hub in our MET network) implicated in oncogene-induced senescence in melanomas and colorectal cancers [42].

Altogether, the DM hub genes relate to common functions including apoptosis, TP53 cell signaling, hepatitis and differentiation as well as alterations of the he NFKB signaling. The NFKB family of transcription factors is pleiotropic and expressed in numerous cell types, it is known to play important roles in the immune response and is increasingly recognized as a crucial player in numerous steps of cancer initiation and progression where it cooperates with multiple other signaling molecules and pathways. Overall, pathological deregulations of the NFKB signaling are linked to inflammatory and autoimmune diseases [43] and in solid tumors NFKB acts as survival factor for transformed cells which would otherwise become apoptotic or senescent [44].

At the base of such functional alterations, genetic polymorphisms in numerous genes of the NFKB signaling pathway are a well described cause of increased risk of colorectal cancer in diverse populations around the world, including the Malaysian [45, 46] and Swedish [47], Danes [48], Swedish and Chinese populations [49, 50].

**Figure 4:**
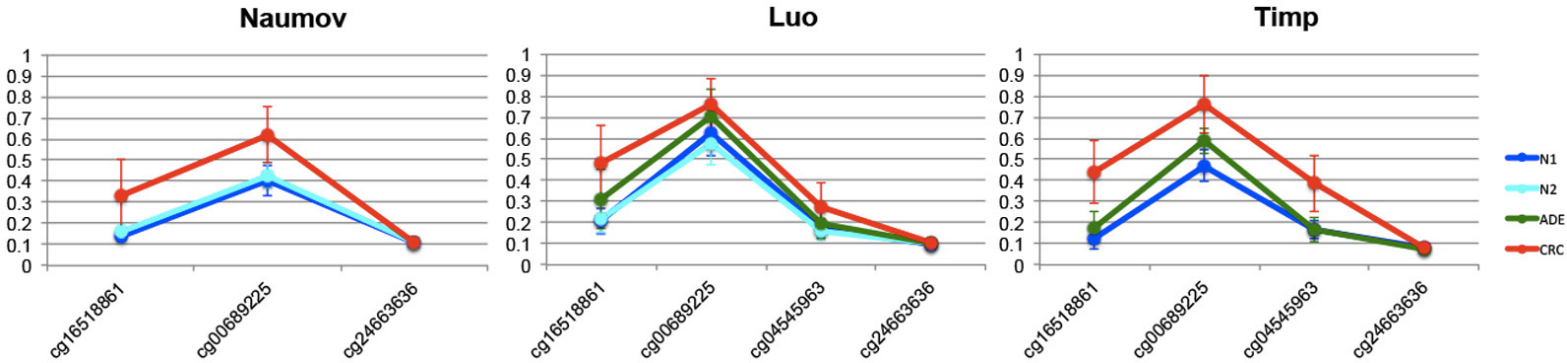
Comparison of methylation profiles of NFKBIA gene of N1xCRC The lines show mean methylation values and standard deviation for each CpG probe within the shore of chr14:35873047-35873990 island in the NFKBIA gene for the following datasets: Naumov (information on CpG cg04545963 was not available), Luo and Timp.

The obvious and yet unclear tight relation between genomic, epigenomic, transcriptional and functional alterations has already been discussed by Weisenberger et al. in the specific context of colorectal cancer [51]. Here we add a piece of evidence to this complex landscape with the observation that *NFKBIA* presents CpG sites with stable and reproducible hypermethylation profiles across all the CRC datasets (Figure 4, line plots from other DMH in Supplementary File 6).

### Independent validation: the TCGA cohort

We validated the relevance of the identified molecules (DMR and DMH) in an independent cohort from the TCGA repository including colon (COAD) and rectum tumor samples (READ).

In particular, since the TGCA dataset offered information on the age of the subjects (not available in Luo nor Naumov and only partially in Timp) we performed the analysis corrected by age, to guarantee that the results of the differential analysis were not affected by this parameter, known to impact on methylation [52].

From the differential analysis (*q*-value < 0.001) we obtained a list of 14578 DMRs, mapping to 9156 genes. Of the 2185 DMRs resulting from the N1xCRC GEO meta-analysis, 2030 were confirmed in the TCGA analysis. Further, 122/125 network DM nodes and all 10/10 DMH were validated in the TCGA results (Supplementary File 7).

As the great majority of DMRs emerging from the meta-analysis was confirmed in the TCGA cohort, we can infer that the CRC-specific DMRs that we identified are largely independent from the age of the subjects.

As an additional test we considered the DNA methylation levels of the 10 DM hub genes (corresponding to 12 BOPs) with respect to the age of the TCGA subjects and no interaction between disease status and age was evident (Supplementary File 8).

To deepen our understanding on the biological meaning and potential clinical role of the identified features (DMR and DMH), we used data mining techniques to explore how samples tend to cluster when similarity is guided by all DMR features or only by DMH.

**Figure 5:**
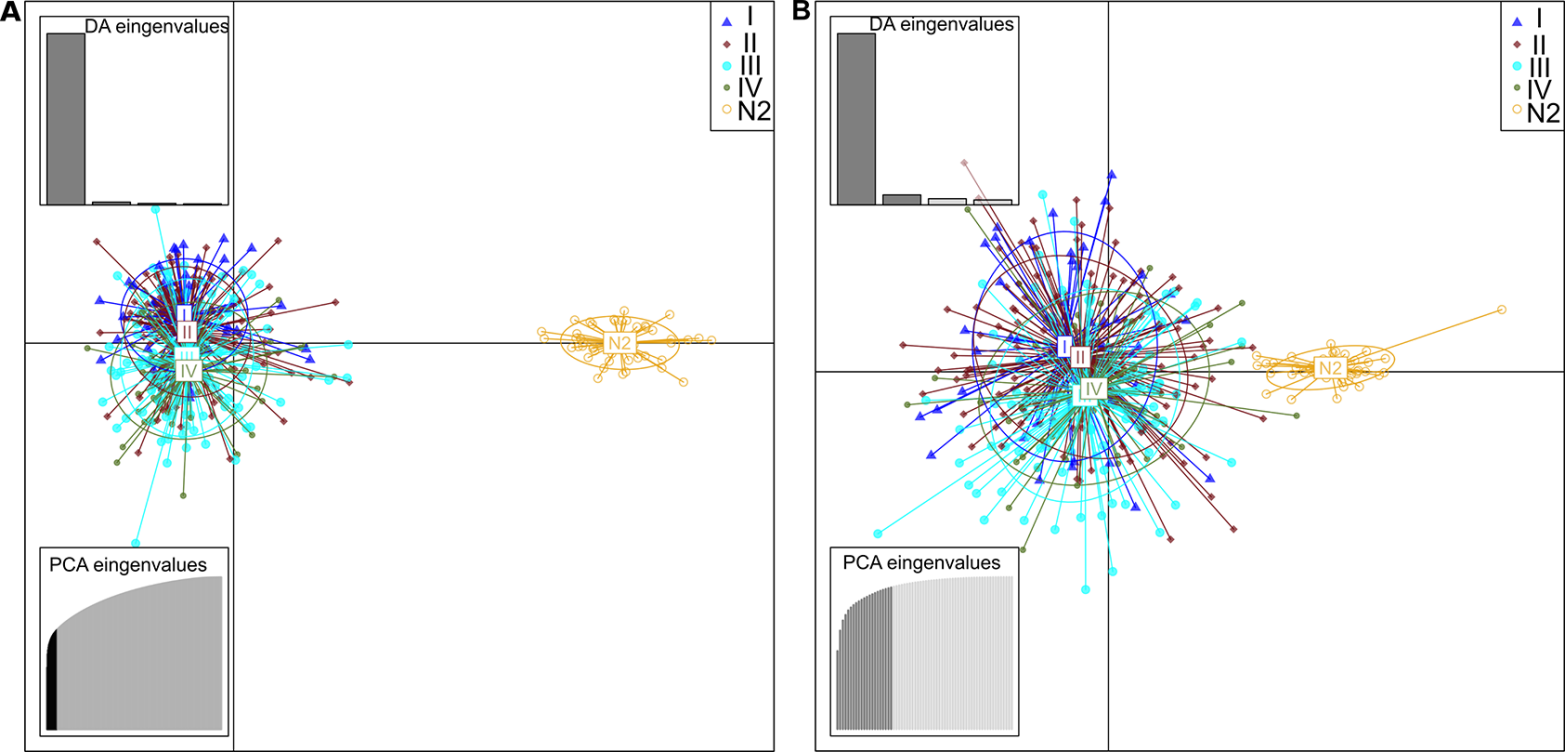
Scatterplots resulted from DAPC analysis of TCGA data These scatterplots show the first two principal components of the DAPC of data simulated according to hierarchical islands model. Clusters are shown by different colours and ellipses, while dots represent individual samples–N2 represent normal samples from affected individuals, and the groups I to IV are the corresponding I–IV stage CRC samples. The chart A is referring to DM CpG that emerged from previous analysis of N1xCRC datasets and the chart B were performed with CpGs related to the CRC networks DMH.

The DAPC analysis (Figure 5) shows that DMRs can not only separate, as expected, tumoral from normal tissue, but also permit to identify largely overalpping but still distinct clusters referring to the tumor stage.

Interestingly, this distinction is not only preserved, but mildly improved when guided only by the 10 DMH. This indicates that the additional systemic analysis run on top of the differential analysis allows to isolate a handful of features whose biological meaning is indeed able to capture the essence of the full list of DMR, and further to remove some of the noise, allowing a clearer distinction of the four stages.

Finally, owing to the larger number of clinicopathological information available in the TGCA dataset (compared to GEO) we also evaluated the DNA methylation of the 10 DM hub genes according to the mutational status of *BRAF* and *KRAS* genes (Supplementary File 9). No statistically significant differences were found when DNA methylation of these CpG sites was analyzed according to the mutational status of BRAF and KRAS genes, despite a trend towards higher methylation levels for some of the hubs (for example, SOX2 and IGF2) in patients carrying mutations in BRAF.

Overall, in this study, we observe and confirm the high reproducibility of methylation data, and hence the importance of this approach in identifying stable markers, drivers, targets of the disease. Finally, we highlight an exemplar case of methylated key molecule, emerging from the systemic approach we used, embodied by NFKBIA, a well-known genetic risk factor for colorectal cancer, here also emerging as in important epigenotype.

## MATERIAL AND METHODS

### Datasets

Infinium 450k datasets were downloaded from the Gene Expression Omnibus–GEO repository [53] using *GPL13534* (identifier of the Infinium 450k platform) and *colorectal cancer* as search terms. As to January 1^st^ 2016 we identified, based on these terms, 3 studies from colonic fresh-frozen tissues (Table 3): Naumov et al. [25] analysed 22 CRC samples, their matched healthy mucosa samples (N1) and 19 cancer-unrelated normal colon tissues (N2); Luo et al. [26] studied 41 normal colon tissues (N1), 42 colon adenomas (ADE) and 64 colorectal adenocarcinoma (CRC); Timp et al. [13] performed an epigenetic analysis of different cancer types, including colorectal cancer, and in particular measured DNA methylation in 18 normal colon tissues (N1), 10 adenoma (ADE), 9 colorectal adenocarcinoma (CRC) and 16 metastases (MET). As our focus is on meta-analysis, to guarantee robustness of the results we only included ADE and CRC samples for which more than one data-set is available. This leaves out of the picture the MET phase, for which one dataset only is available in Timp, while the heterogeneity of this phase (intense proliferation and different metastases localizations including liver and lung) strongly demands the natural filtering of the meta-analysis.

**Table 3:** GEO datasets used in the meta-analysis

	Naumov	Luo	Timp
GEO ID	GSE42752	GSE48684	GSE53051
**N1**	20	17	18
**N2**	21	24	–
**ADE**	–	42	10
**CRC**	22	64	9
**Total samples**	63	147	53

N1: Normal colorectal tissue from cancer-free subjects; N2: Normal colorectal tissue from subjects with colorectal cancer; ADE: Colorectal samples from adenoma lesions; CRC: Colorectal samples from CRC patients.

In order to validate our findings we used an independent Infinium 450k dataset from The Cancer Genome Atlas (TCGA) project [29], including data from colorectal adenocarcinoma (COAD) and rectum adenocarcinoma (READ). Methylation data, demographic and clinical information of 418 samples were downloaded from the TCGA data portal (http://firebrowse.org, [54]): they included 323 colon (285 tumor samples, 38 of which had also a normal counterpart) and 95 rectum samples (88 tumor samples, 7 of which had also a normal counterpart) [55].

### Differential analysis

DMRs were defined and selected according to the multivariate approach proposed by Bacalini et al. [1] to compare the methylation of blocks of 3 or more adjacent CpG probes (Block Of Probes, BOPs) between groups. In the present meta-analysis, the focus is on BOPs localized in CpG islands and CpG islands-surrounding sequences (shores and shelves) that map on genic regions. The rationale underlying this approach is that, compared to punctual changes in DNA methylation of a single CpGs, concomitant alterations in adjacent CpGs are more likely to modify the chromatin structure and hence gene expression and, more in general, to affect biological functions [1]. Correction for multiple hypotheses comparison was done by Benjamini-Hochberg (False discovery rate–FDR, *q*-value < 0.001, [56]).

To guarantee robustness while avoiding loss of information we requested that the retained candidates show unanimous statistical significance across all datasets, without further limitations on the absolute value of the differential methylation (delta), owing to the recent findings on the relevance of minor changes in DNA methylation levels in terms of phenotypical consequences [57]. Delta distributions are nevertheless provided in Supplementary Figures 2 and 3 showing that less than 2% of the samples have delta below 0.05.

Three pairwise comparisons (N1xN2, N1xADE and N1xCRC) were run. In the TCGA validation cohort, the 45 N2 samples were compared with 373 CRC samples. In this last comparison, associations were corrected for the age of the subjects, an information that was not available for the 3 GEO datasets. In the following, if not explicitly stated, DMRs always refer to significantly differentially methylated BOPs.

### Network analysis

For each comparison, we identified robust results by selecting across all 3 datasets only common and concordant (coherently hyper- or hypo-methylated) DMRs (Supplementary File 1).

Network analysis was adopted to gain understanding on the biological interconnections occurring among the DMRs identified in each meta-list: DMRs genes were set as nodes of the network, and connecting biochemical relations were reconstructed importing the gene lists into Ingenuity Pathway Analysis (IPA, Qiagen v.1-04 [58], (Supplementary File 2). To improve the reconstruction, IPA adds external (non-differentially methylated) items including genes and genes products, mRNA, miRNA, proteins, from here on interchangeably named nodes. These additional nodes are extracted from IPA internal data-base and filtered by the *Tissues & Cell Lines* parameter here set to *large intestine* and *colon cancer cells*. To guarantee proper reconstruction, Qiagen recommends to output from each meaningful list of molecules a maximum of 10 networks with a maximum of 70 nodes each. Successively, all networks sharing nodes (interconnected) were merged in one large interconnected map, leading to one network per comparison.

From there, we first explored the networks’ nodes based on their topological characteristics, known to match biological relevance [59]. In particular, topological analyses were performed on nodes degree, i.e. the number of connections stemming from a node/gene [60]. Degree is an intuitive measure to determine the importance of a node, indicating that the more the node is connected, the more genes/proteins/molecules it interacts with and the more pathways it is involved in. Nodes with degree above a certain threshold (here 80th percentile [61]) are defined as *hubs*. For such topological analyses, the network generated by IPA was input into Cytoscape v 3.3.0 [62], a network analyzer software (Supplementary File 3).

We further explored network genes’ meaning with functional enrichment analysis for KEGG pathways with WebGestalt online tool [63] on a selection of nodes belonging exclusively to each tumor stage i.e. by excluding the nodes shared by all networks (Supplementary File 2). This selected list of genes was manually curated first with PANTHER [64] to identify the protein class and the general functional importance of the hubs (See Supplementary File 4) and then from literature to highlight key regulators of the disease and discuss potential mechanisms driver of tumor onset and progression.

Finally, a list of the topologically relevant DMRs (DMH, i.e. differentially methylated hubs) in each network was filtered to select the genes exclusive to each tumor stage, leading to the final discussion on genes of topological and functional relevance, characterized by an aberrant methylation state in the disease.

### Statistical analyses

Statistical analyses were performed using the computing environment R. Discriminant Analysis of Principal Componentes (DAPC) was performed using the R package *adegenet* [65].

## SUPPLEMENTARY MATERIALS




















