# Supplementary material for: Aberrant methylation patterns in colorectal cancer: a meta-analysis
Source: Oncotarget. 2017 Jan 10;8(8):12820–30. doi: 10.18632/oncotarget.14590 (PMC5355058; doi:10.18632/oncotarget.14590)
Supplement: Supplementary file 1 [file oncotarget-08-12820-s001.pdf]

Aberrant methylation patterns in colorectal cancer: a meta-analysis

Supplementary Materials

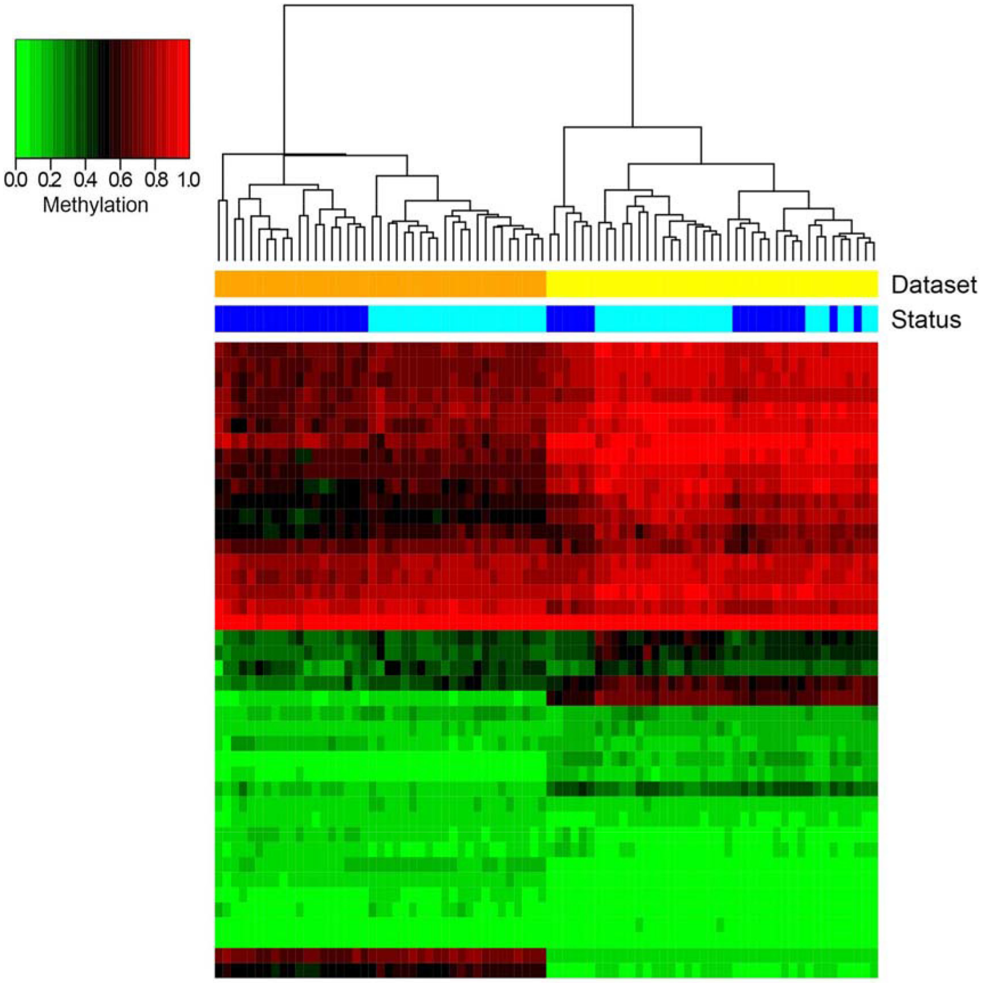

Supplementary Figure 1: Hierarchical clustering of DMRs resulting from the comparison N1xN2 and heatmap representation of their methylation values. Columns correspond to samples, rows correspond to DMRs (for graphical purposes only the top significantly differential CpG of each BOP is reported). Color bars indicate the status of the samples (blue: N1; cyan: N2) and the dataset of origin (orange: Naumov; yellow: Luo).

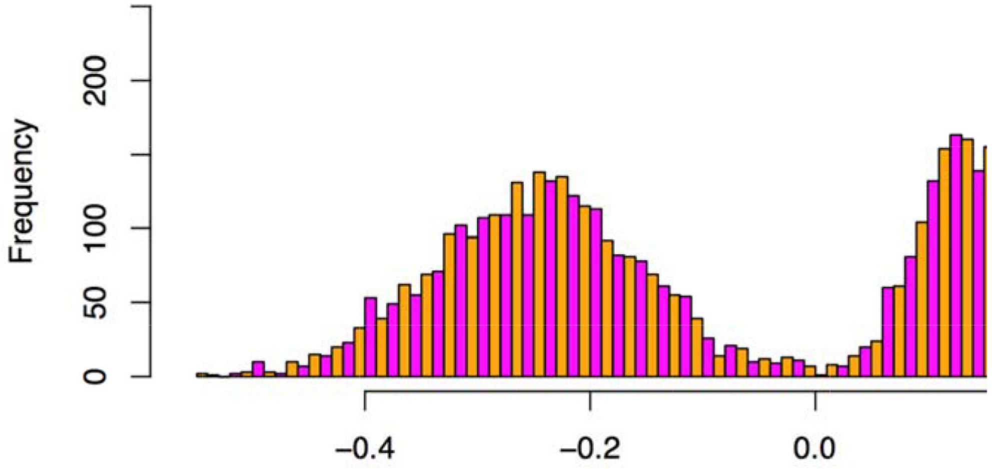

Supplementary Figure 2: Histogram of N1xADE analysis–Frequency of delta methylation values to Luo and Timp N1xADE analysis considering all significant DM BOPs using an *q*-value of 0.001.

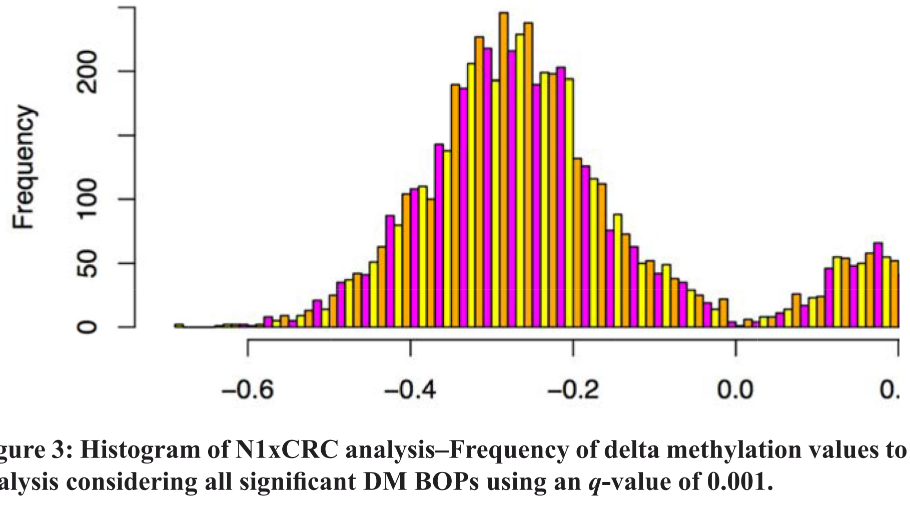

Supplementary Figure 3: Histogram of N1xCRC analysis–Frequency of delta methylation values to Naumov, Luo and Timp N1xCRC analysis considering all significant DM BOPs using an *q*-value of 0.001.

Supplementary File 1: Differentially methylated BOPs for each colorectal cancer phase studied. See Supplementary_File_2

Supplementary File 2: Network genes and DM genes exclusive to each colorectal cancer phase studied. See Supplementary_File_2

Supplementary File 3: Network nodes, topological parameters and funcional informations. See Supplementary_File_3

Supplementary File 4: Network hubs to each colorectal cancer phase studied. See Supplementary_File_4

Supplementary File 5: Kegg pathways analysis of the network genes (nodes). See Supplementary_File_5

Supplementary File 6: Comparison of methylation profiles of genes DM hubs of N1xADE and N1xCRC. See Supplementary_File_6

Supplementary File 7: The Cancer Genome Atlas COADREAD samples characteristics. See Supplementary_File_7

Supplementary File 8: Scatter plots Age versus CpGs methylation levels; plus linear regression from TCGA dataset. See Supplementary_File_8

Supplementary File 9: Boxplots showing methylation levels by BRAF and KRAS gene mutation status–abnormal and normal. See Supplementary_File_9
