# Supplementary material for: Aberrant methylation patterns in colorectal cancer: a meta-analysis
Source: Oncotarget. 2017 Jan 10;8(8):12820–30. doi: 10.18632/oncotarget.14590 (PMC5355058; doi:10.18632/oncotarget.14590)

**Supplementary File 6** - Comparison of methylation profiles of genes DM hubs of N1xADE and N1xCRC. Curves showing the DNA methylation levels of significant block of probes (BOPs) mapped into genes that resulted as hubs (Table 3). The lines show mean methylation values and standard deviation for each CpG probe within the selected BOP for each dataset:

**PAGES 2 to 12**

N1xADE – Luo dataset: The line colors A, B C and D represent respectively N1, N2, ADE and CRC cancer phases.

**PAGES 13 to 23**

N1xADE – Timp dataset: The line colors A, B C and D represent respectively N1, ADE, CRC and MET cancer phases.

**PAGES 24 to 36**

N1xCRC – Naumov dataset: The line colors A, B and C represent respectively N1, N2 and CRC and met cancer phases.

**PAGES 37 to 49**

N1xCRC – Luo dataset: The line colors A, B C and D represent respectively N1, N2, ADE and CRC cancer phases.

**PAGES 50 to 62**

N1xCRC – Timp dataset: The line colors A, B C and D represent respectively N1, ADE, CRC and MET cancer phases.


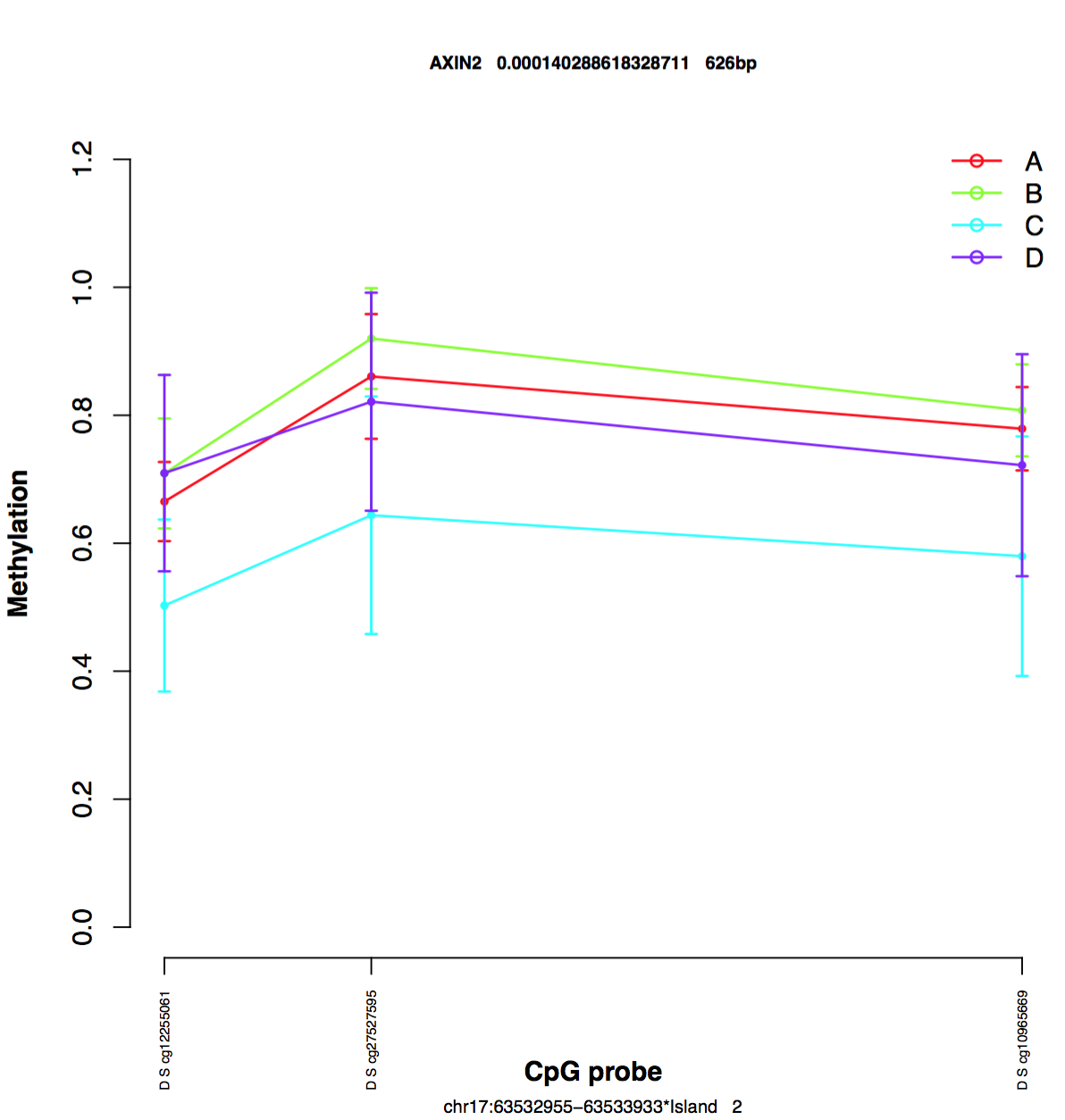

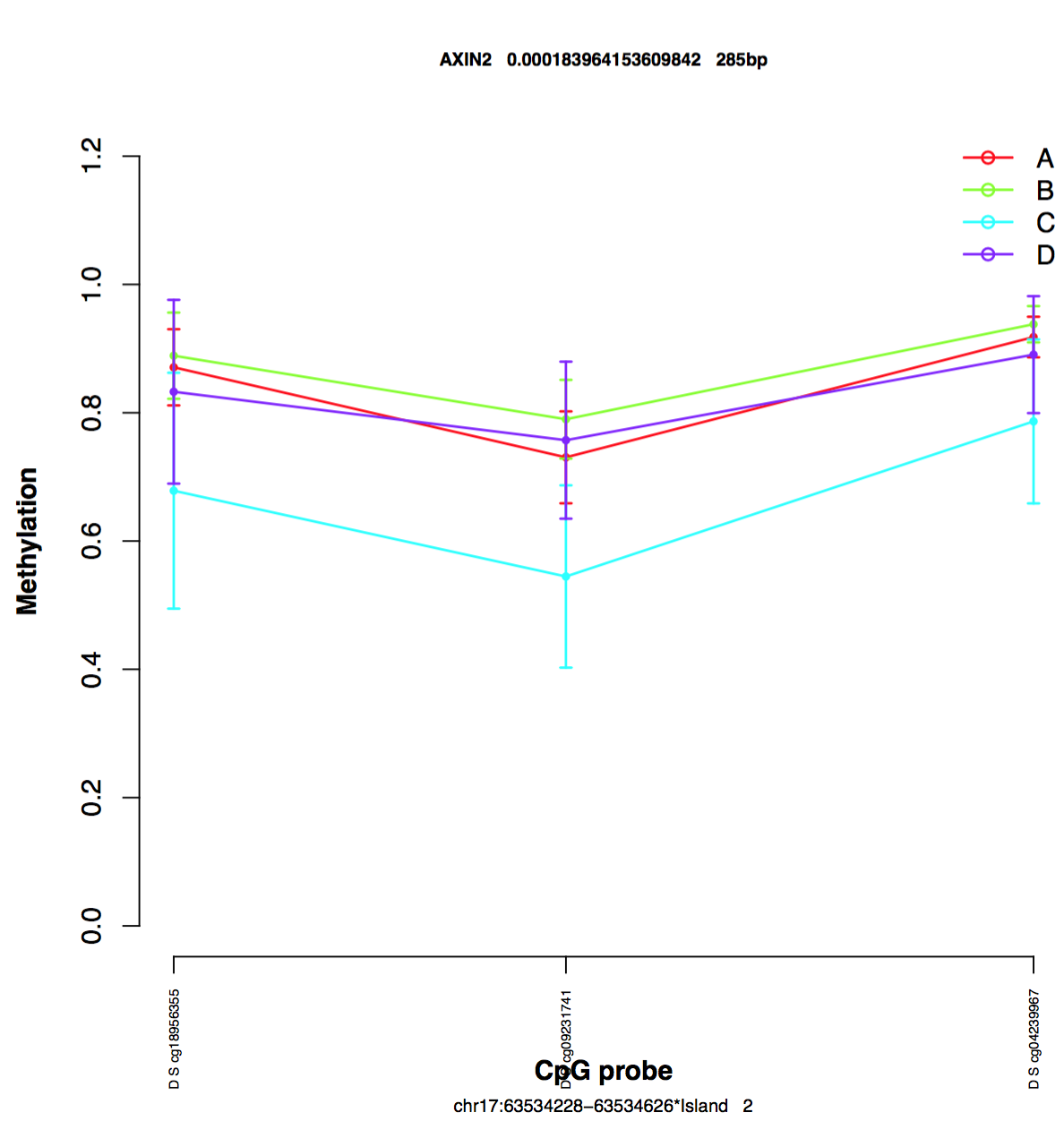

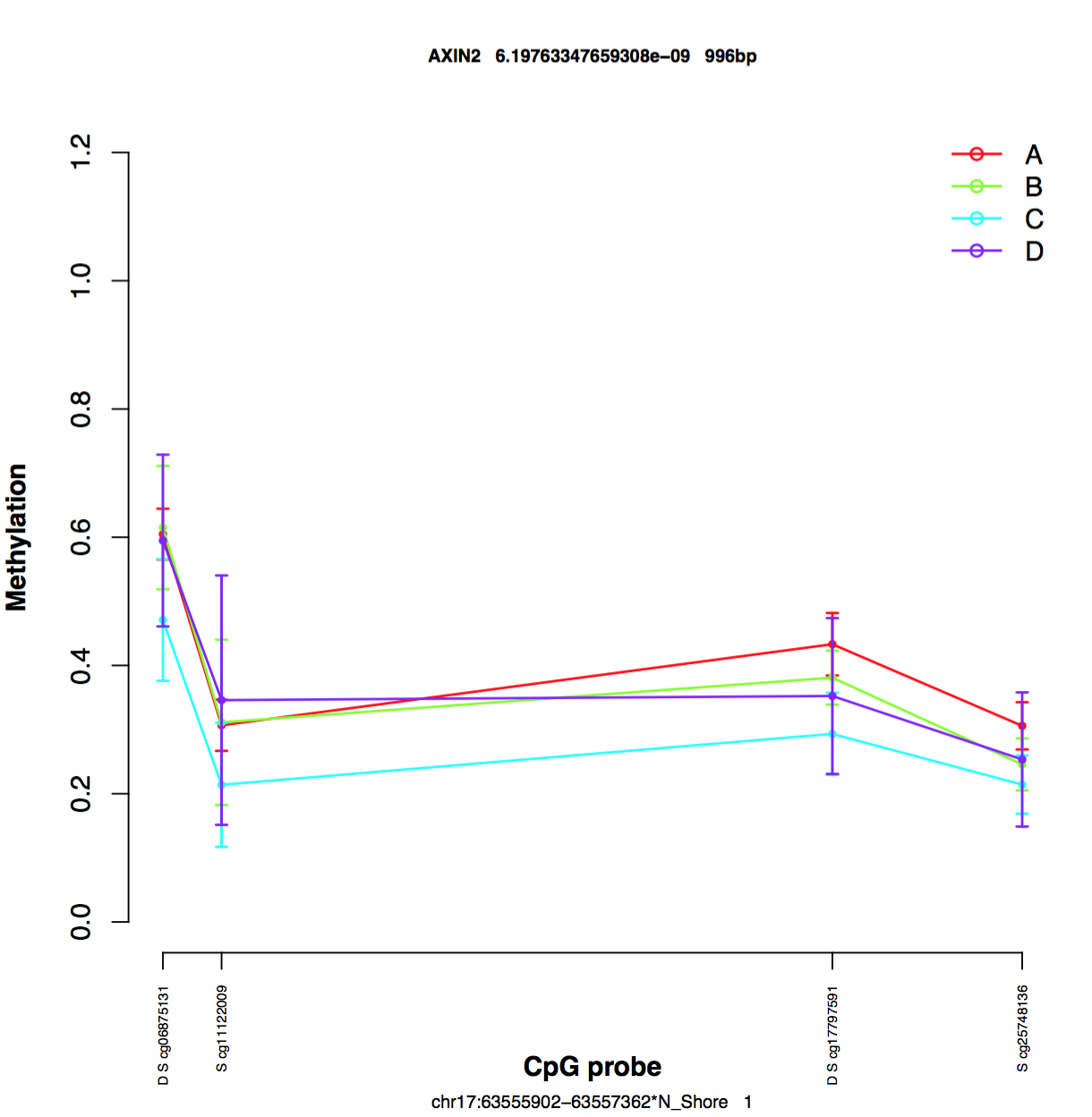

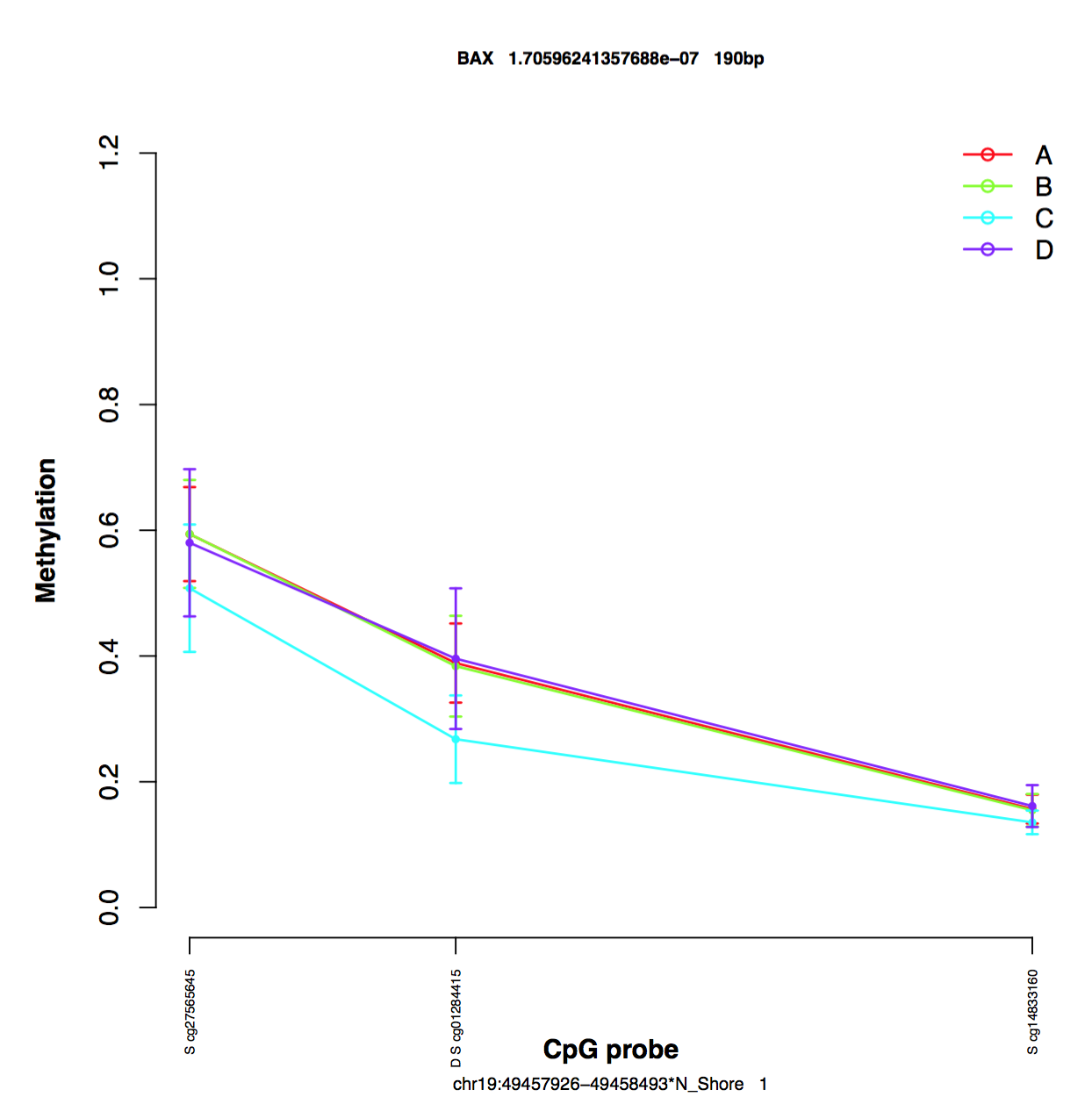

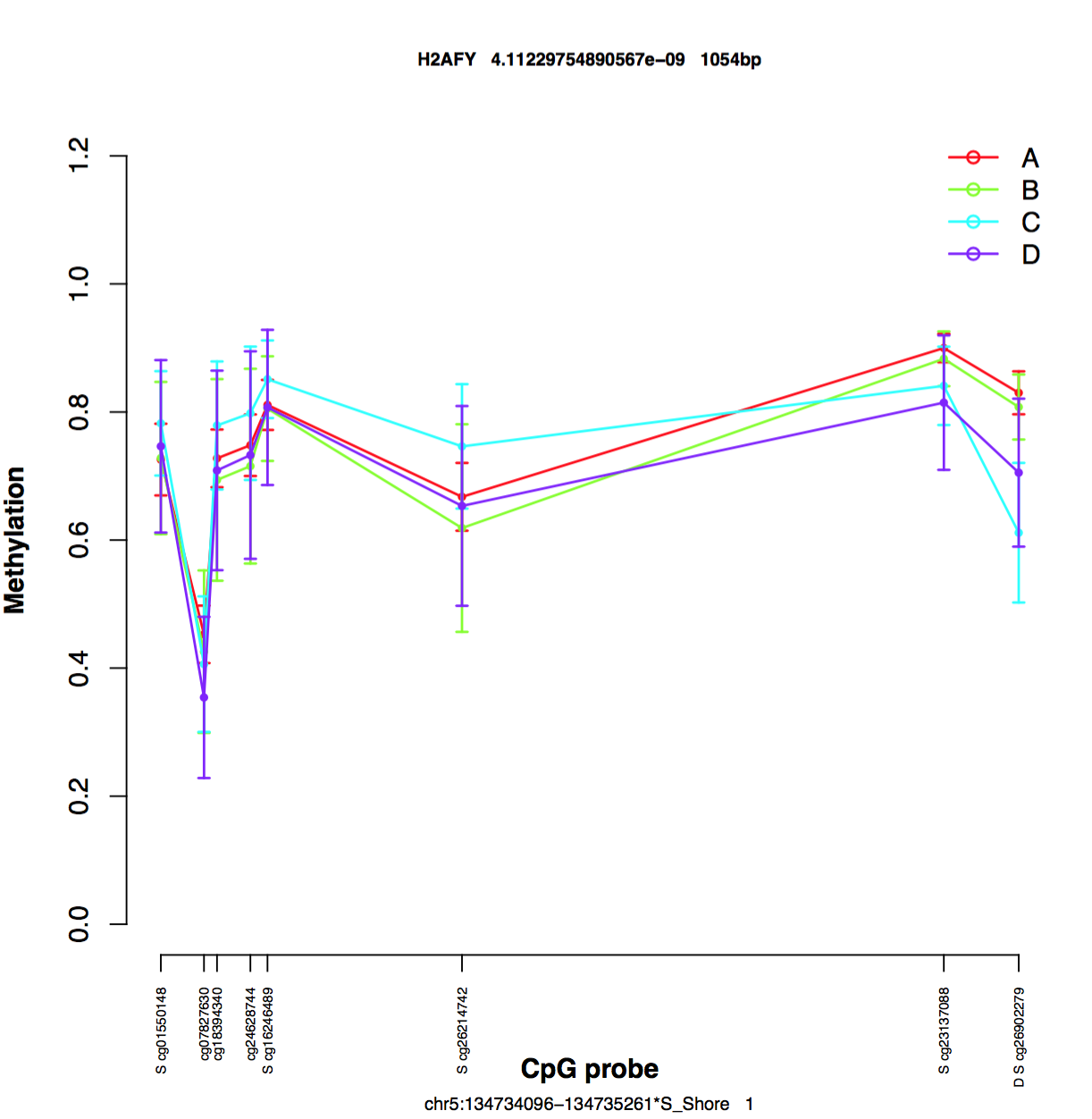

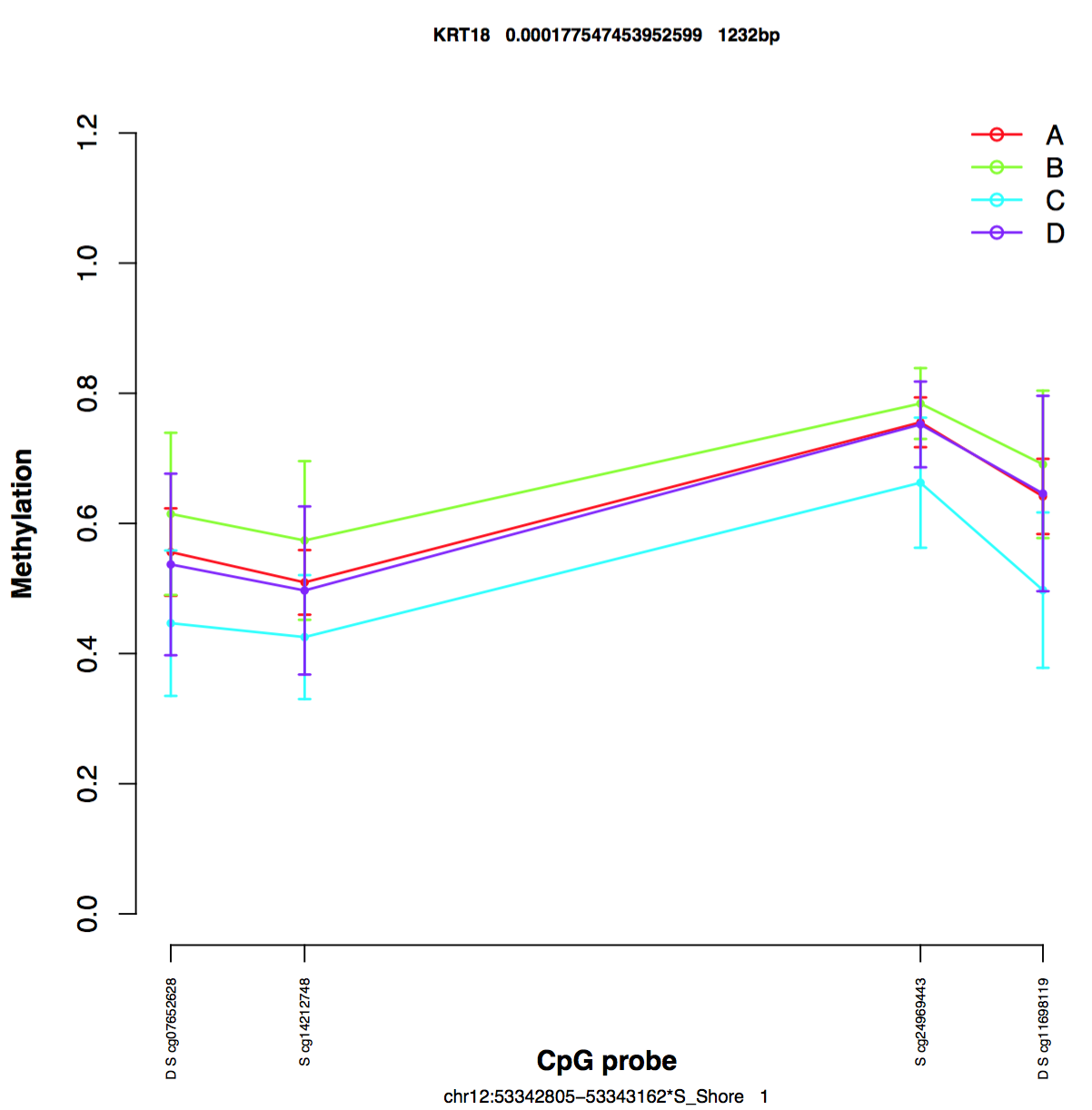

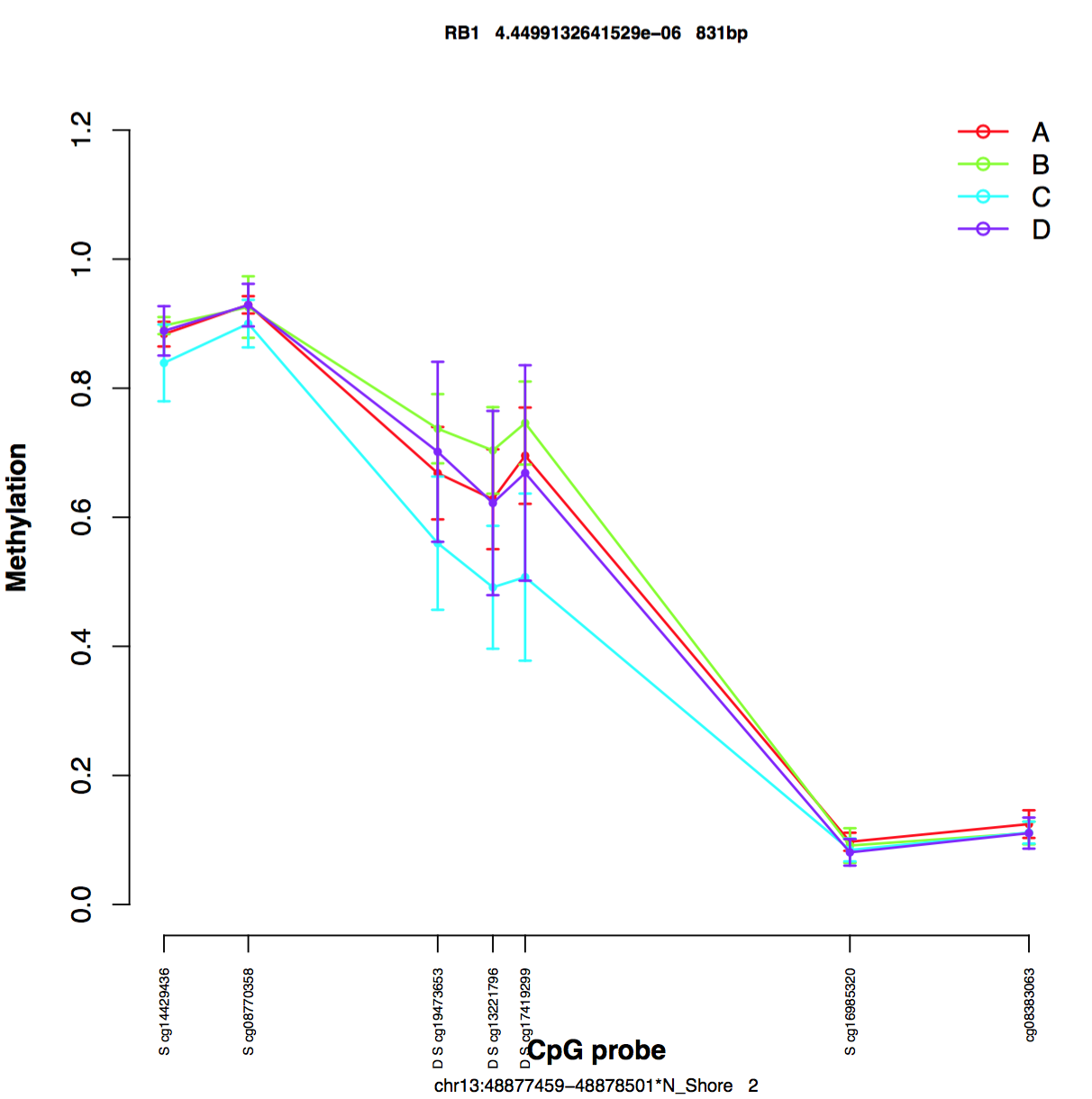

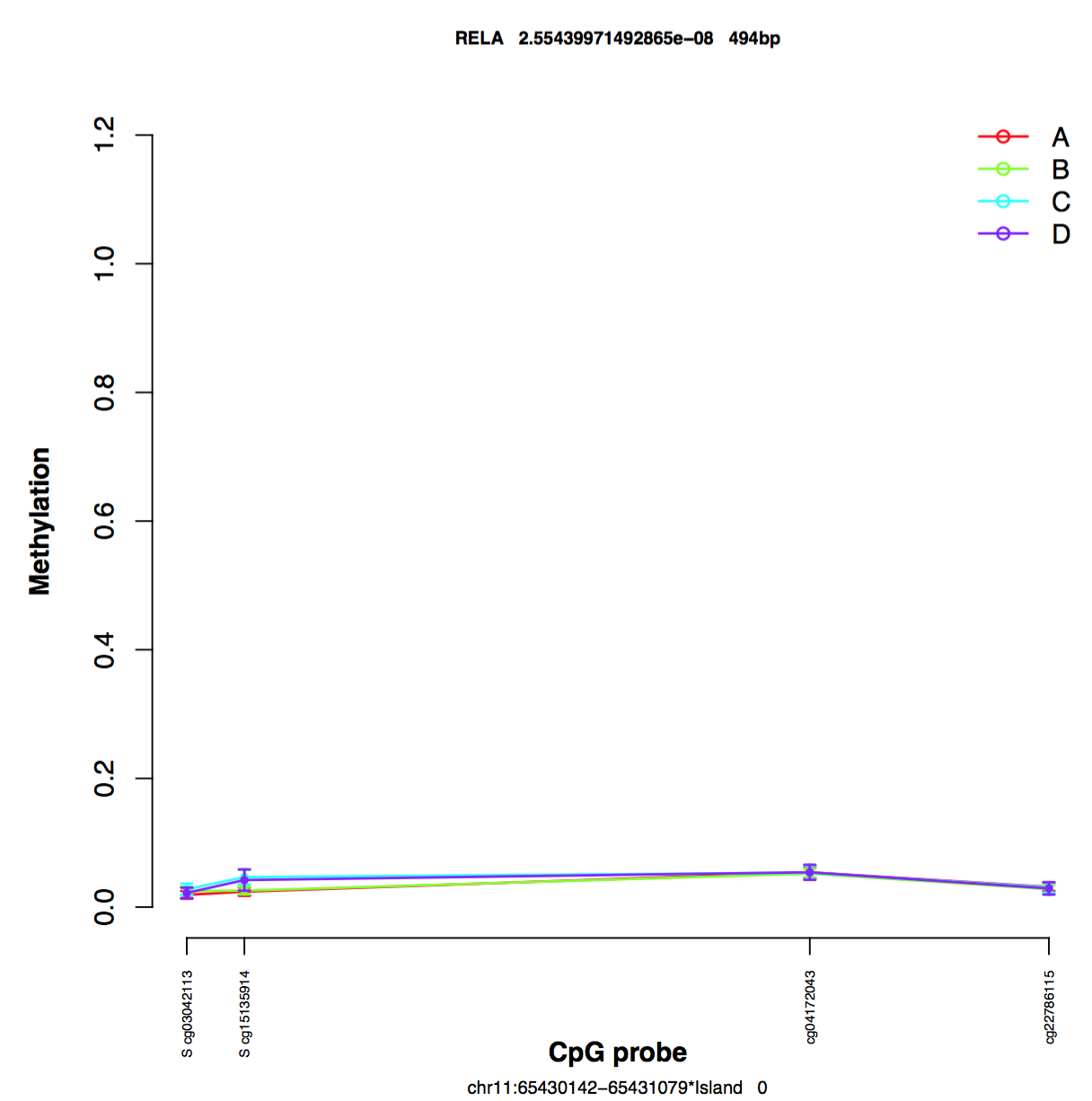

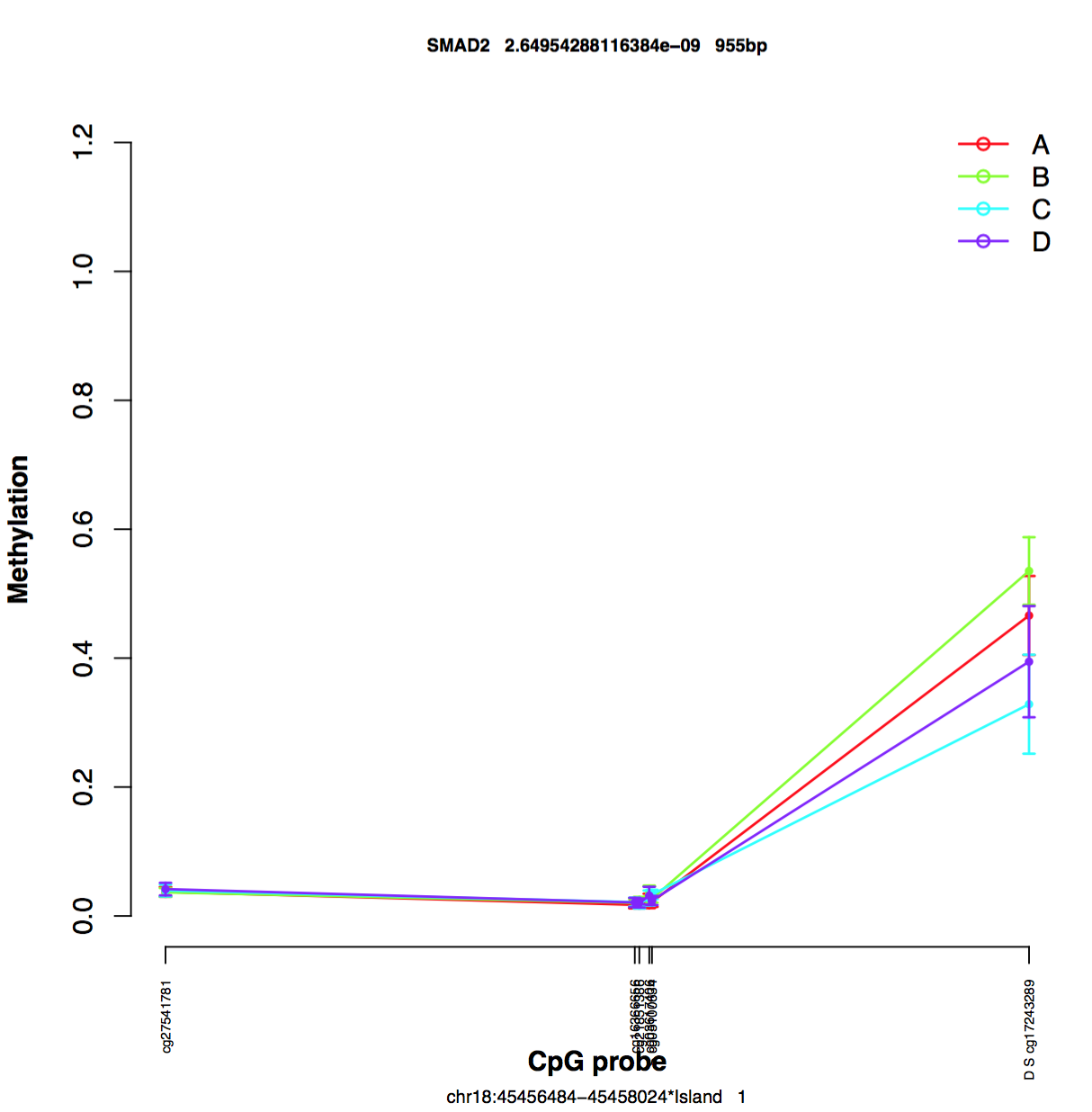

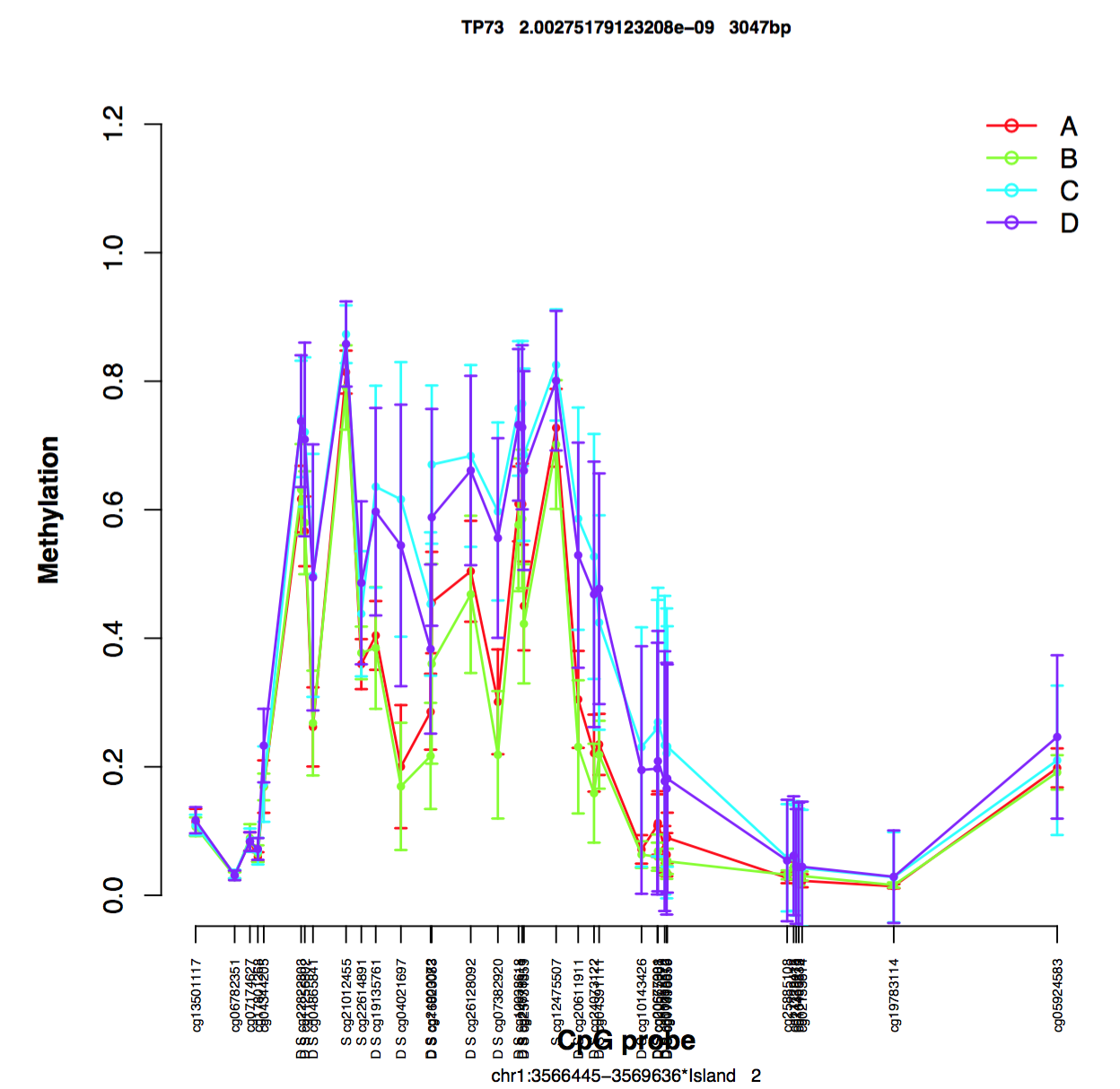

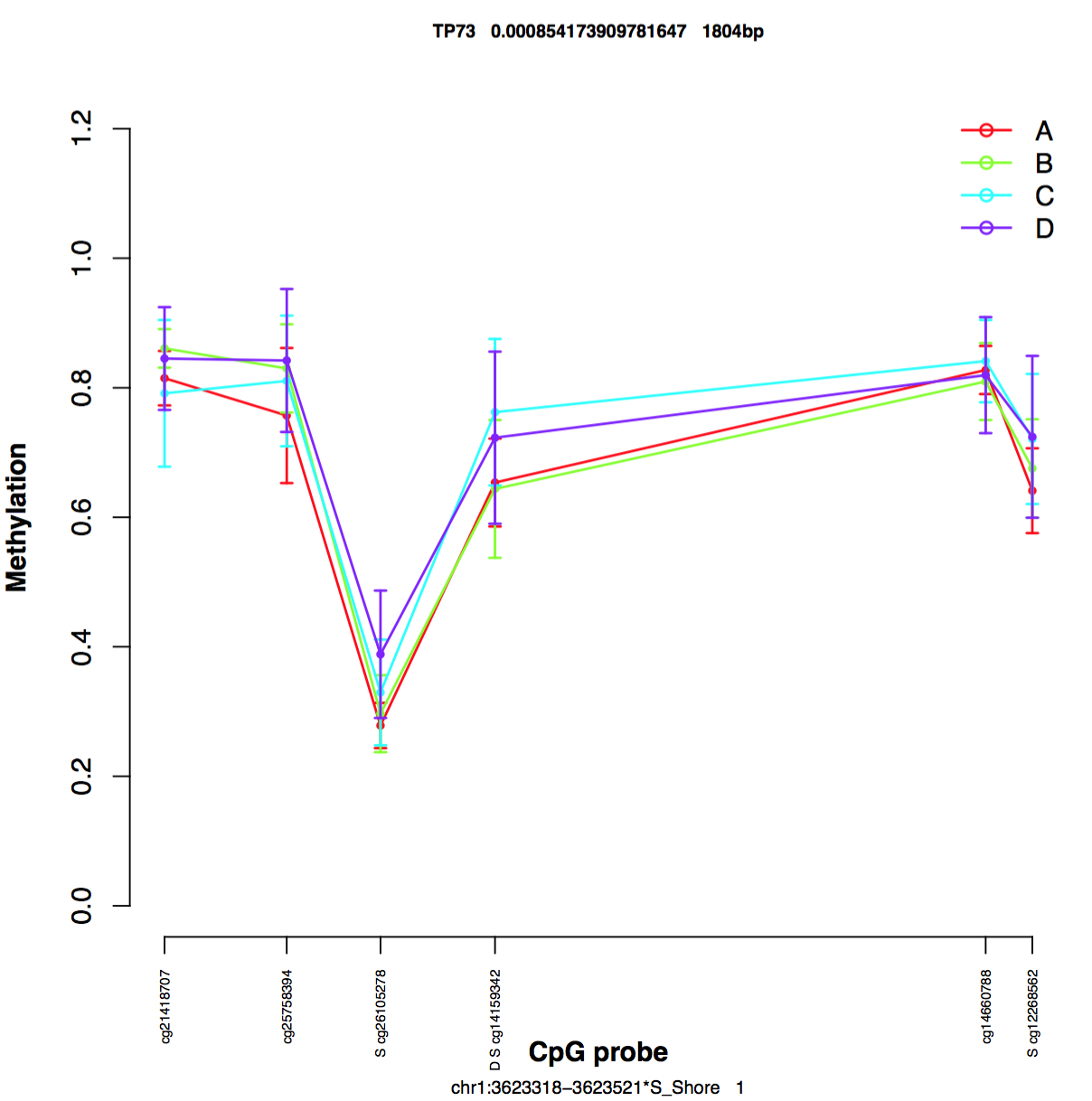

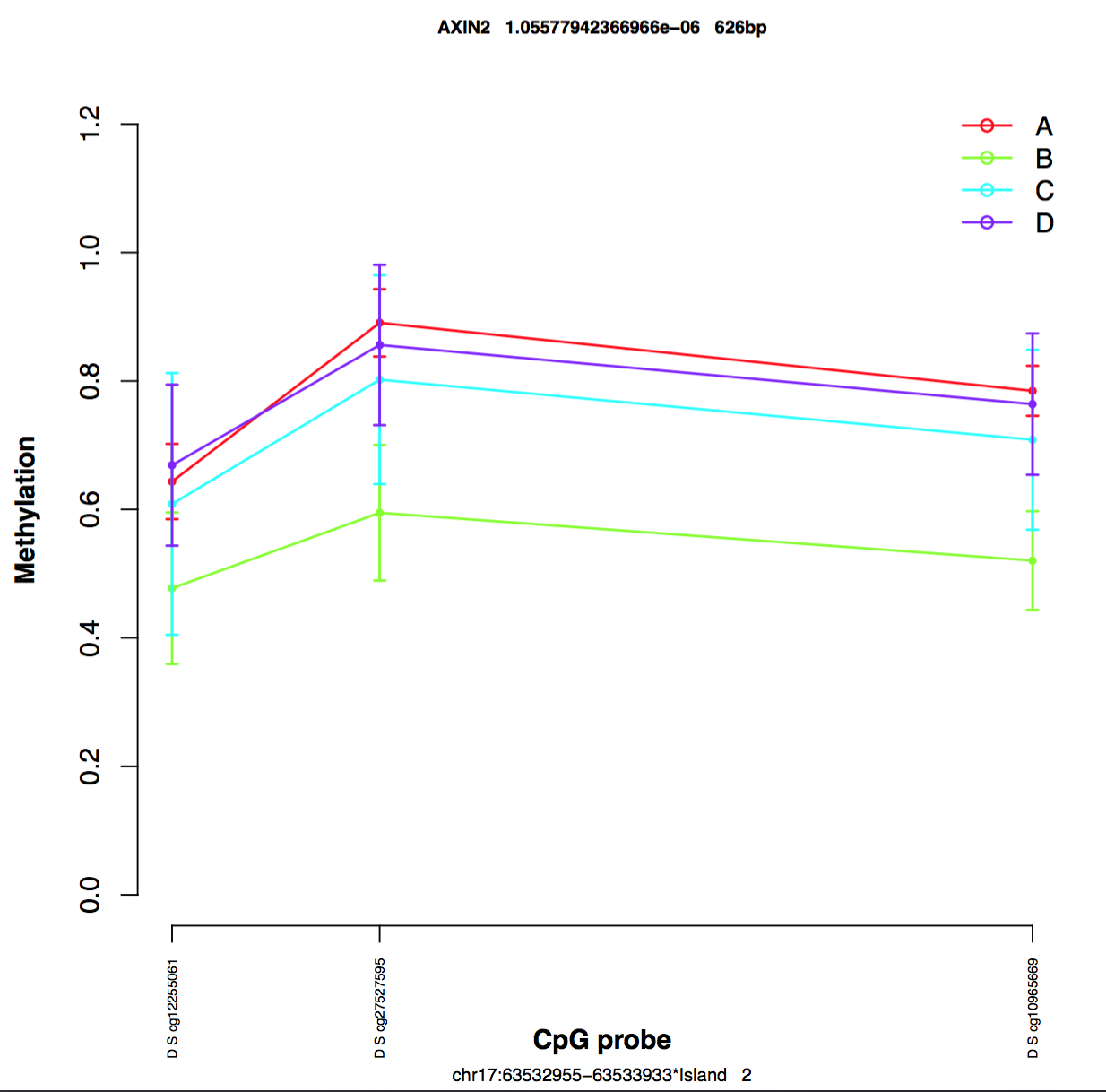

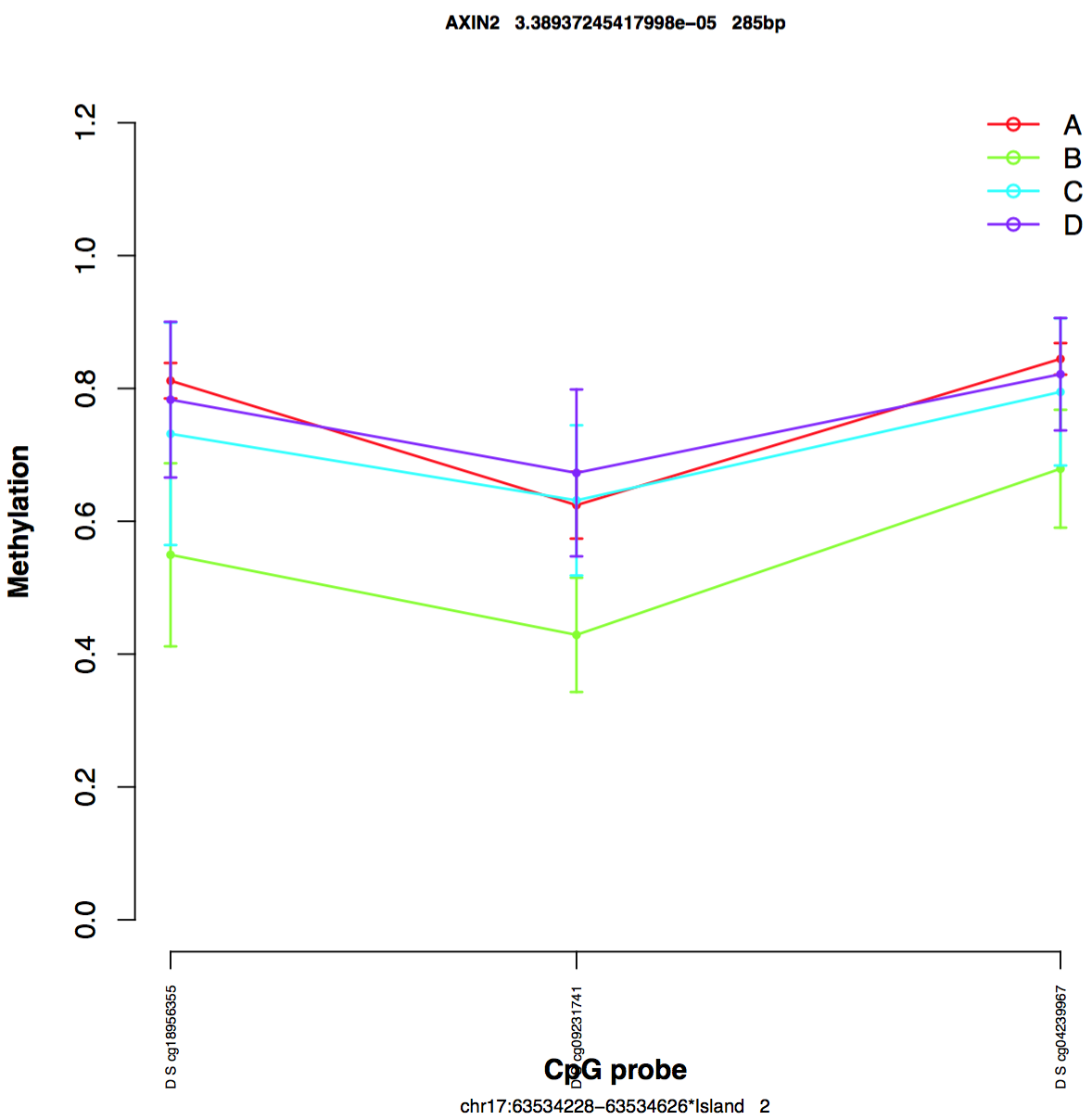

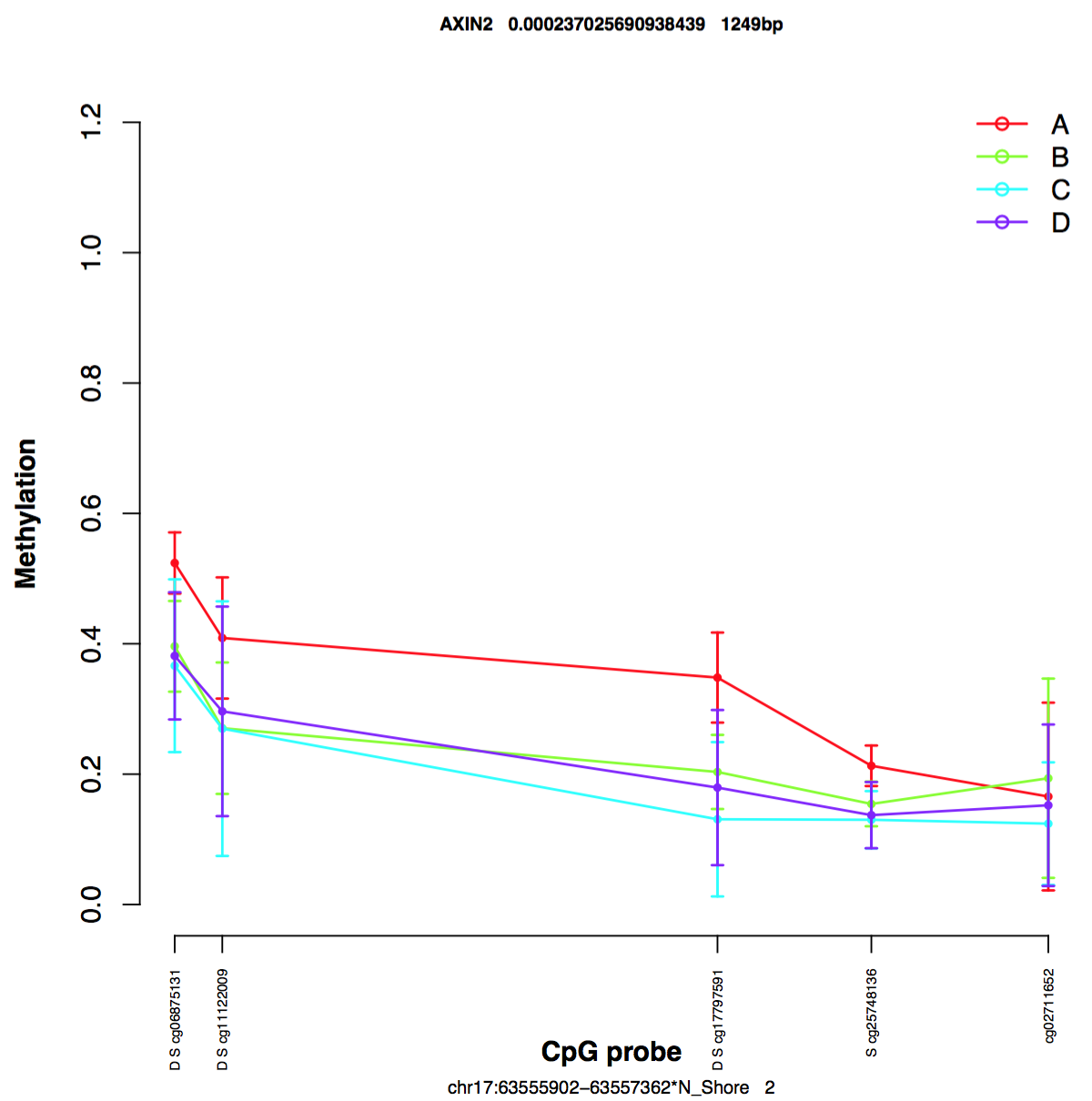

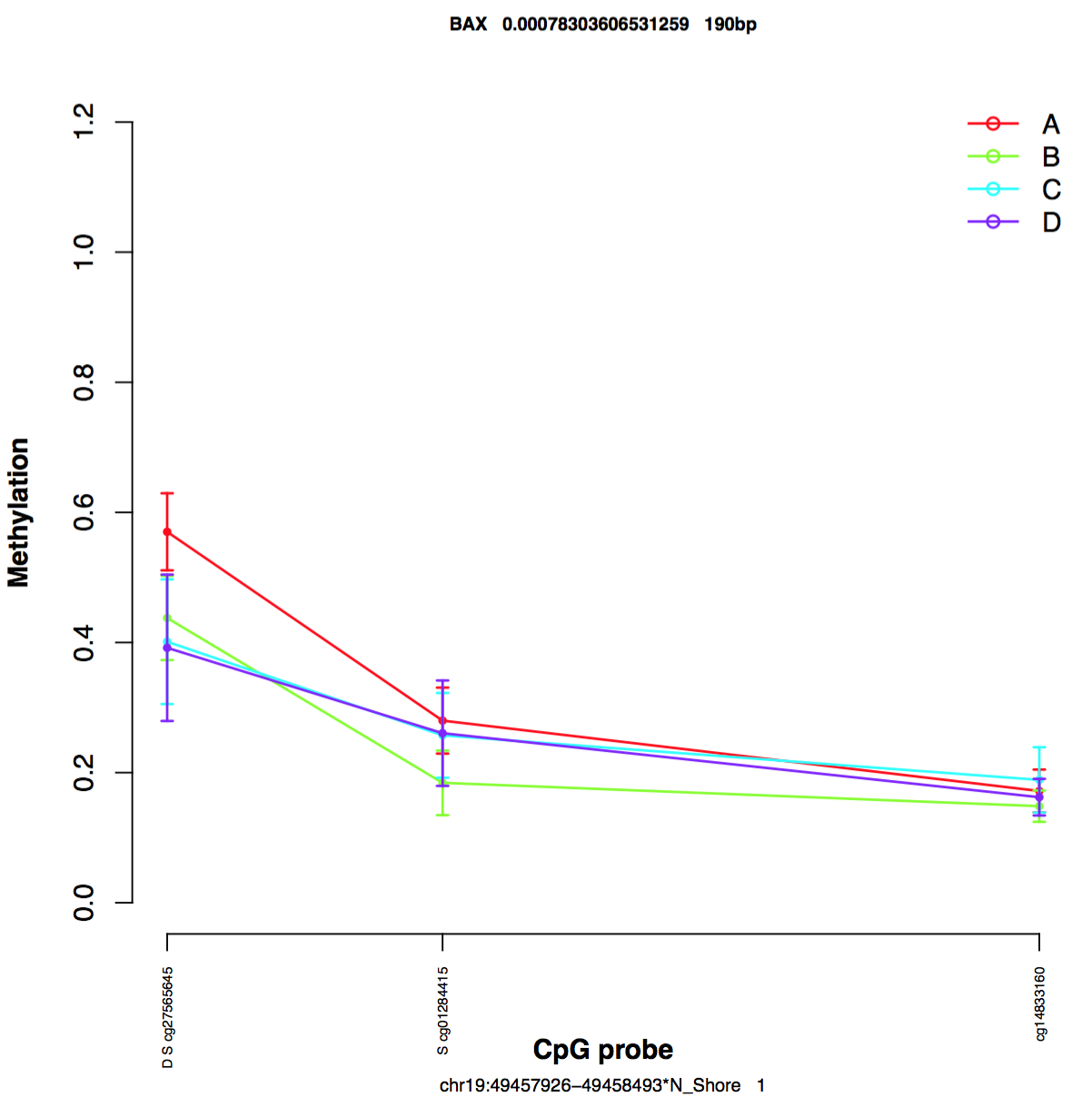

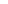

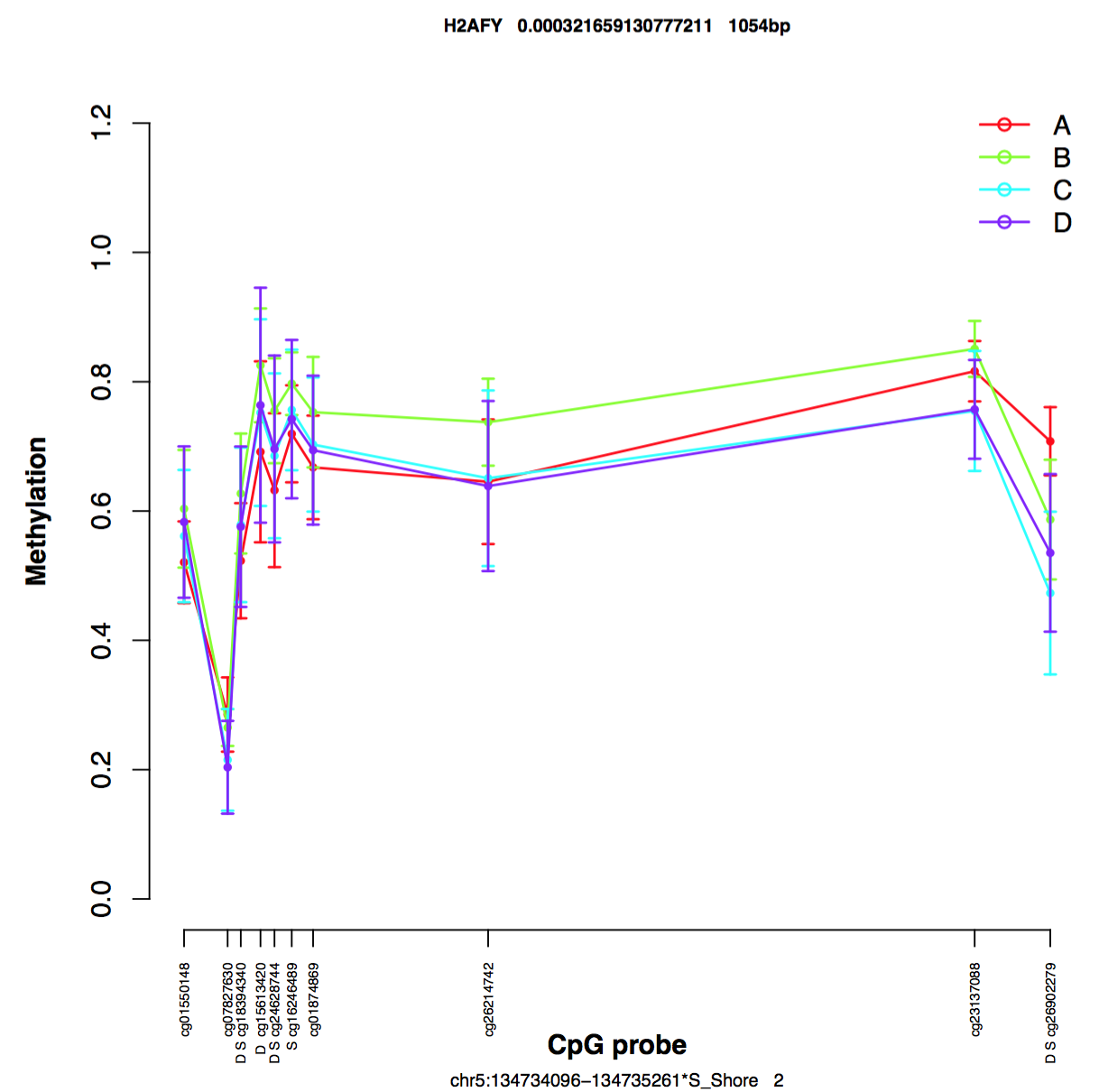

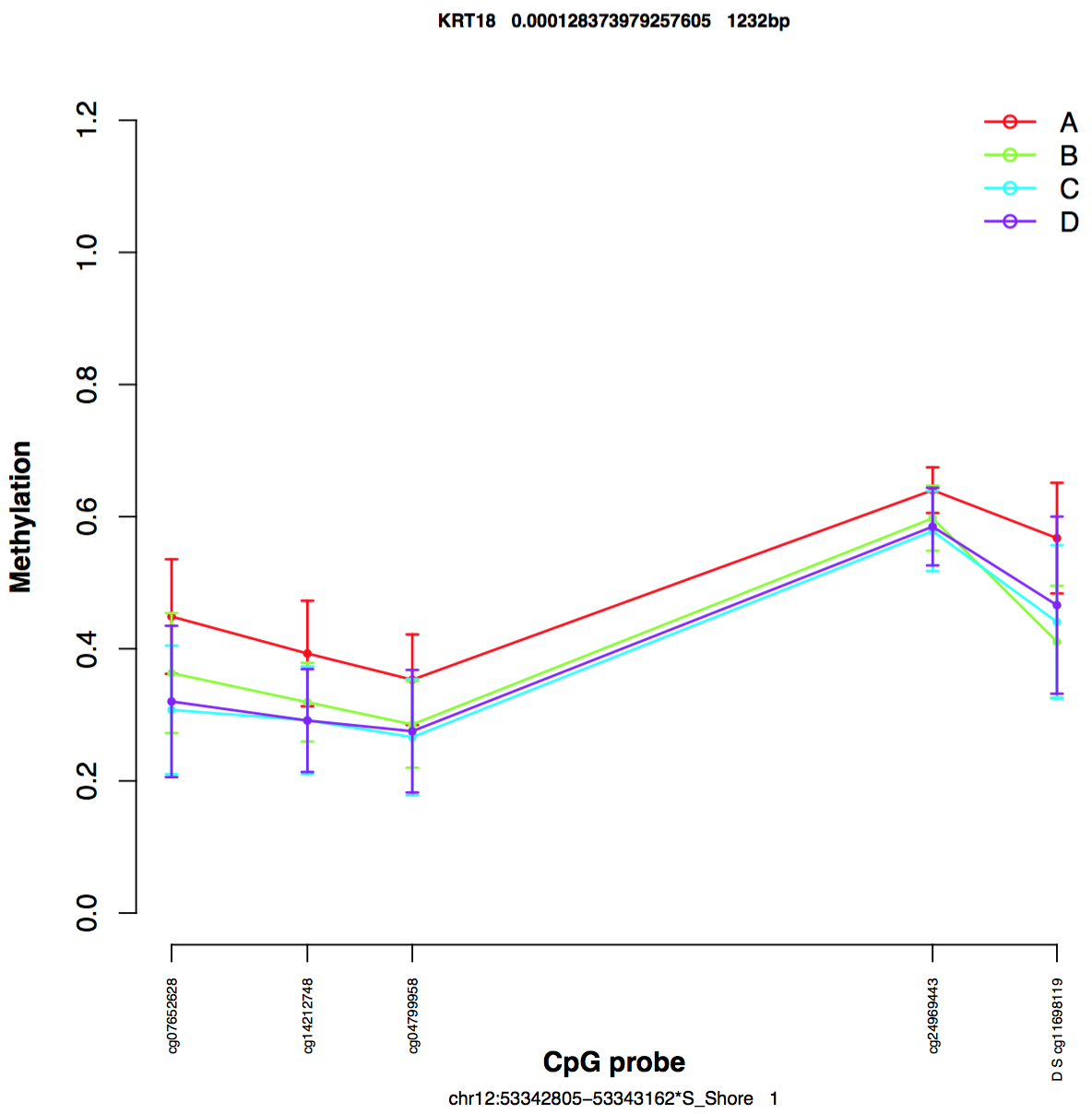

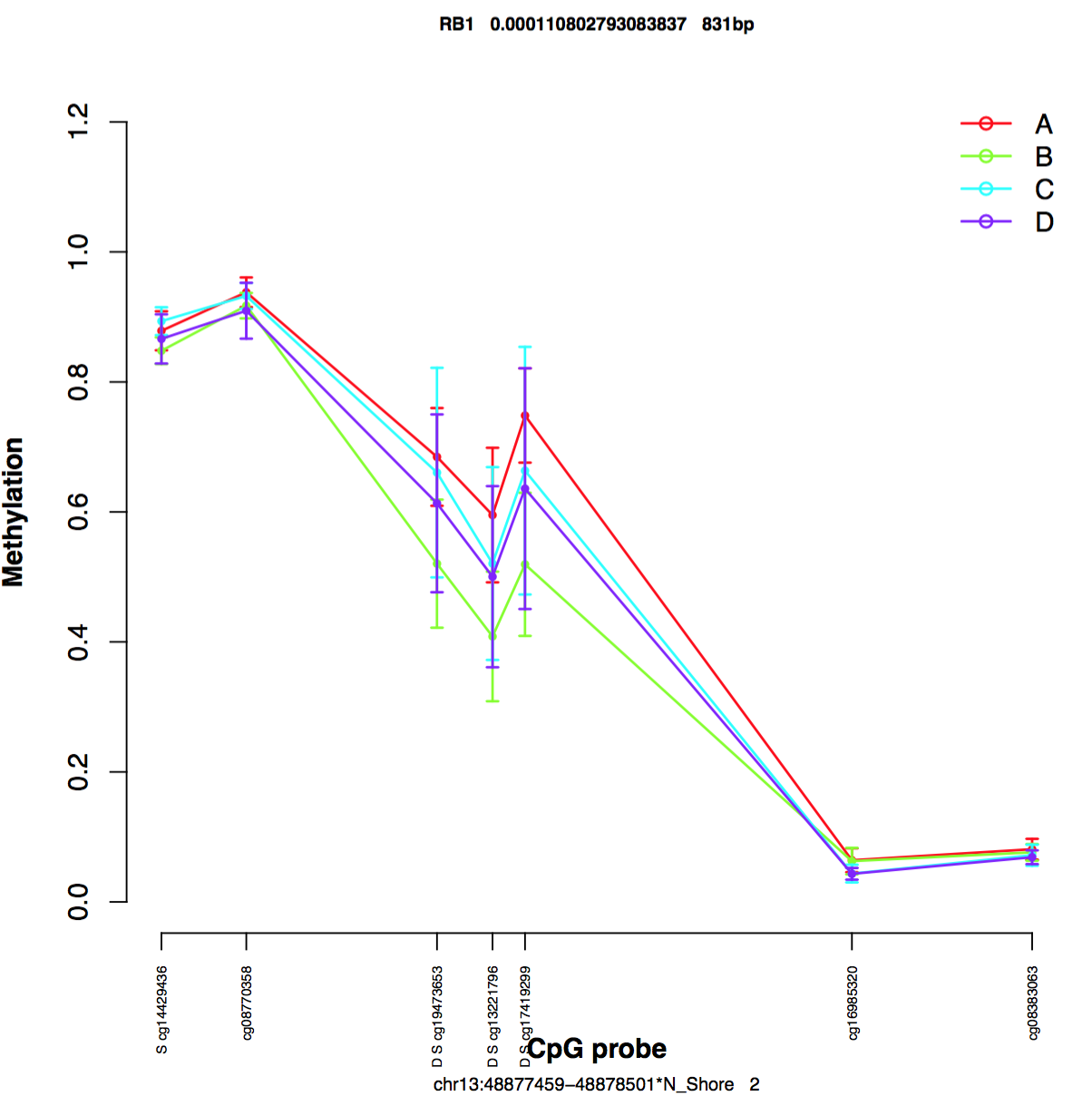

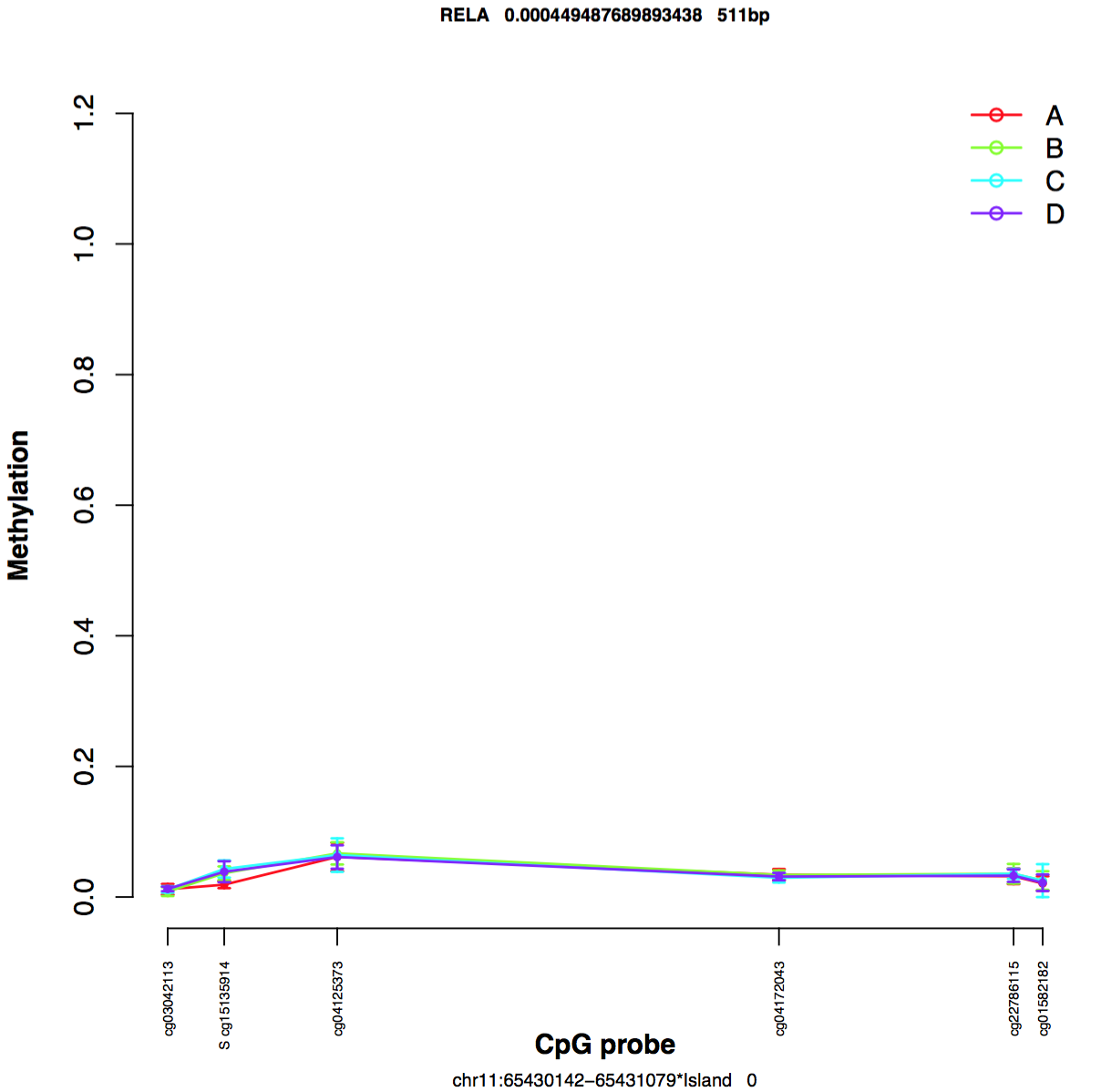

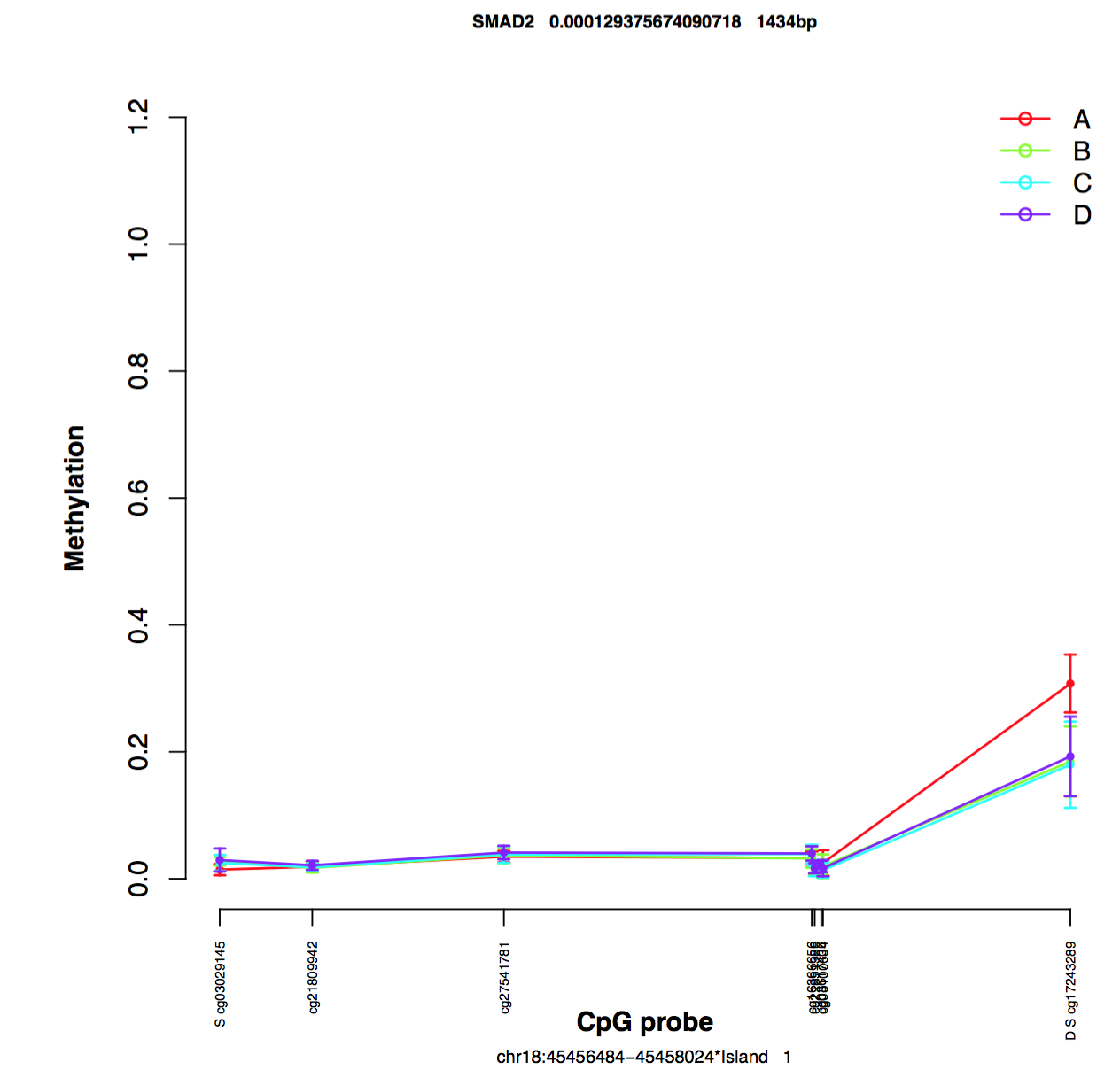

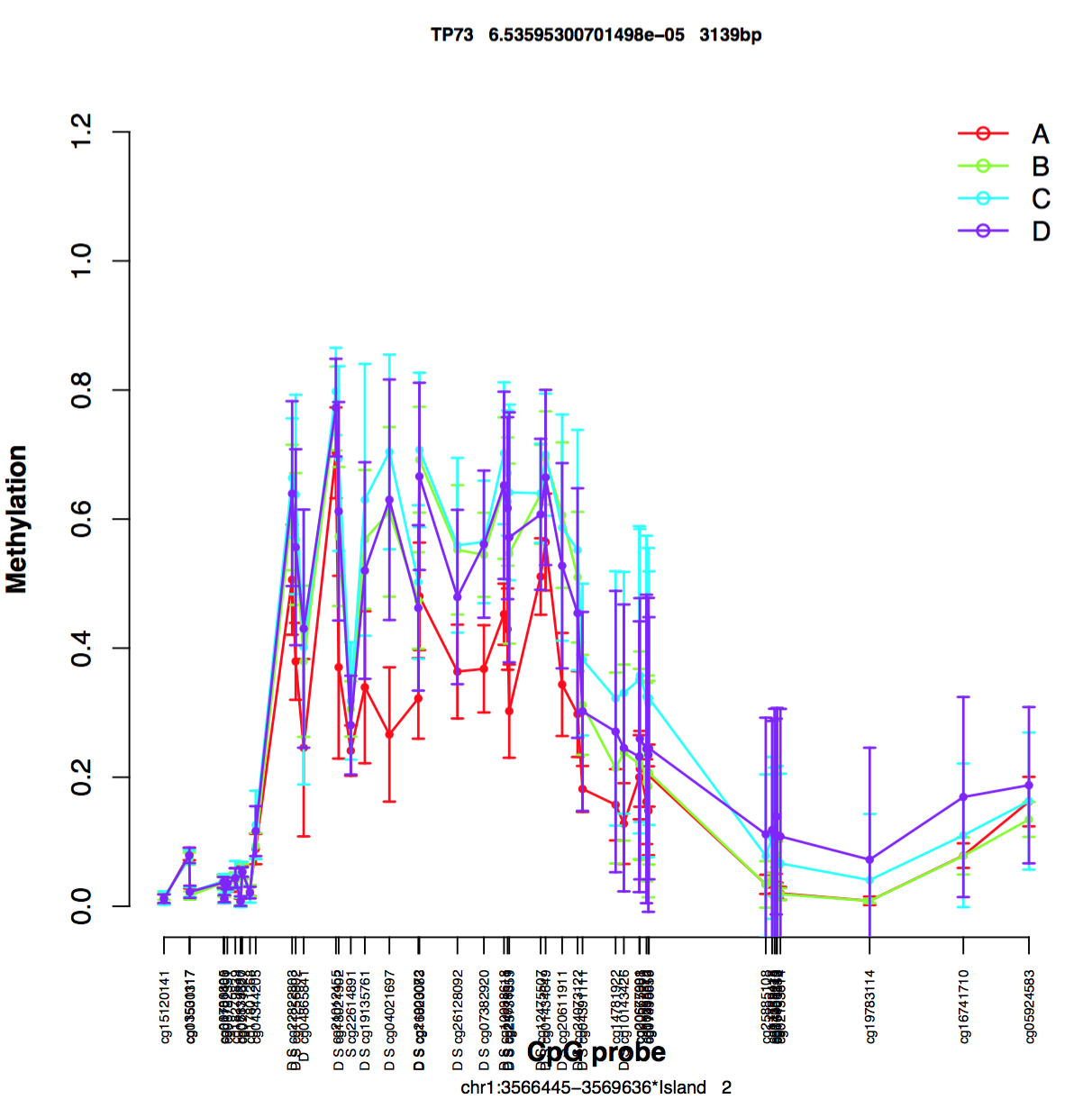

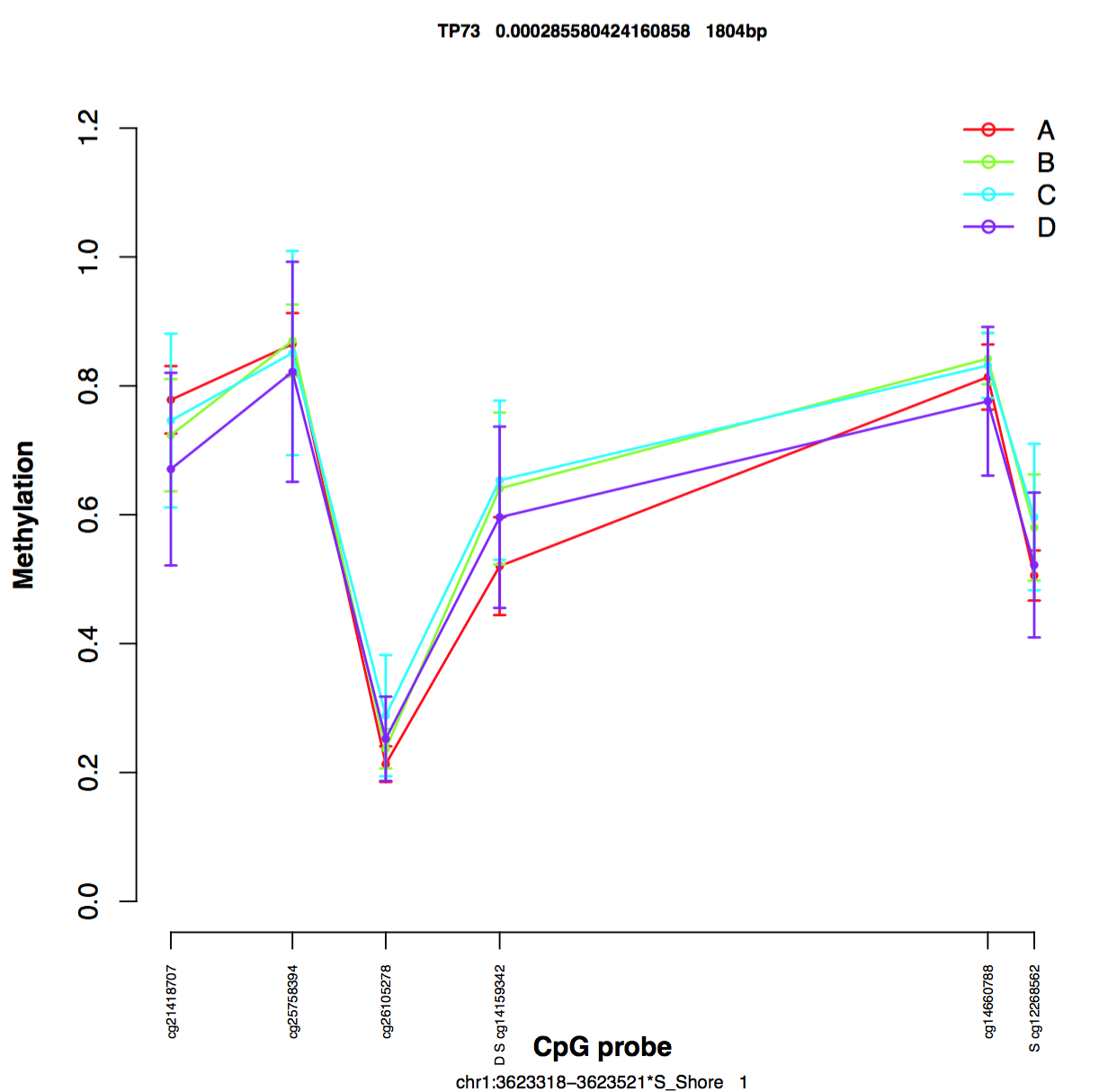

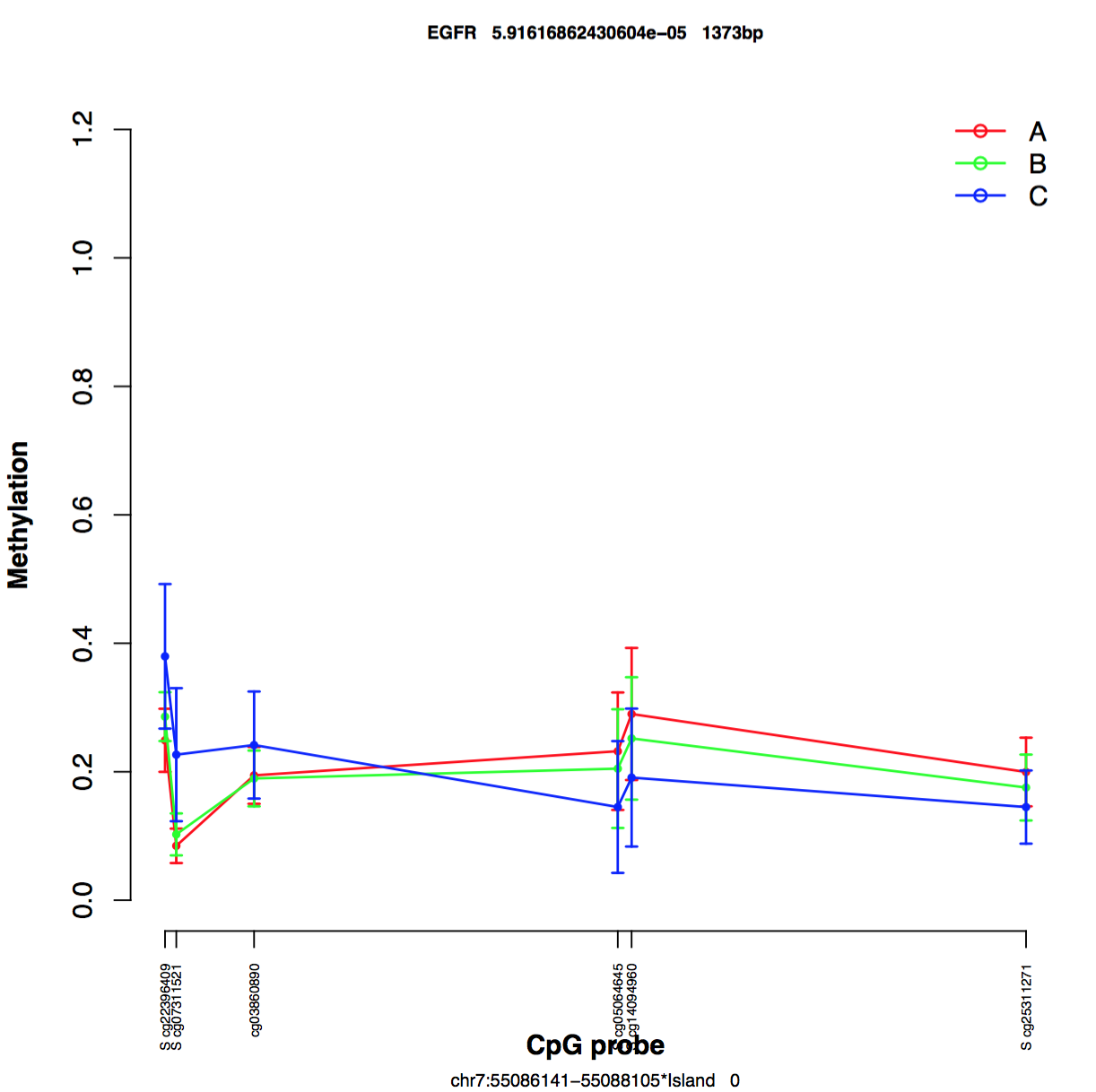

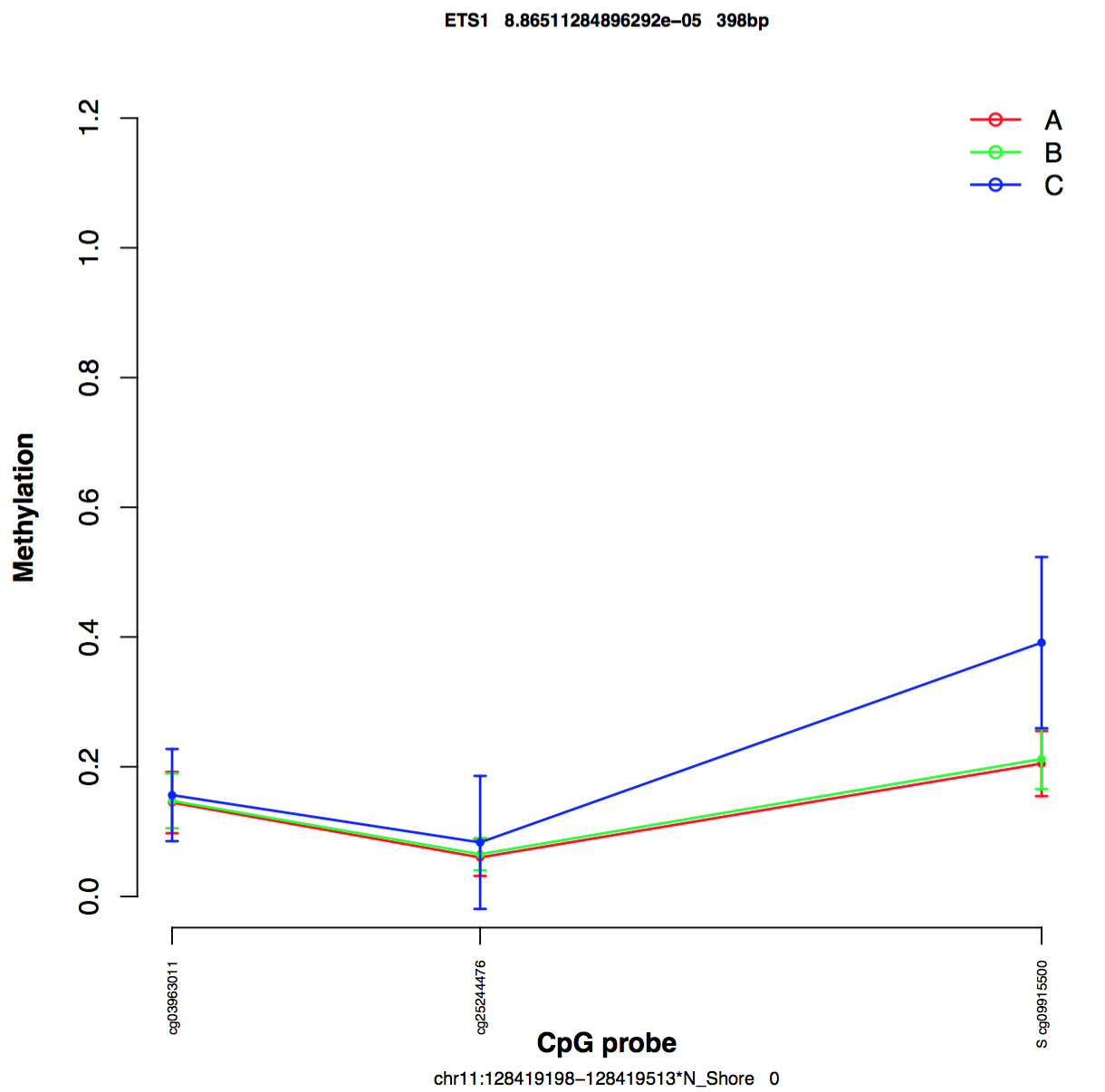

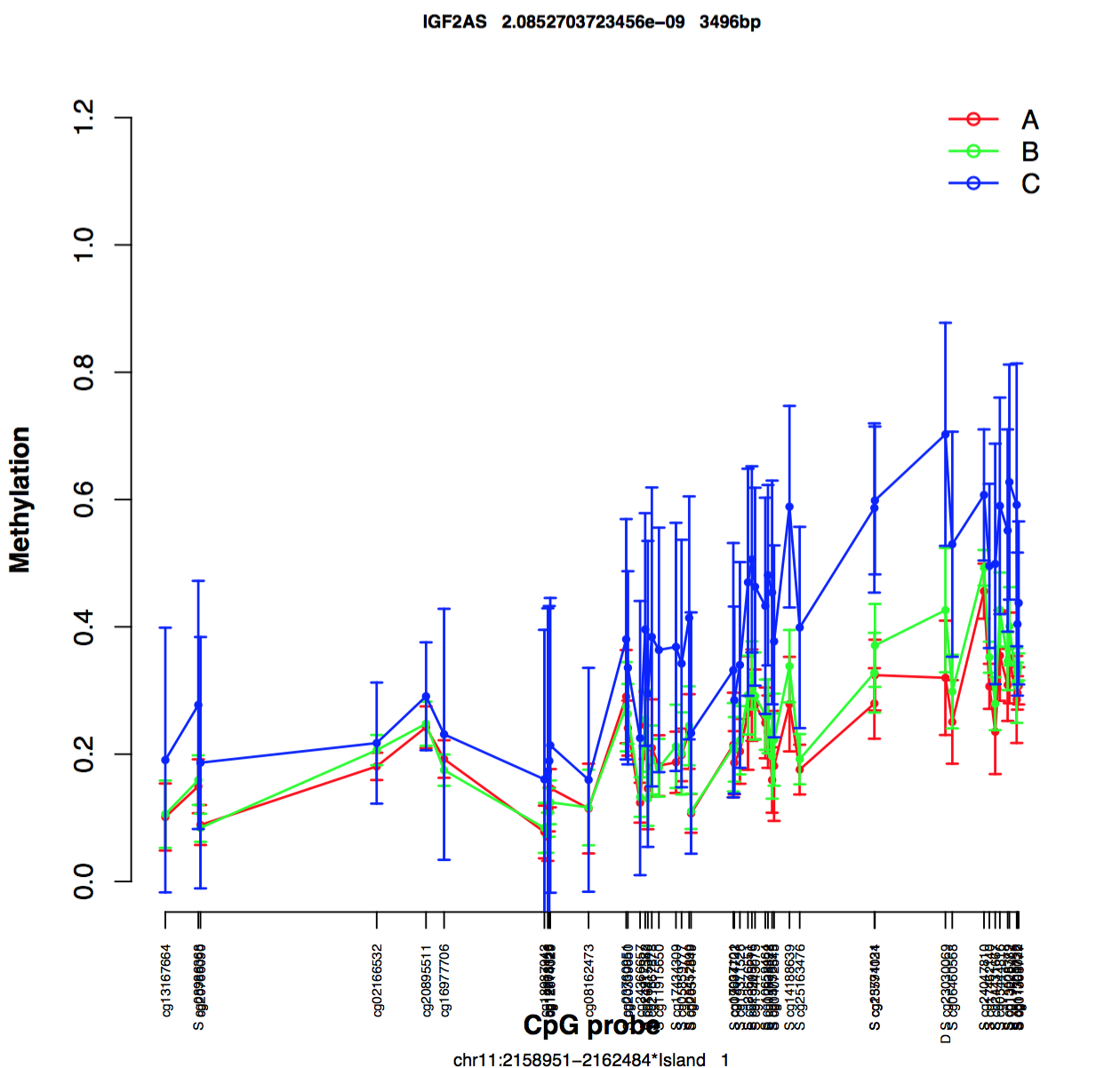

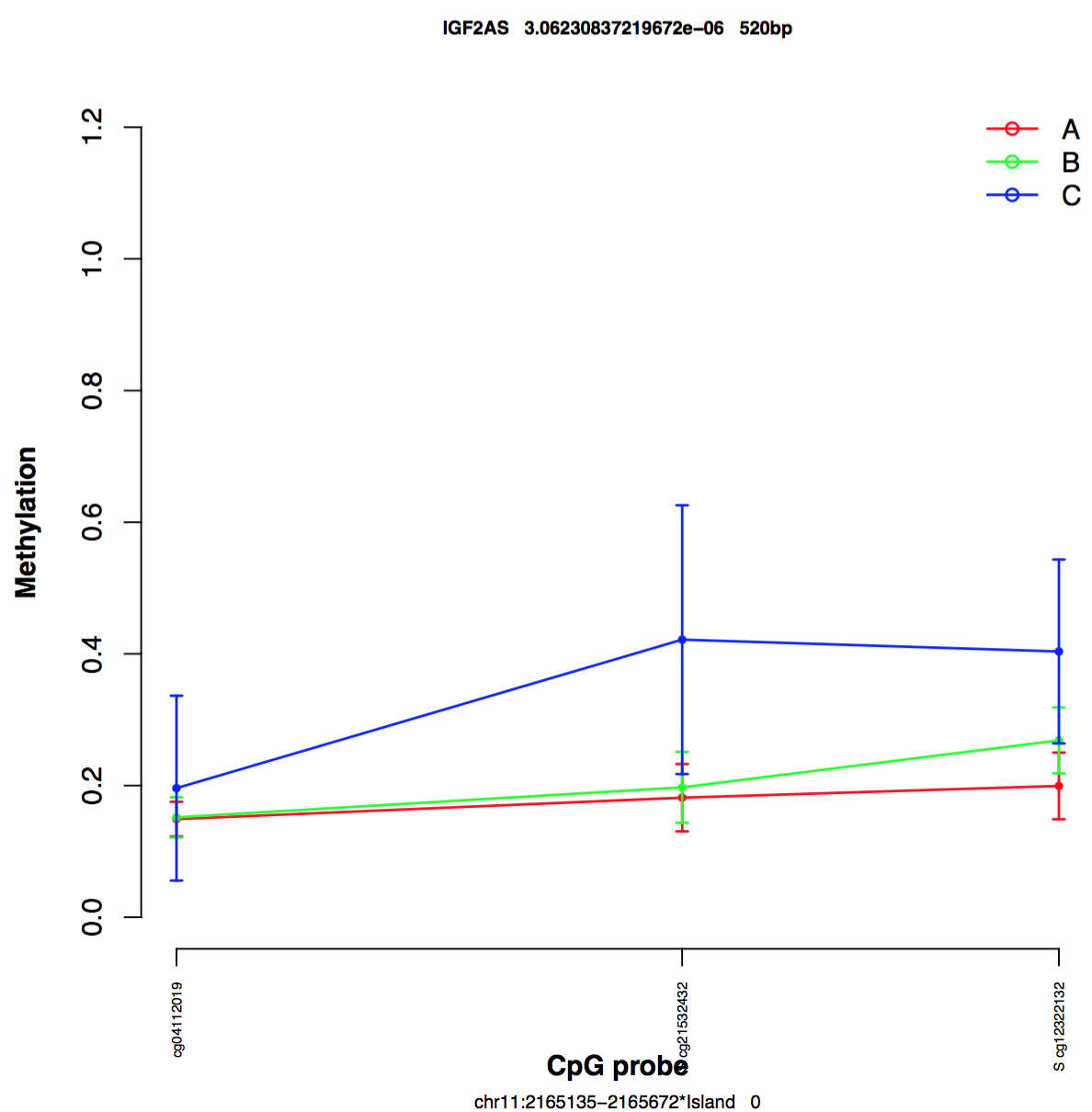

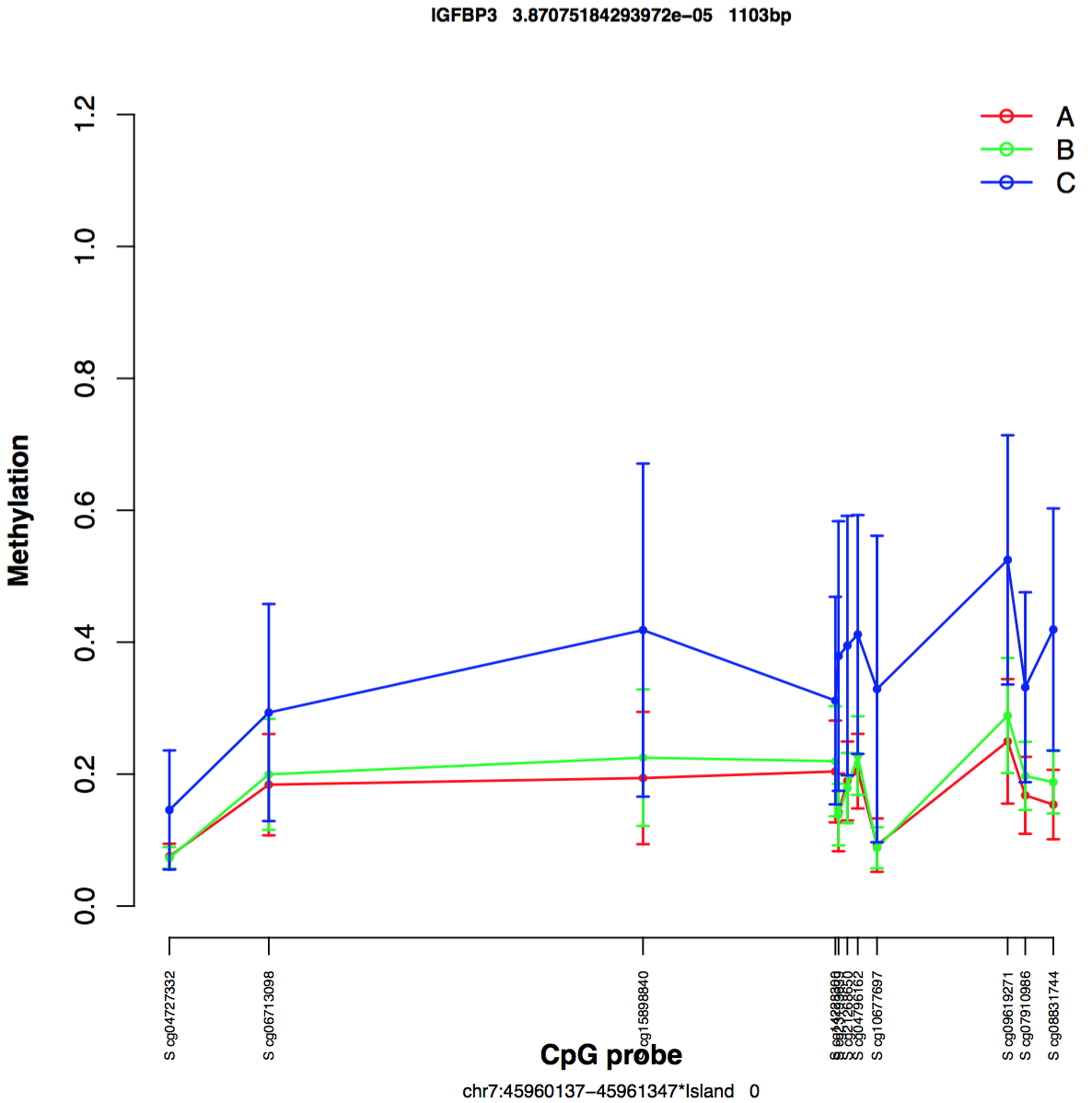

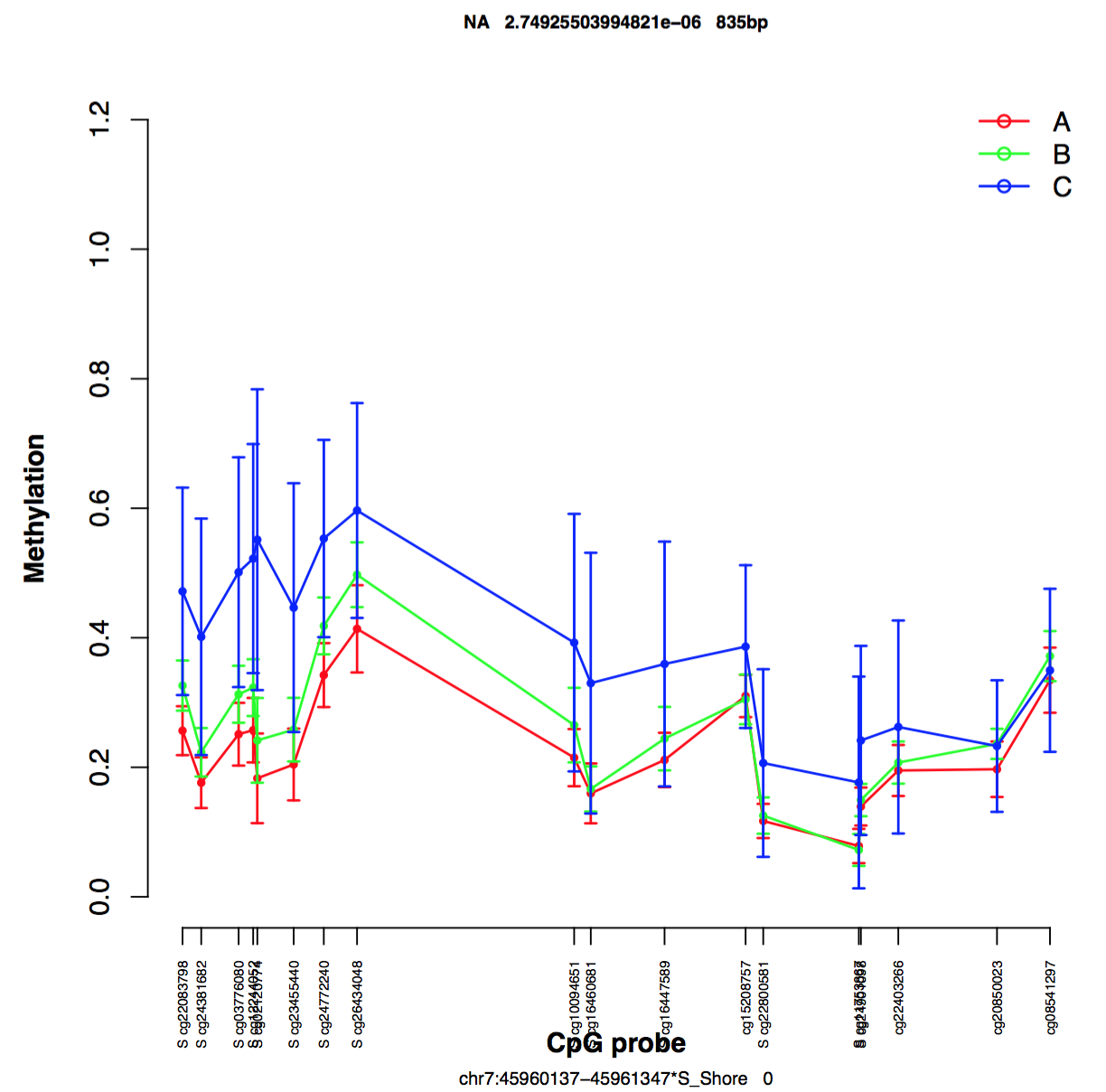

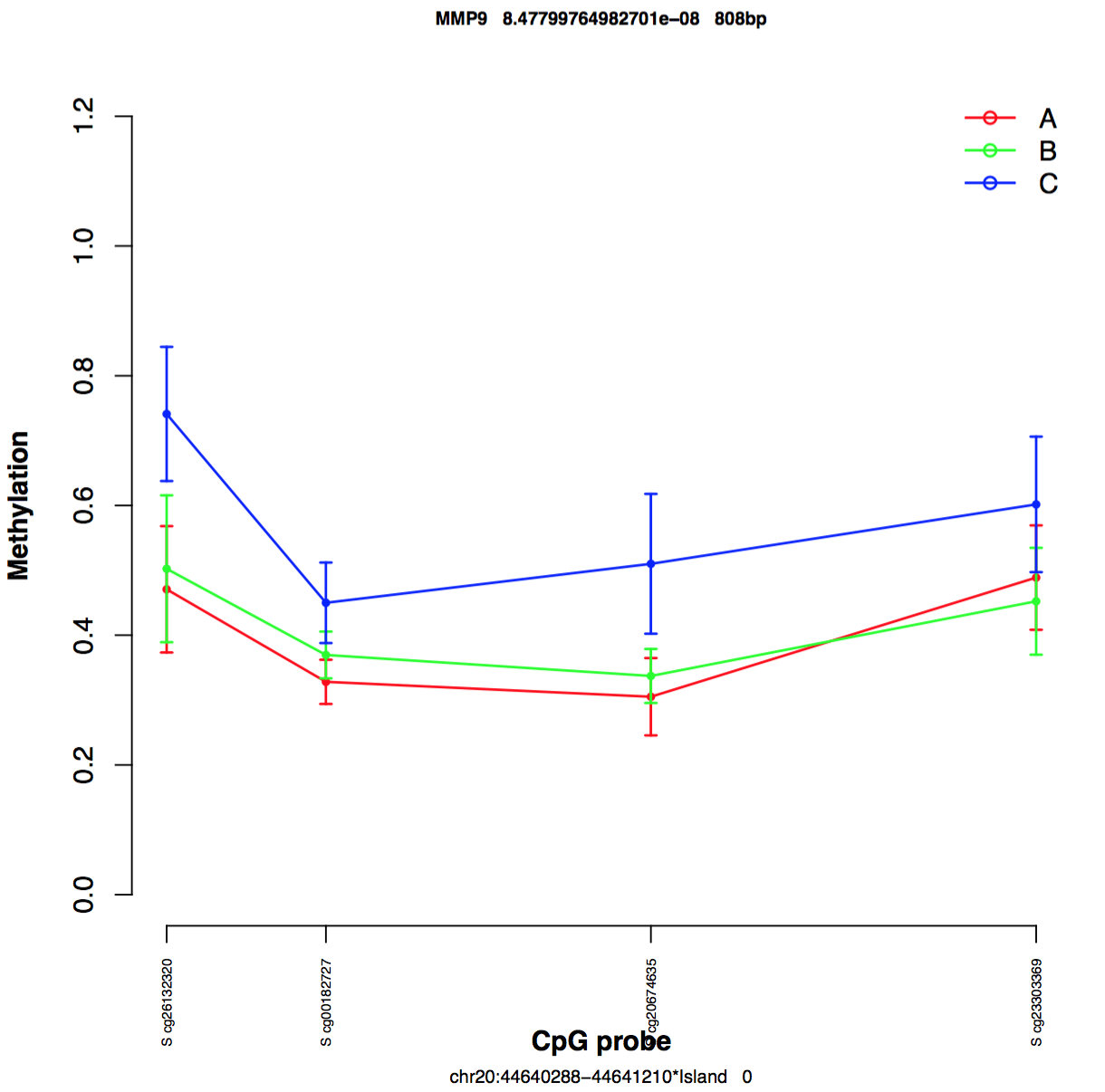

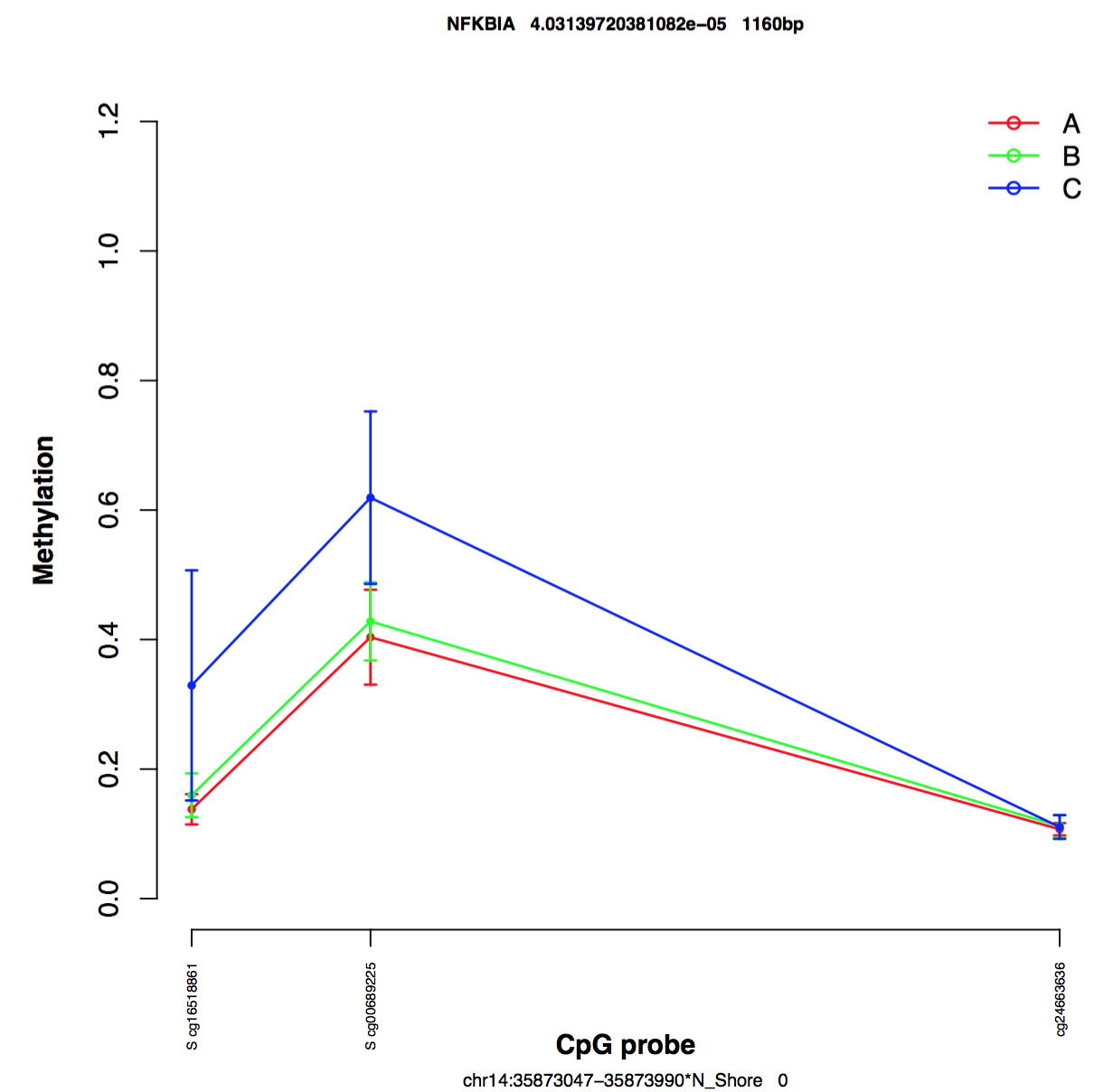

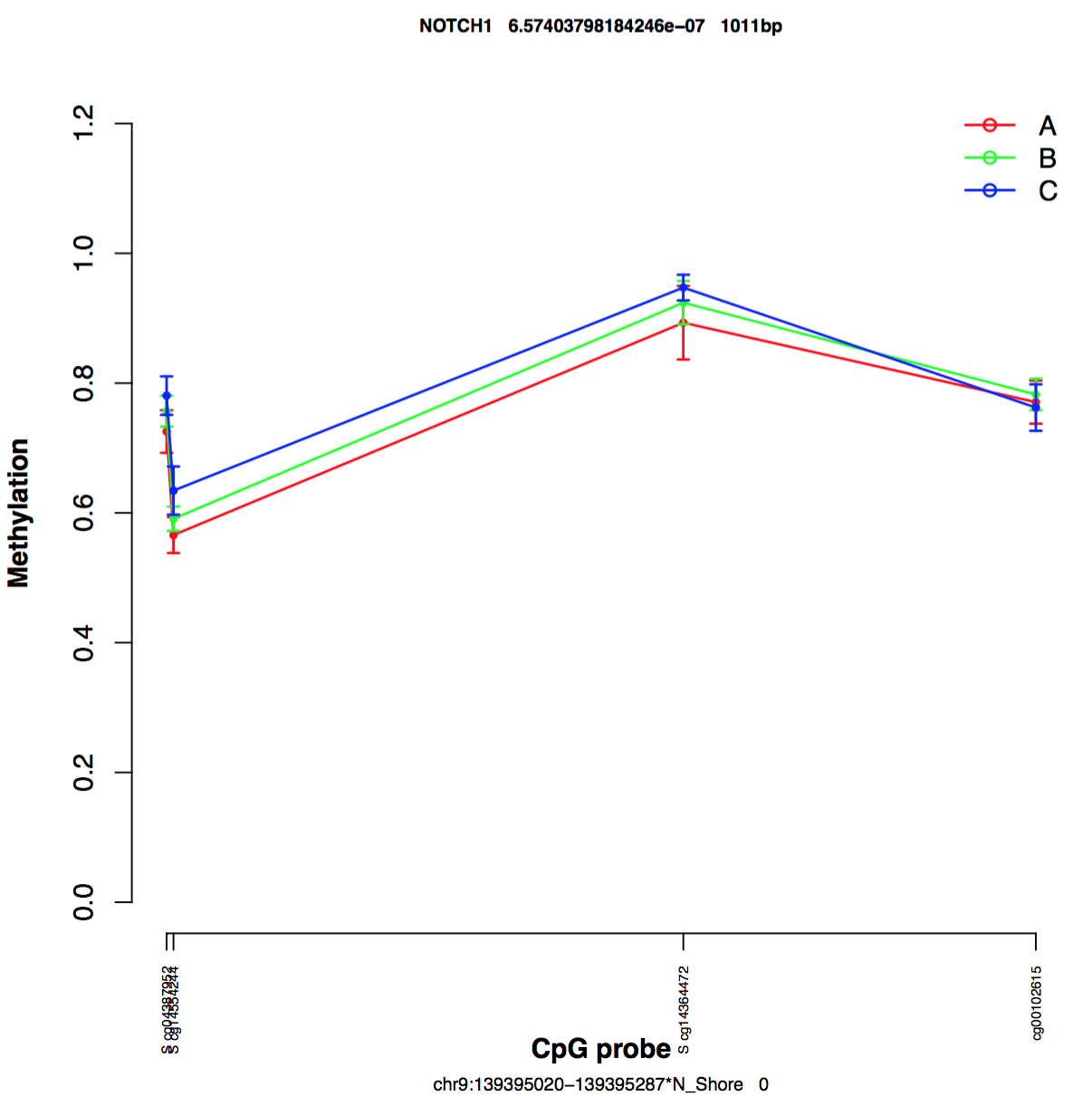

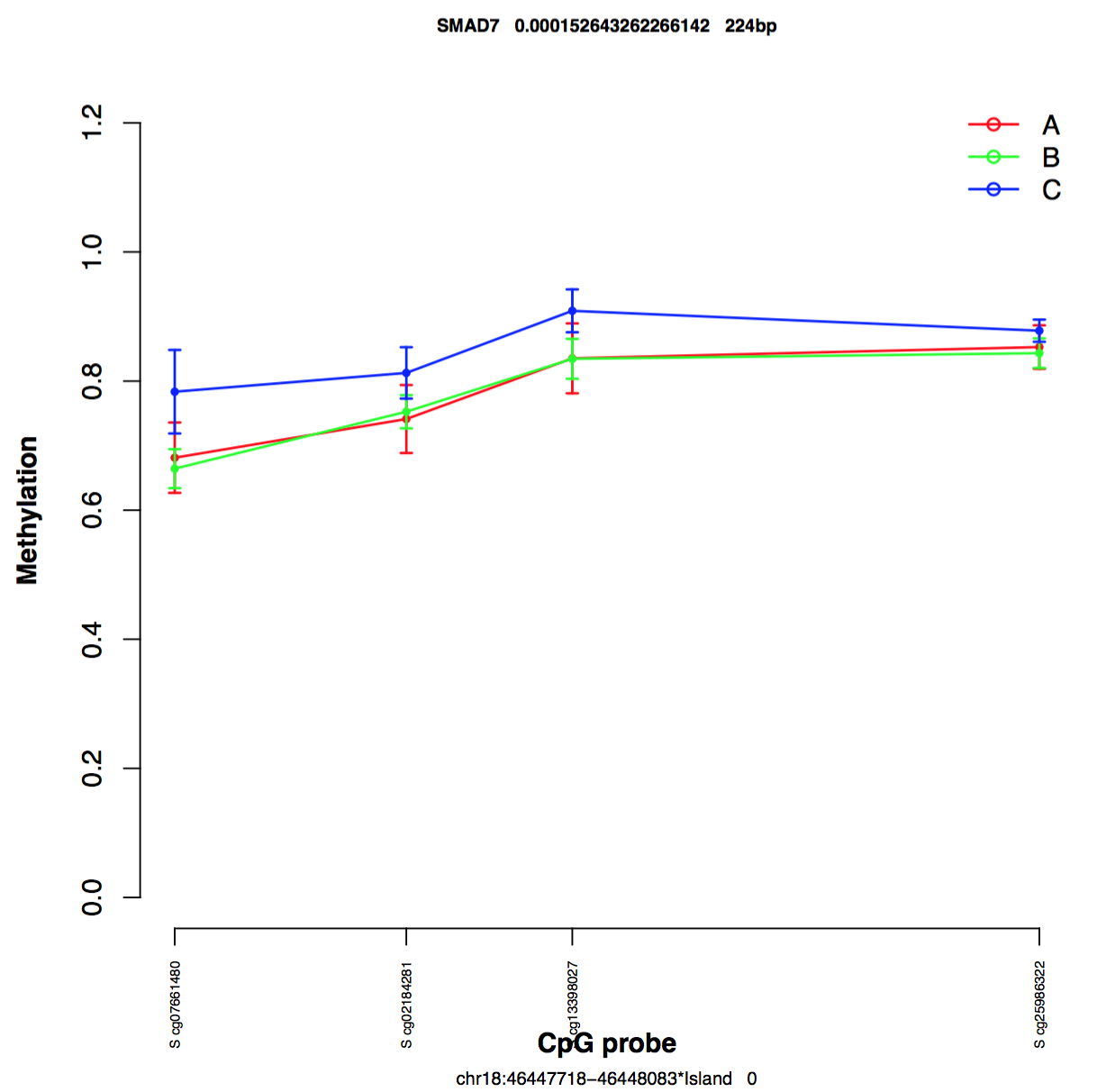

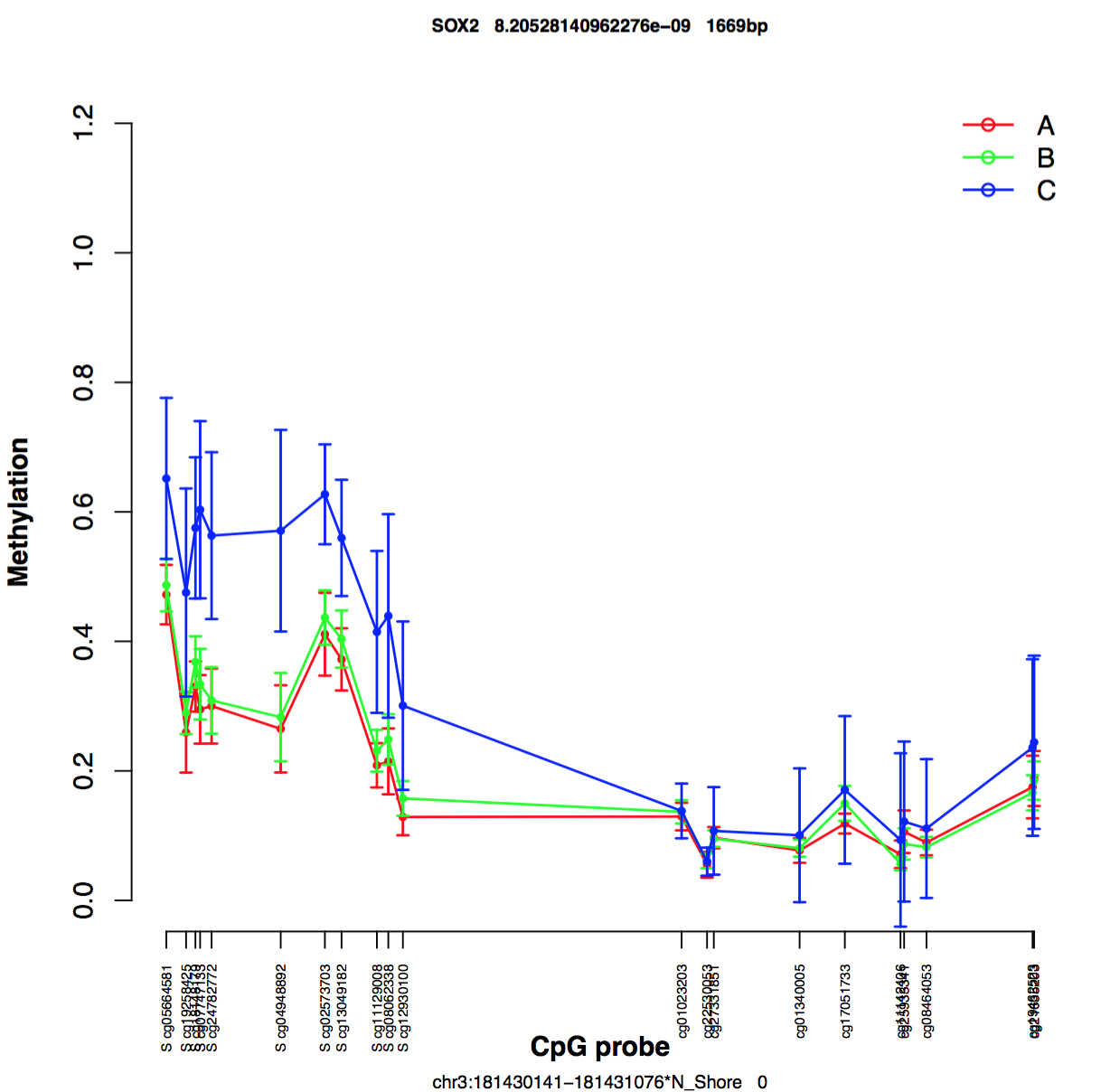

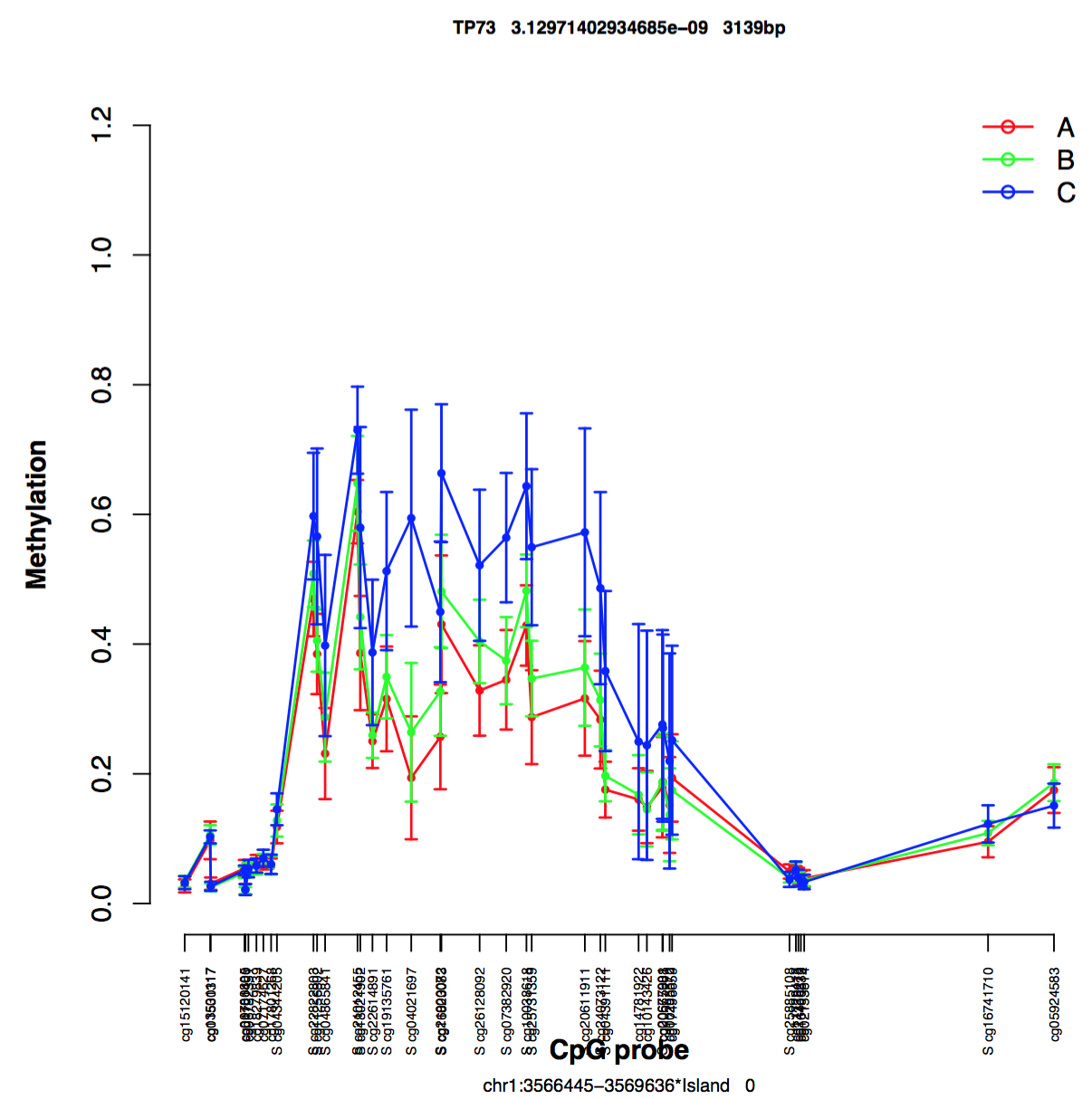

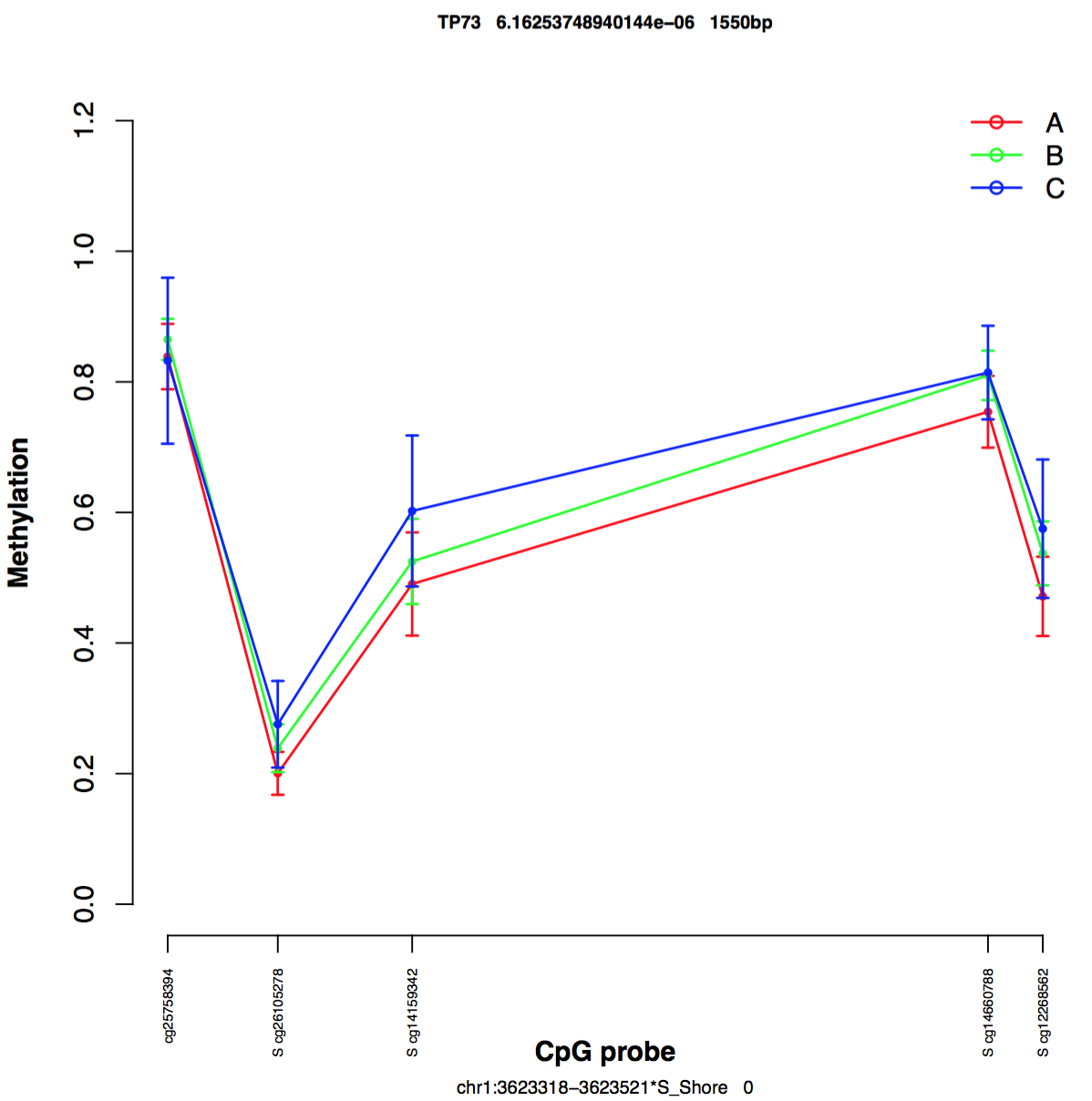

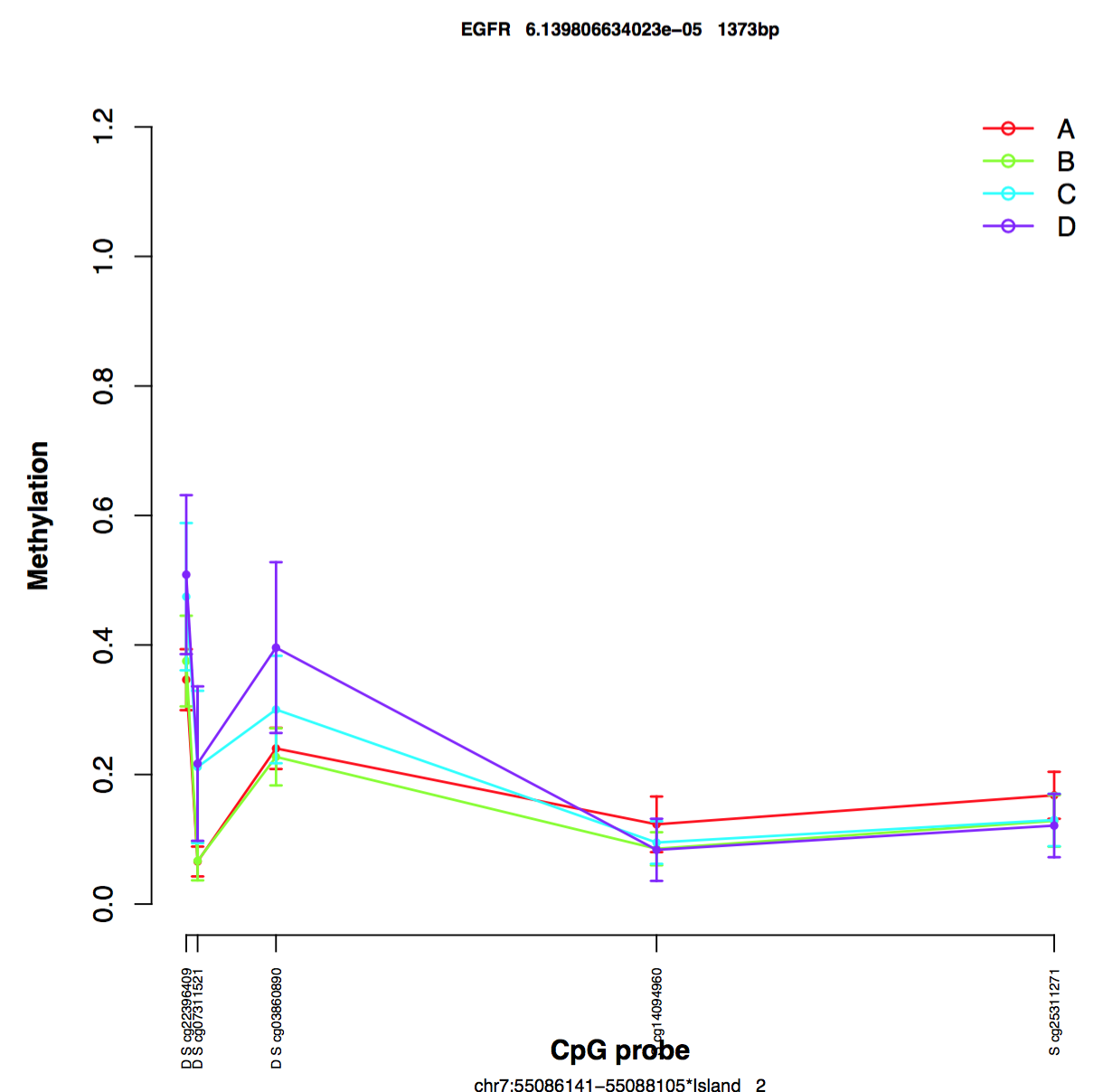

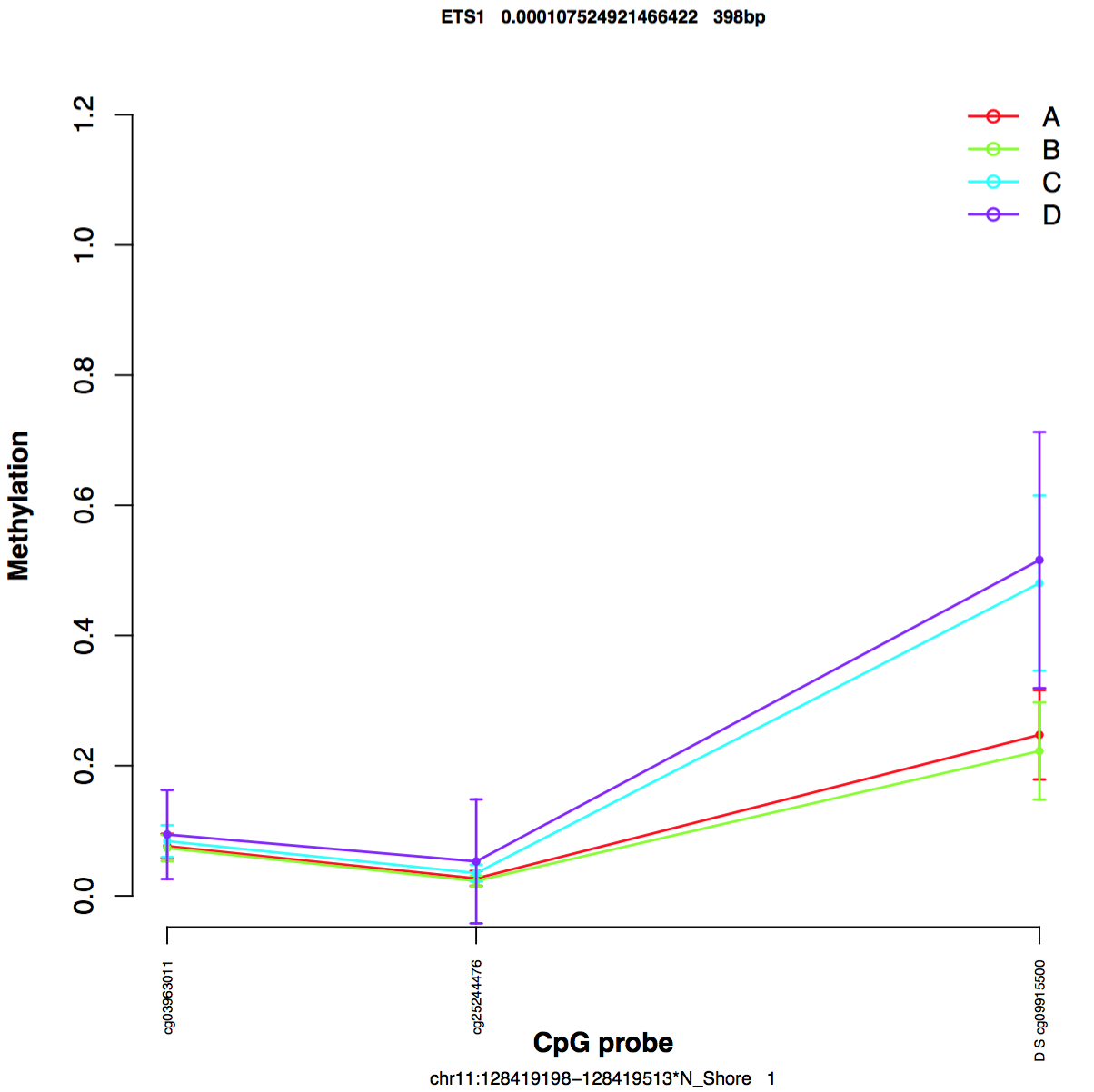

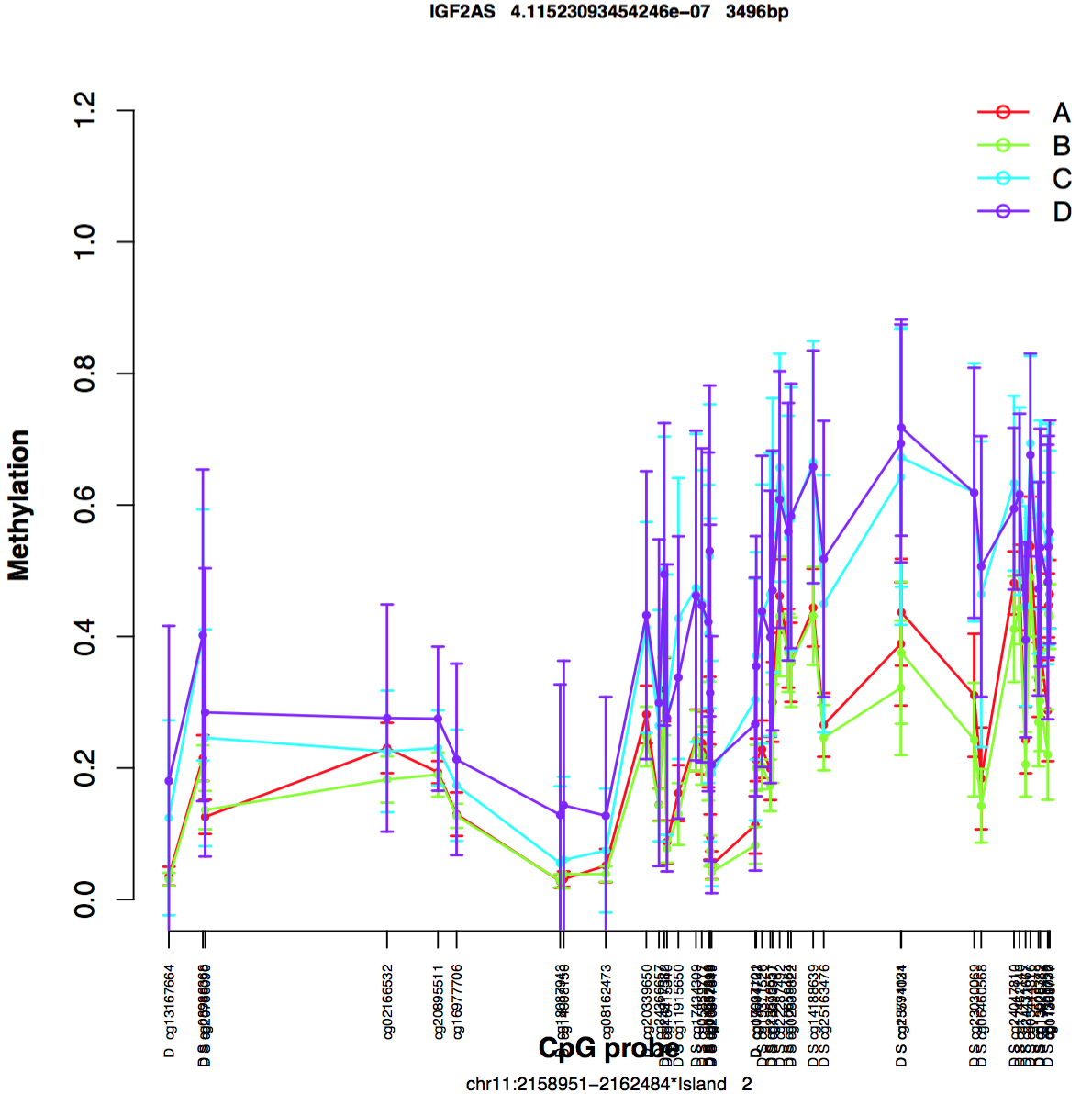

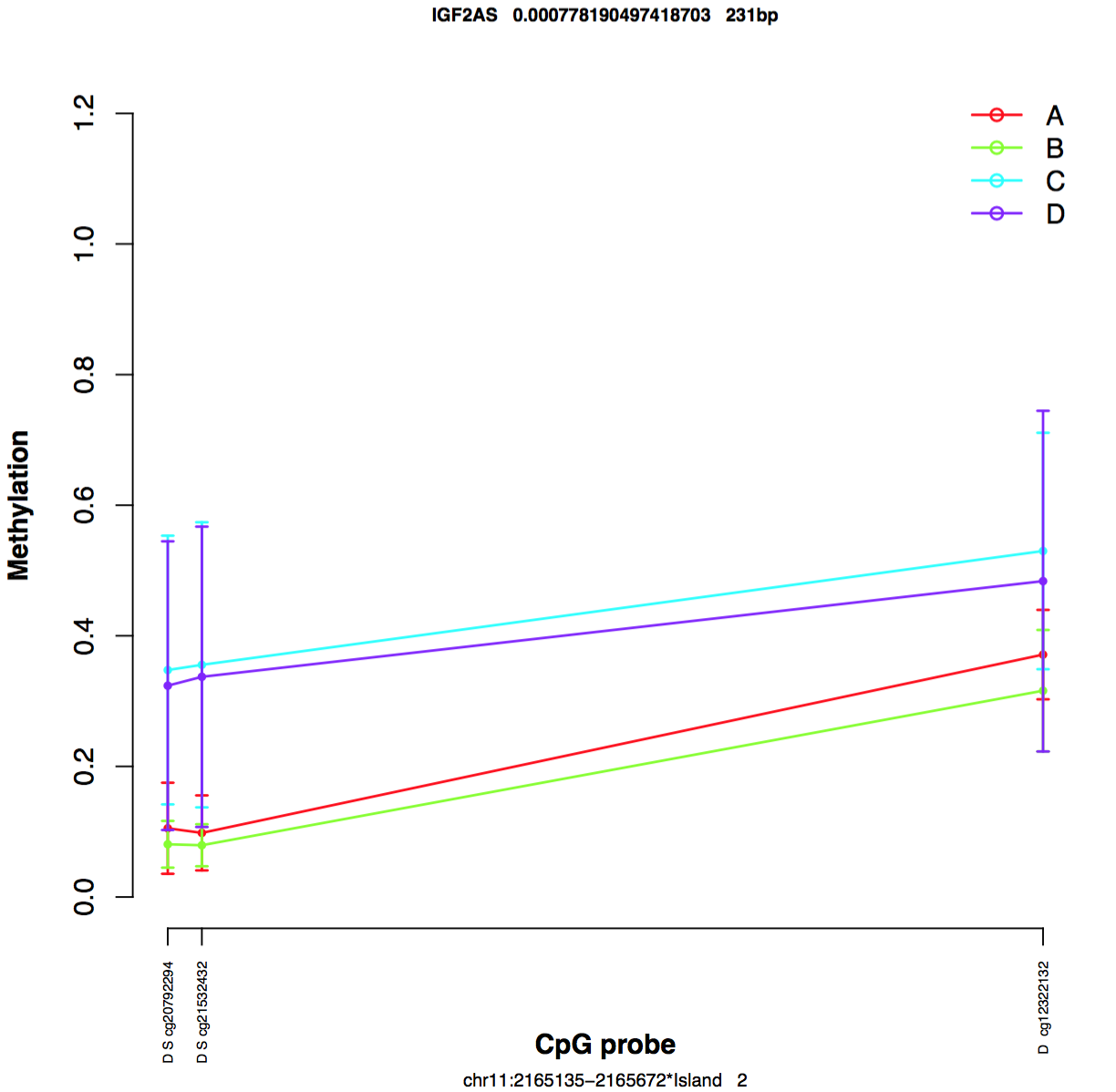

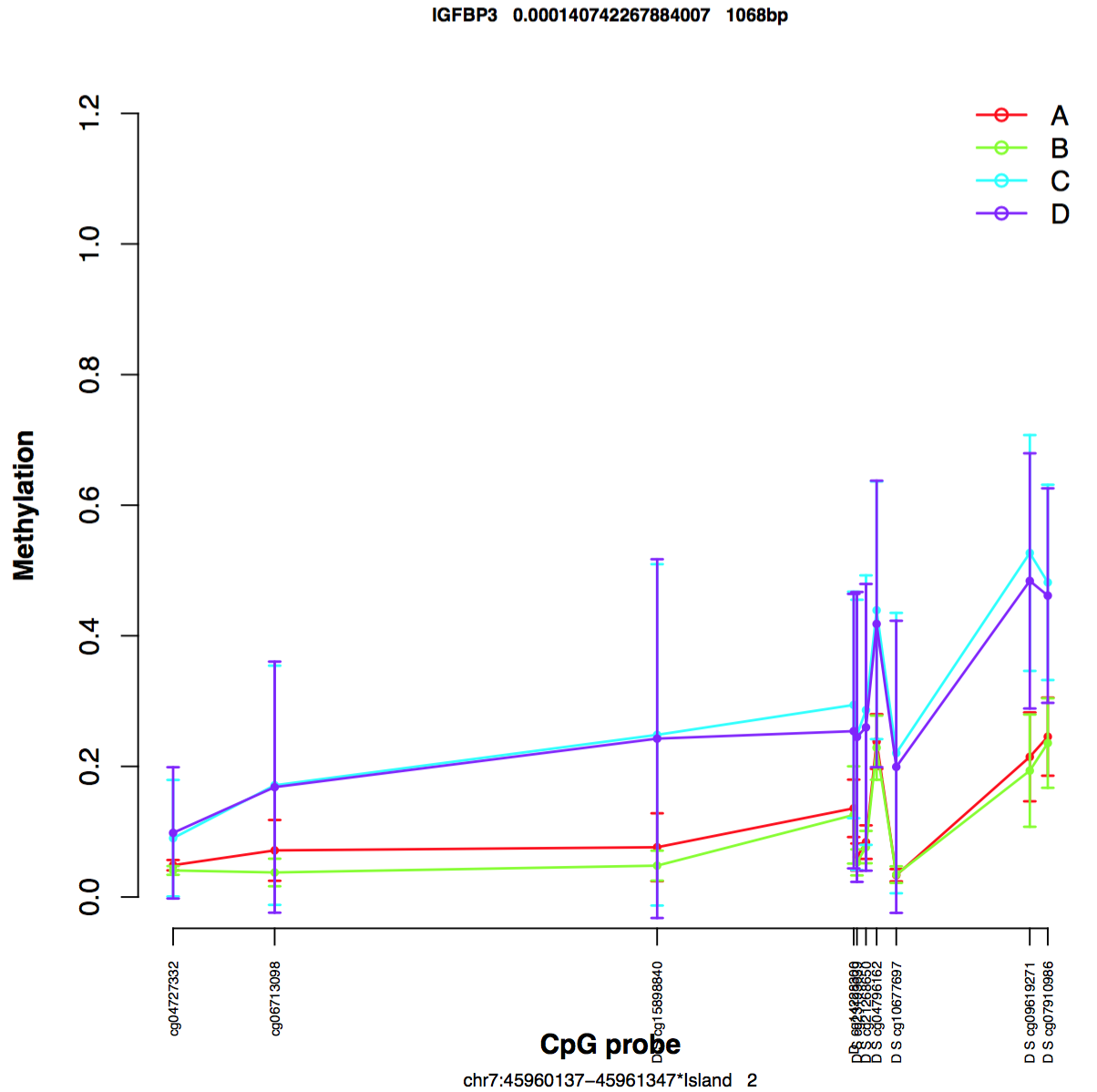

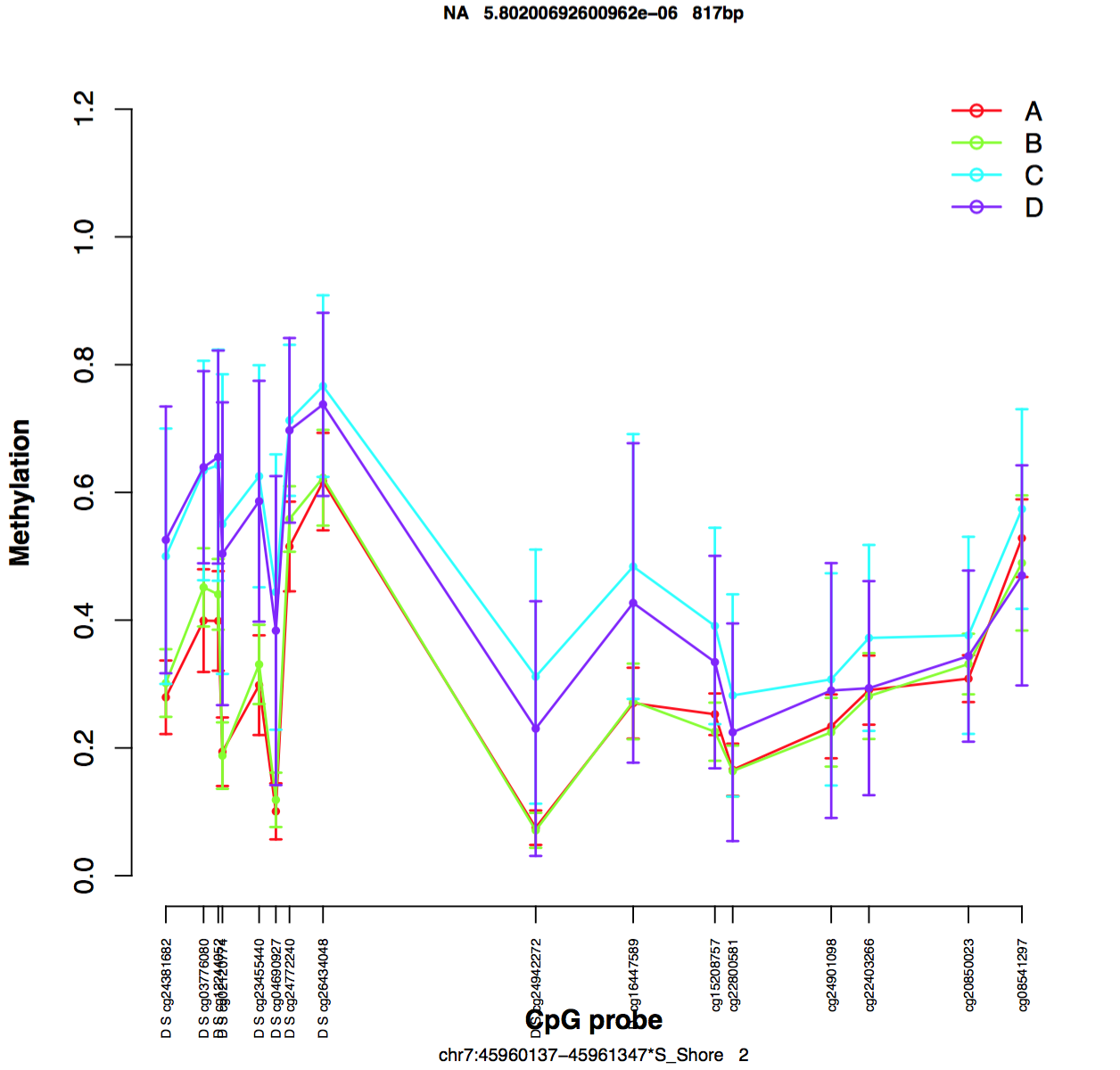

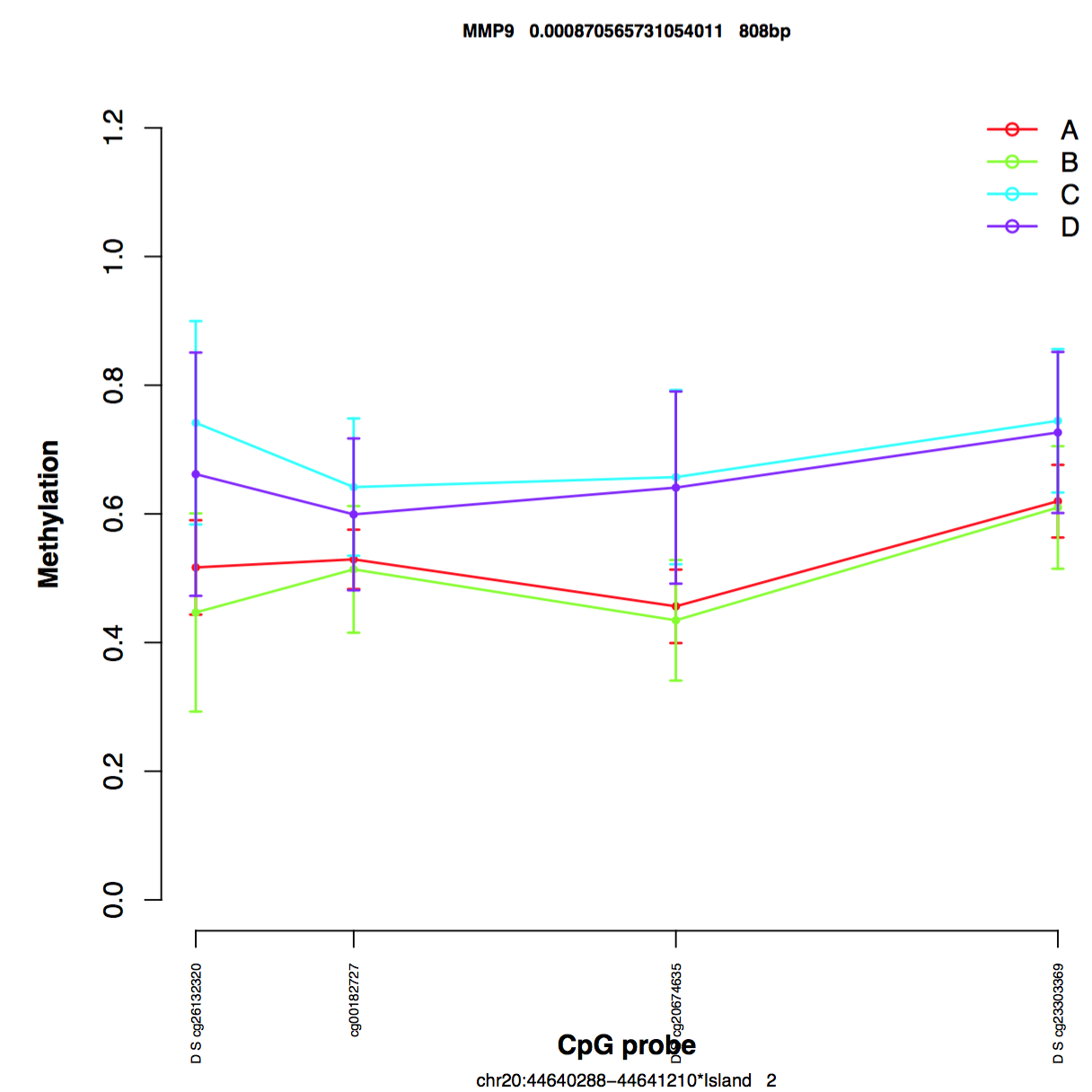


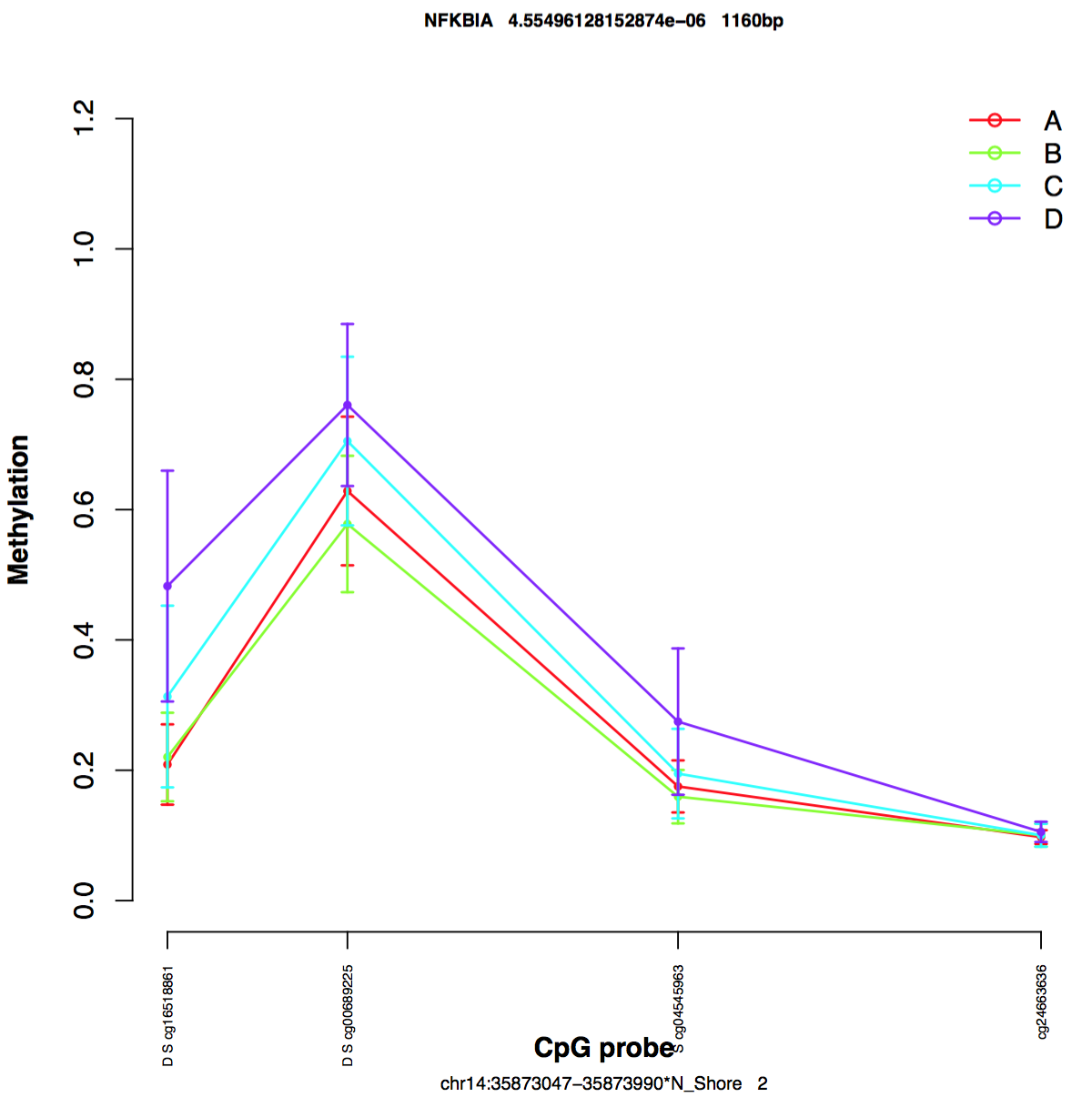

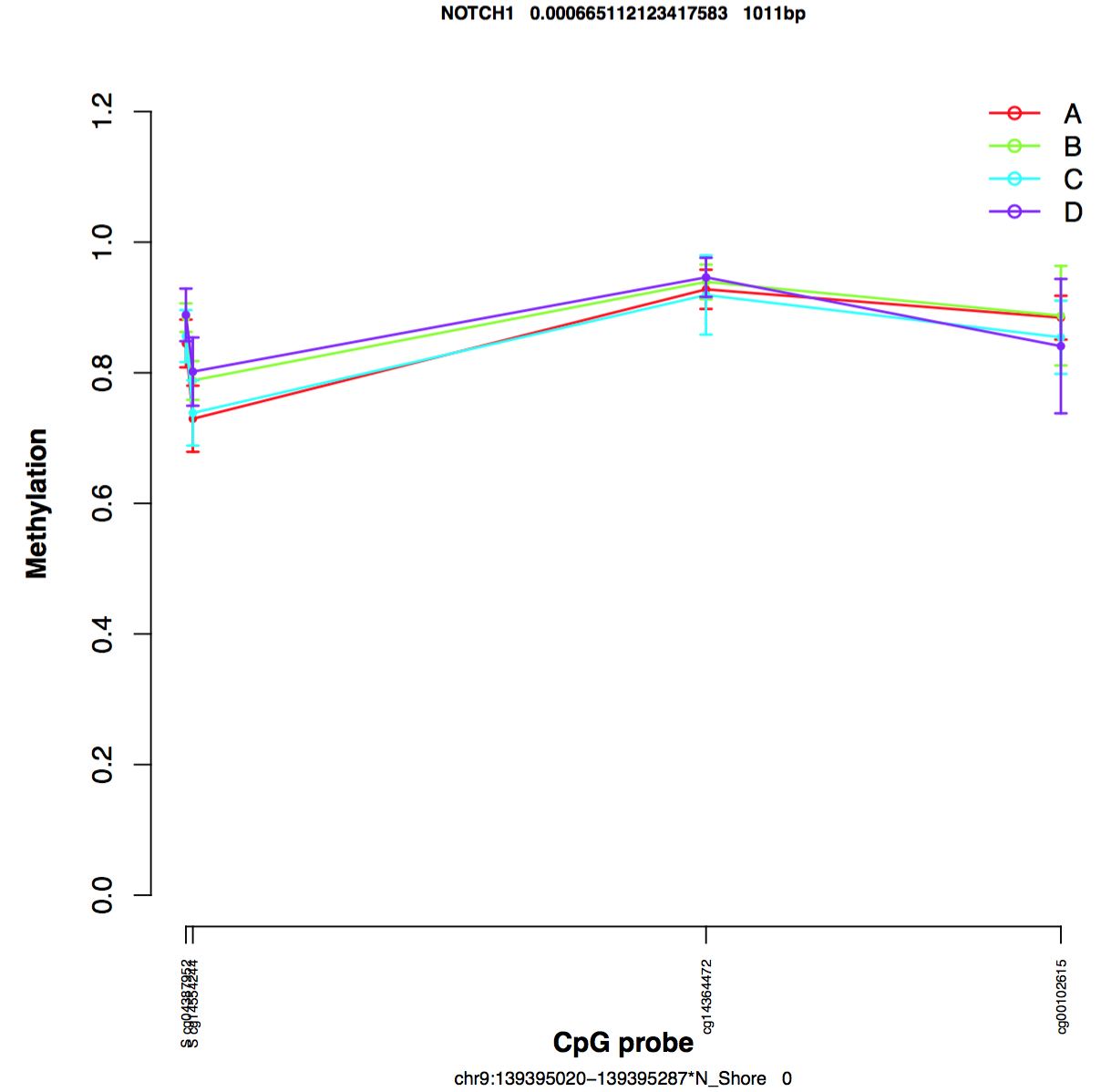

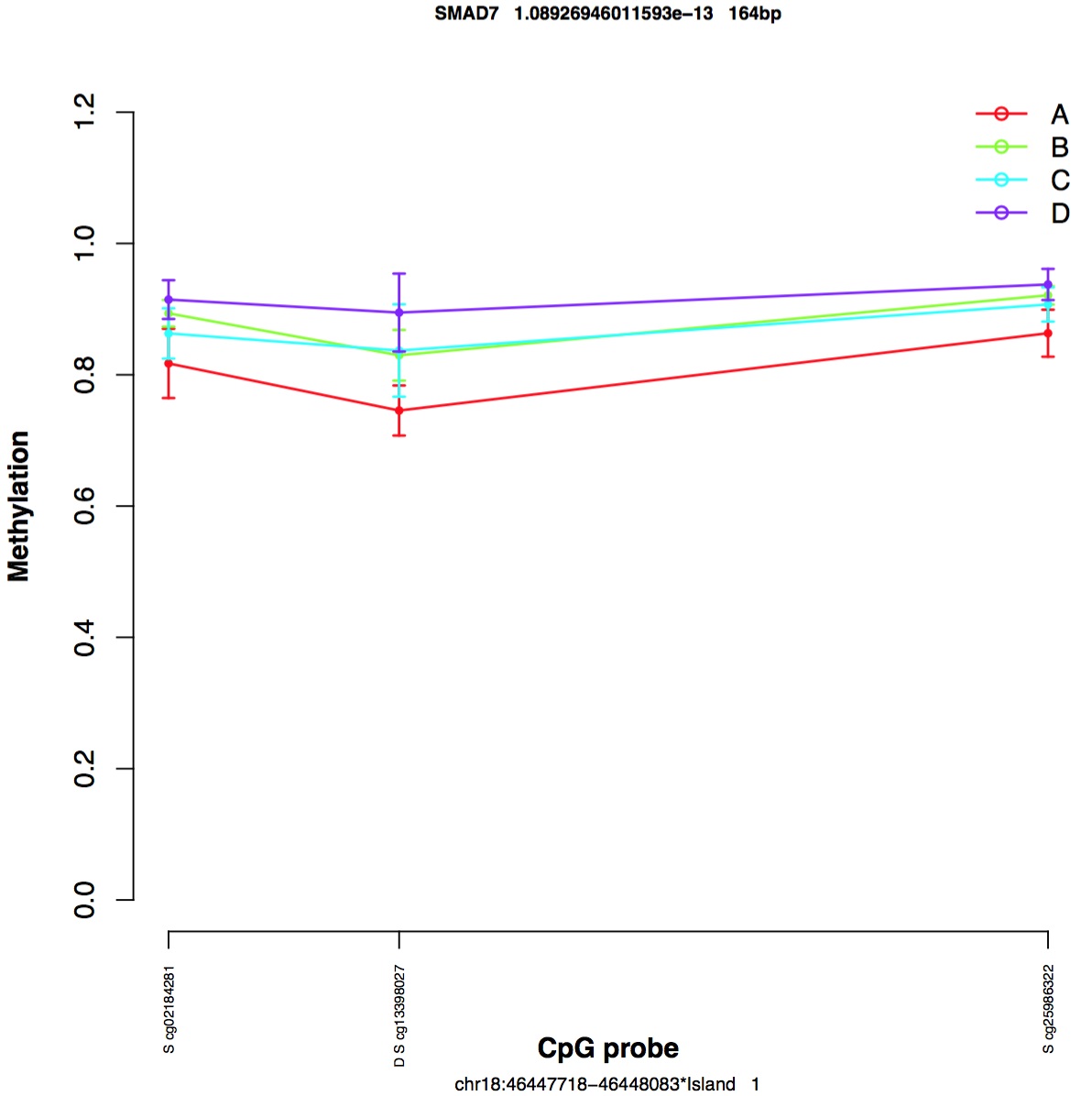

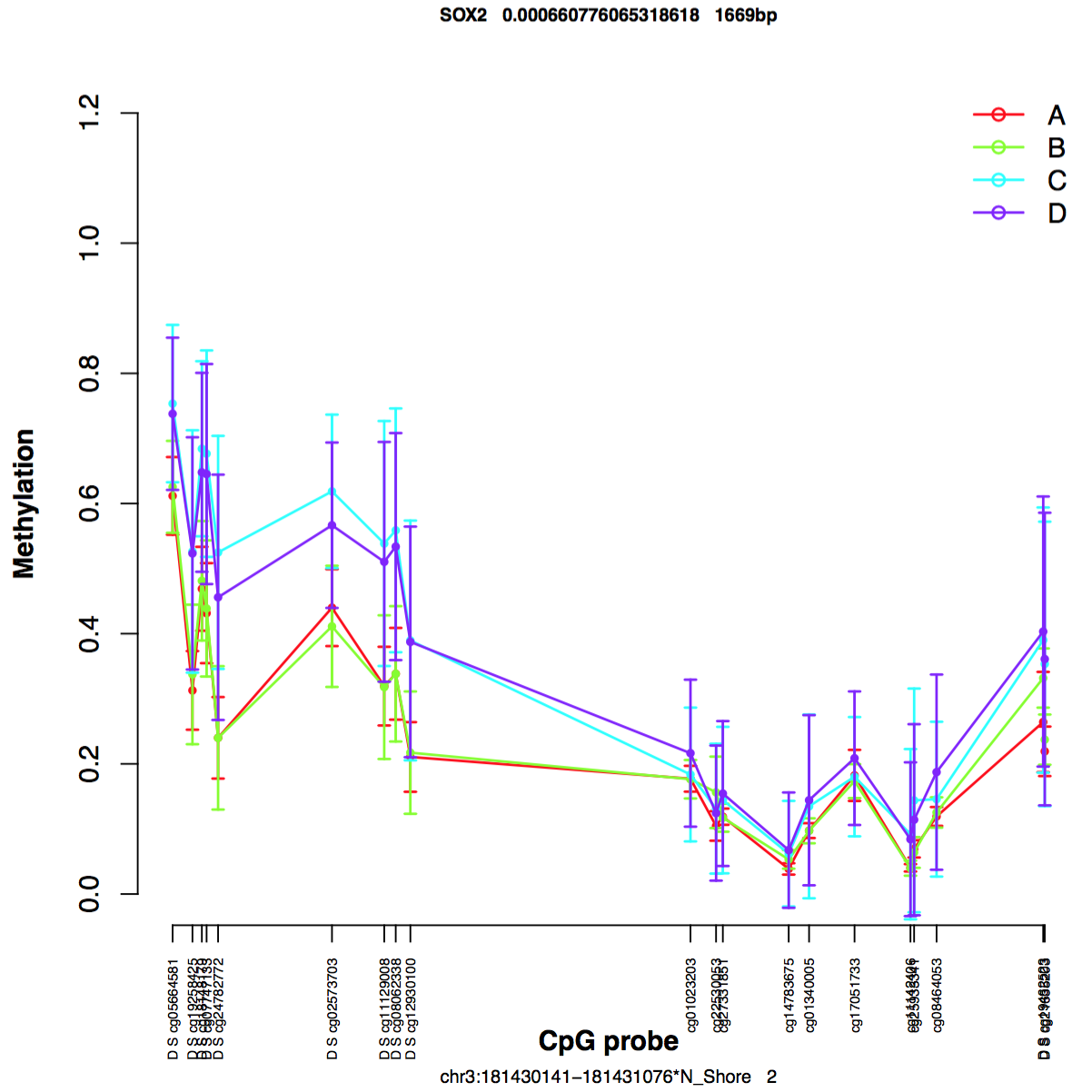

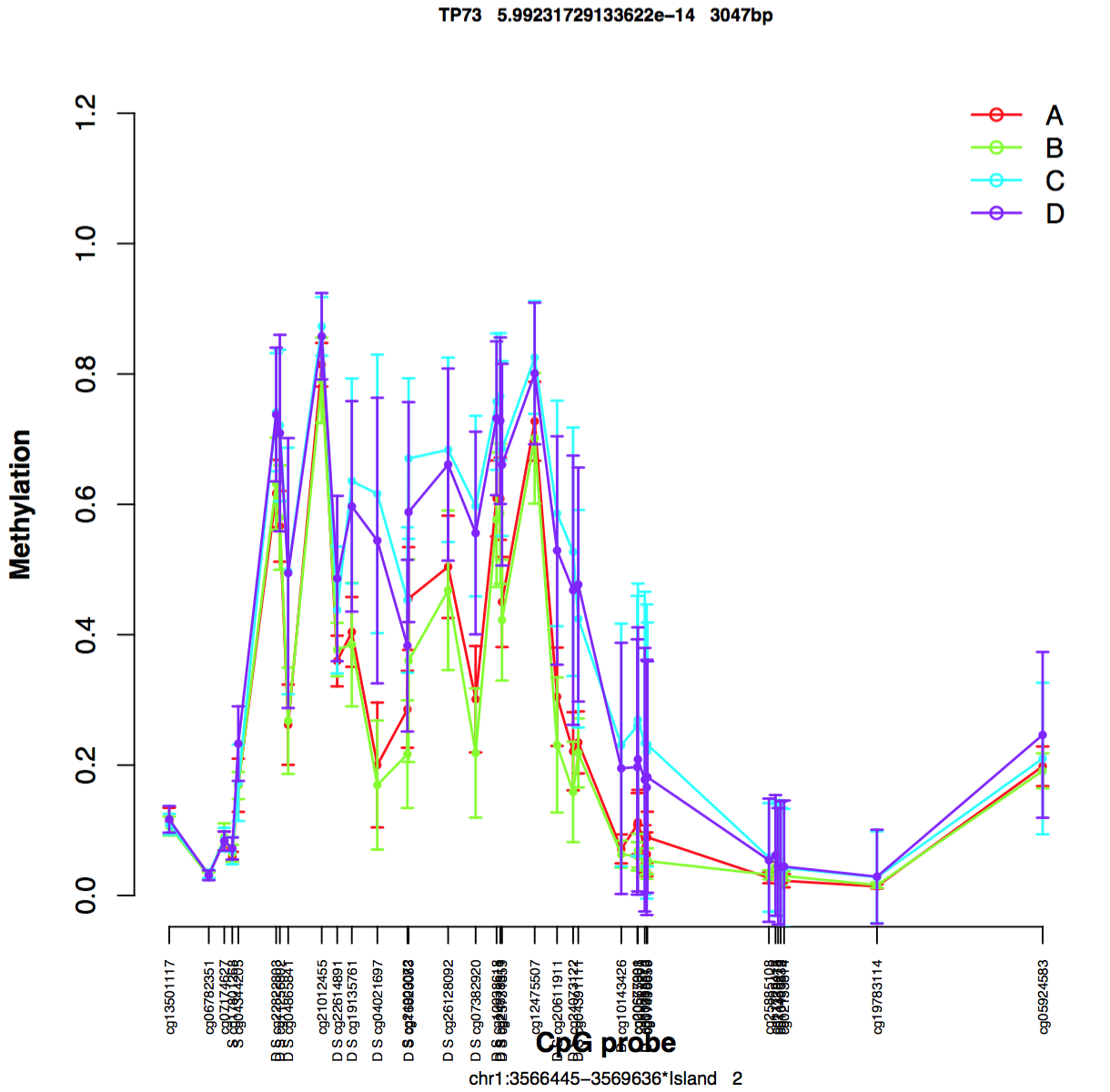

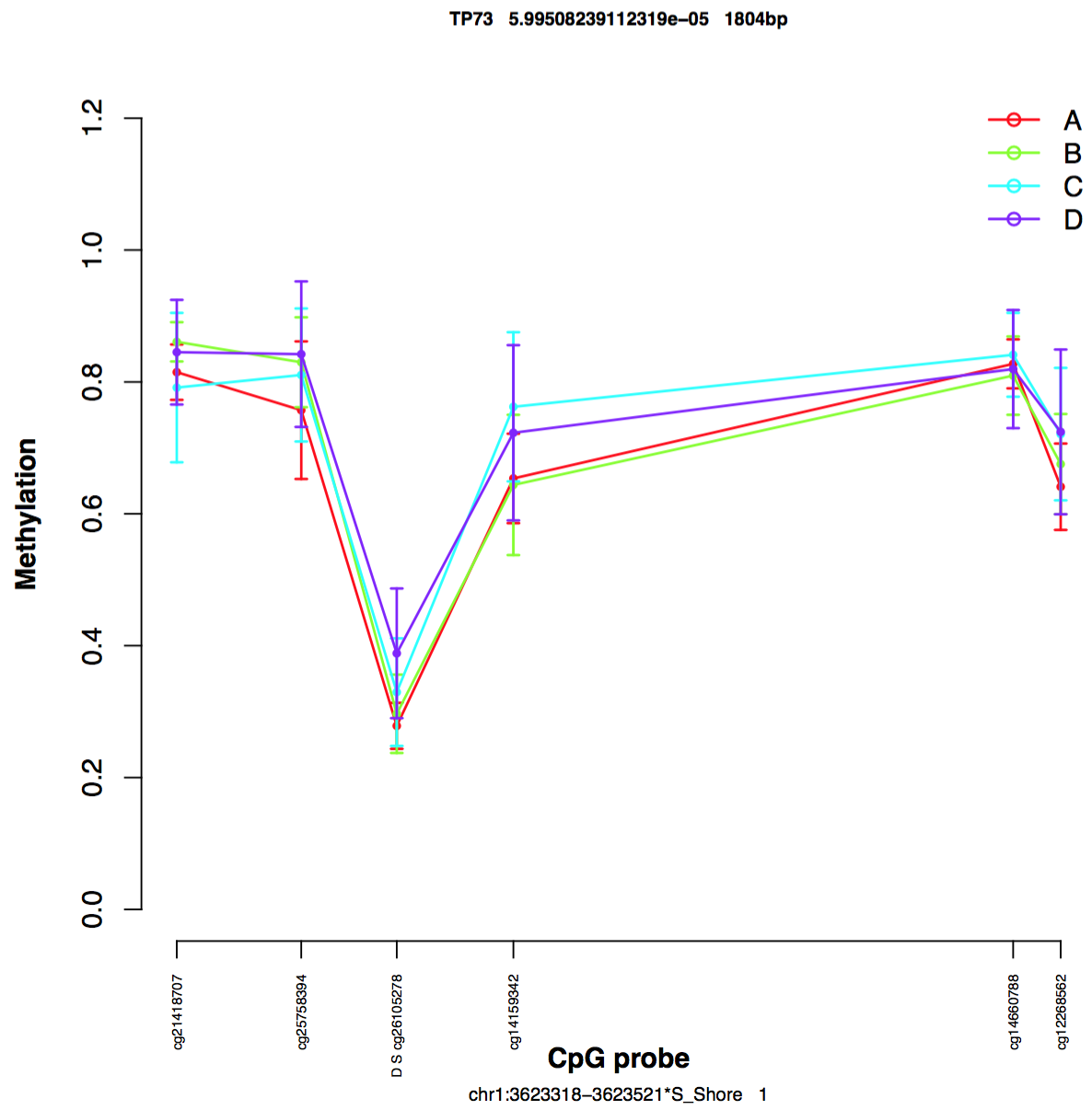

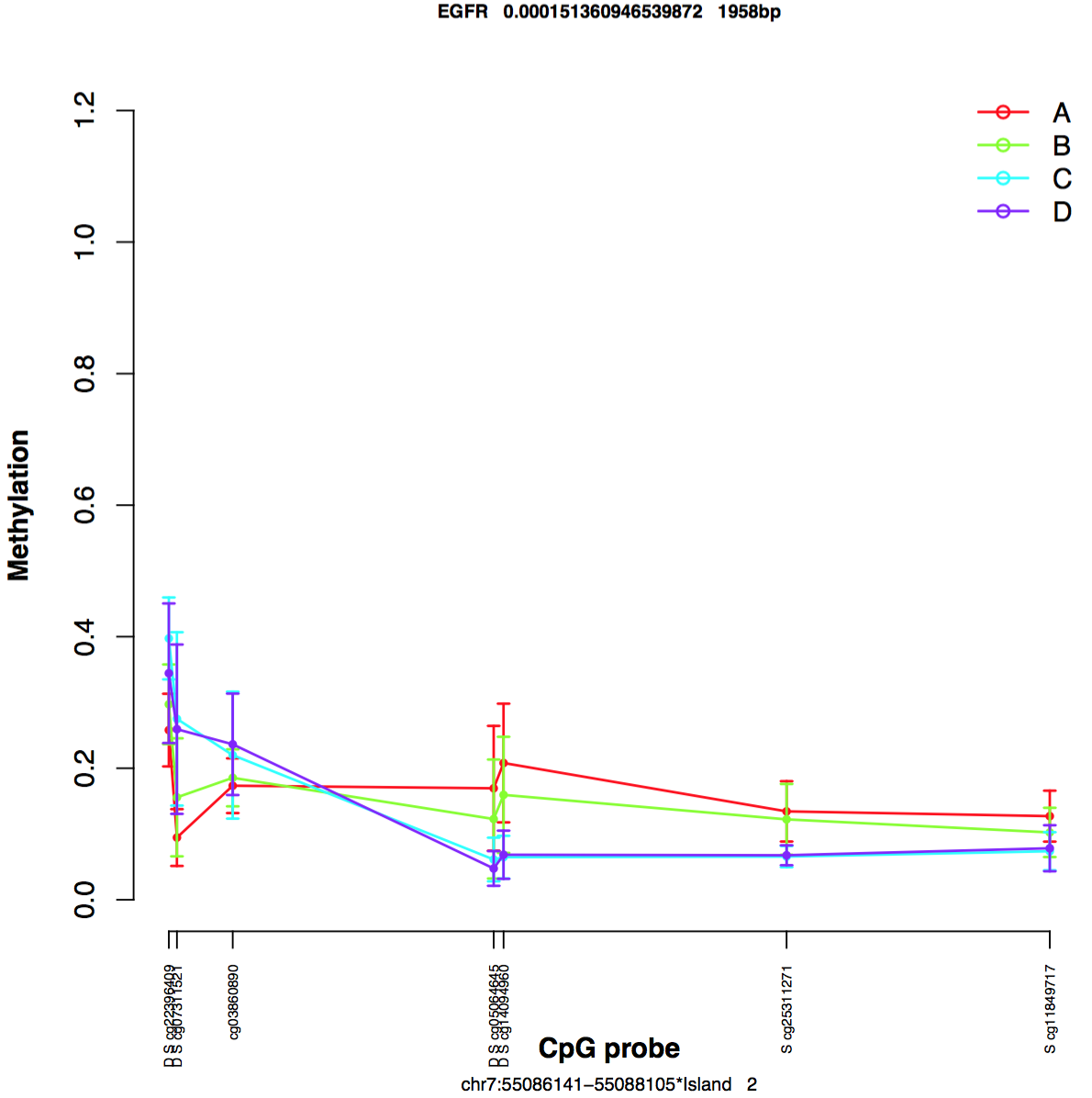

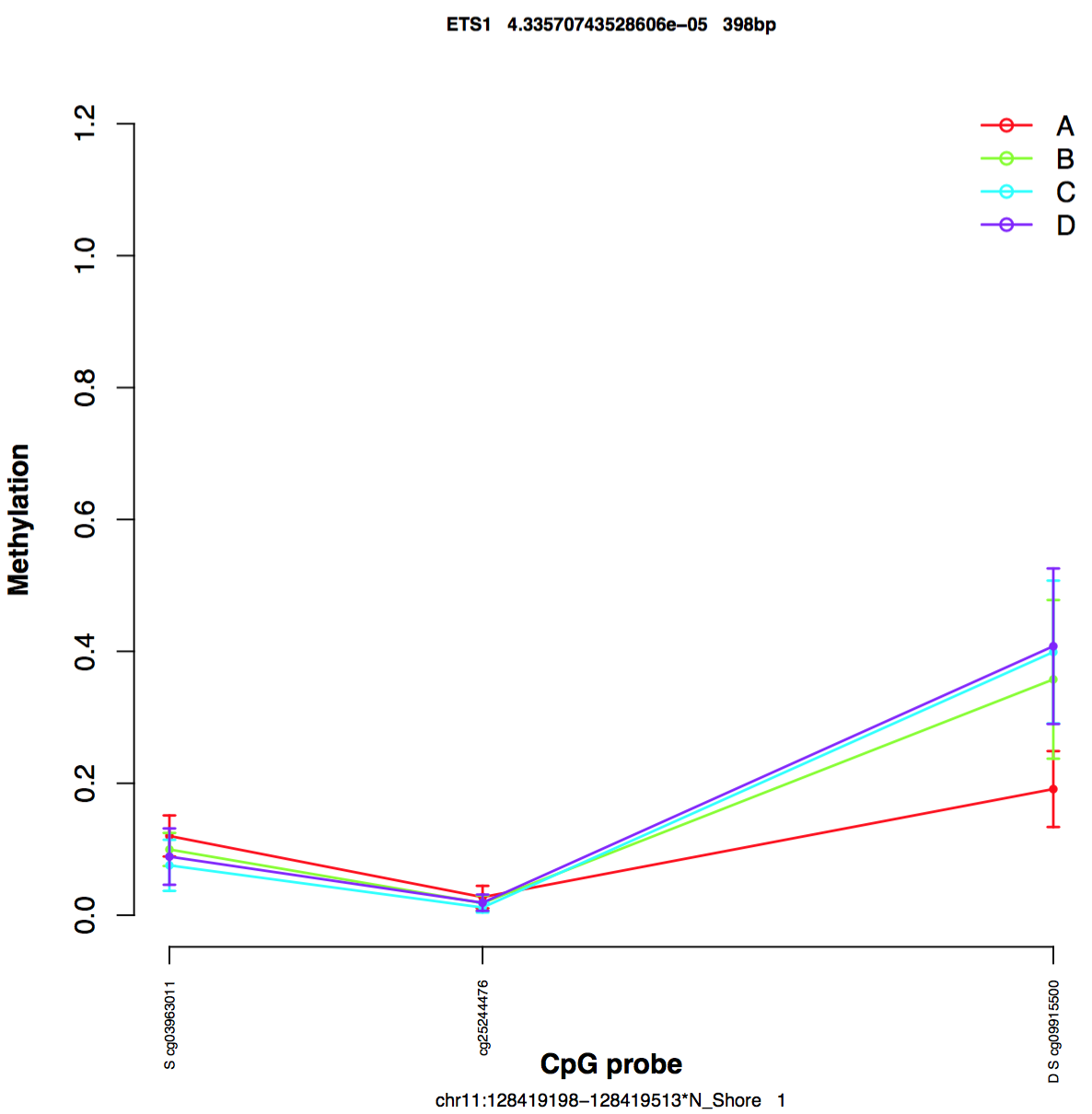

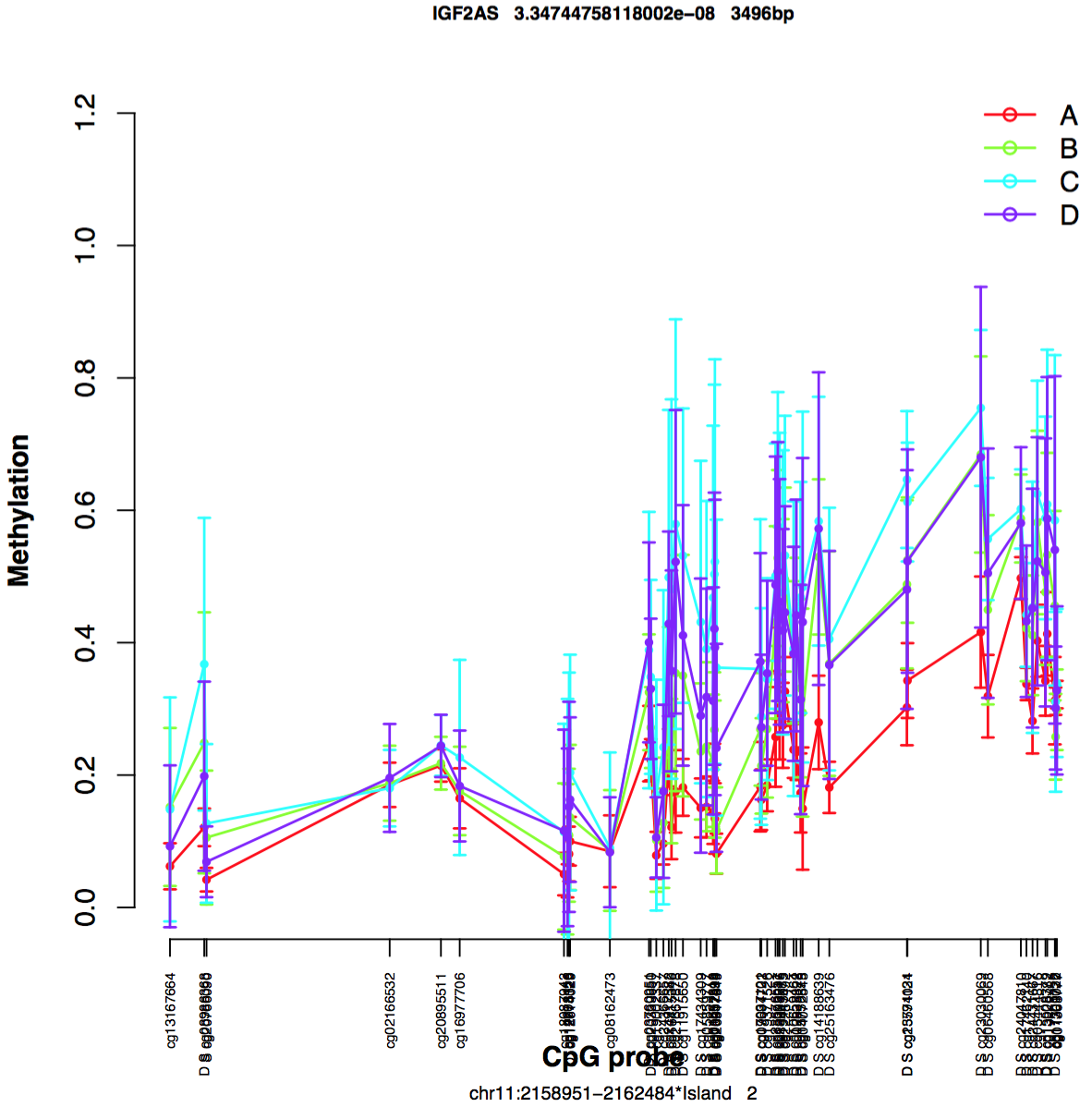

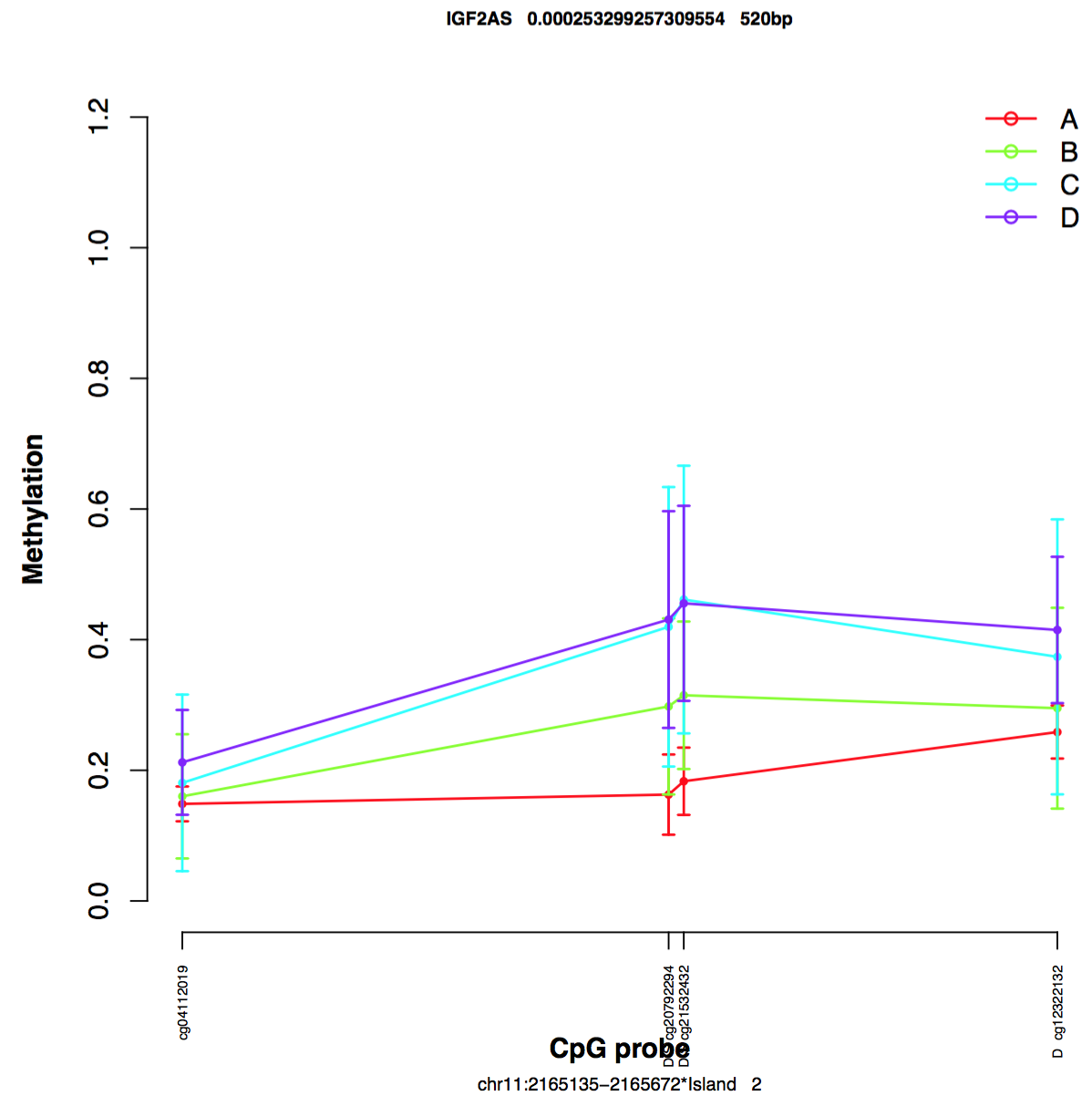

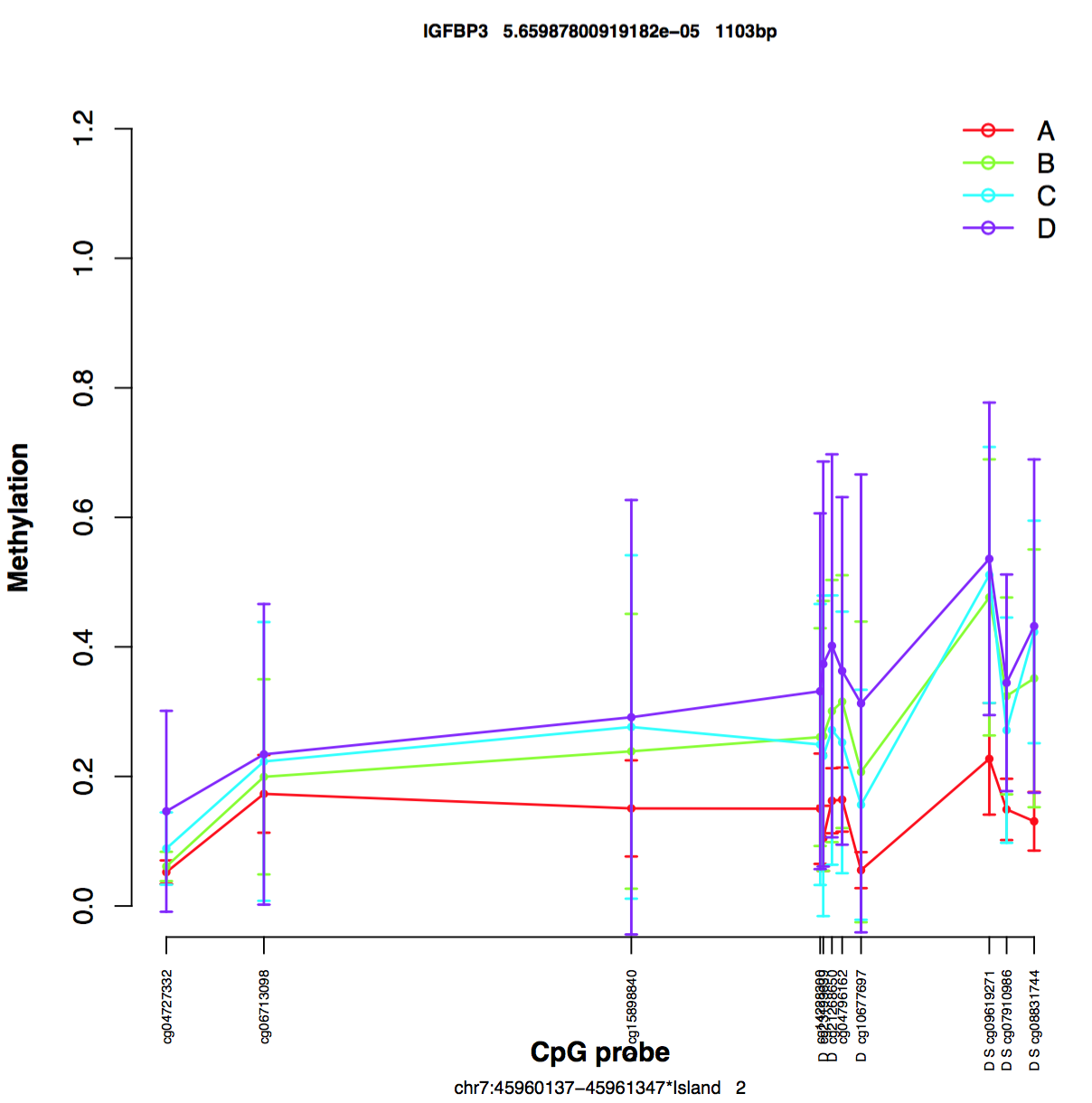

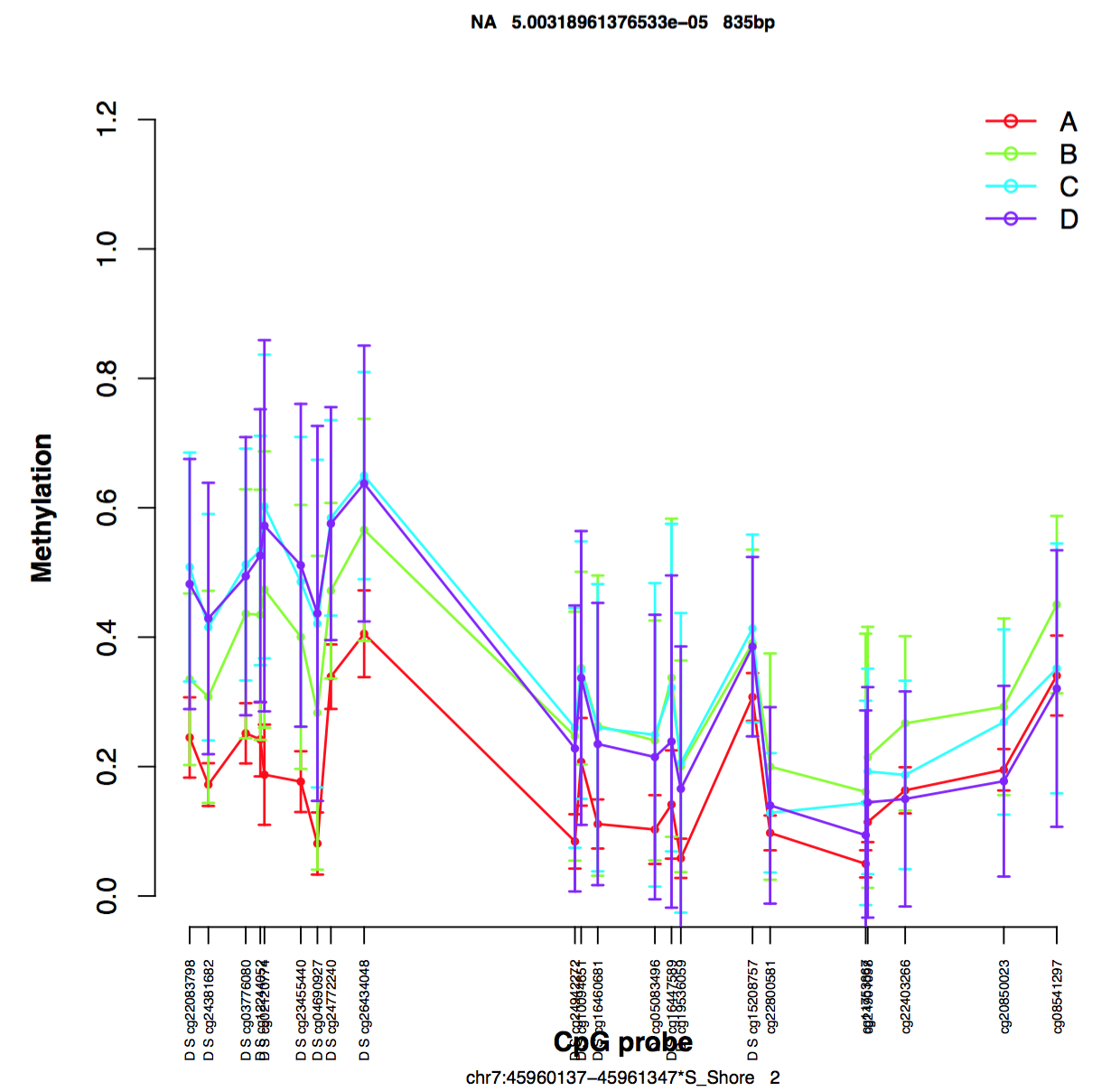

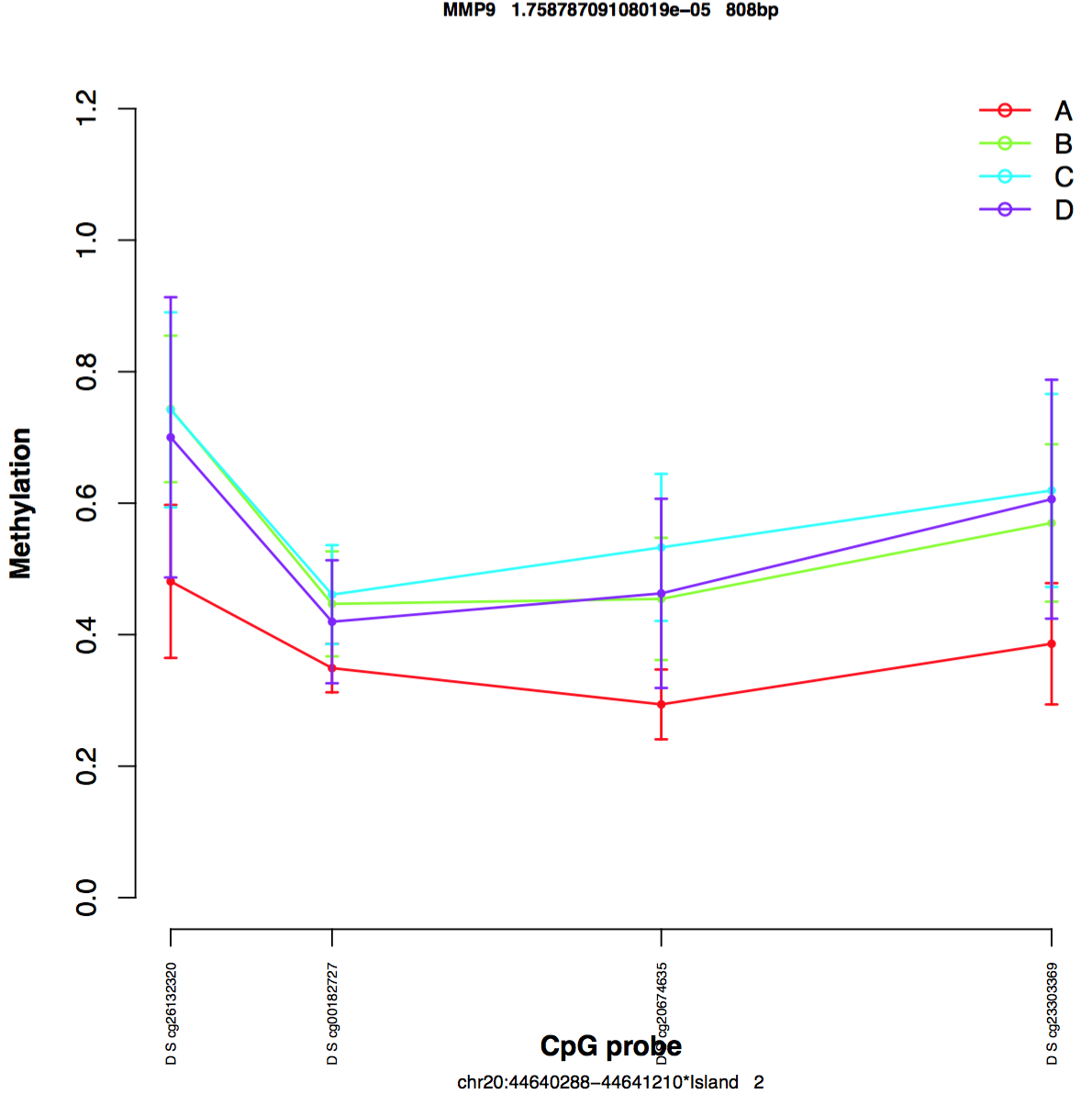

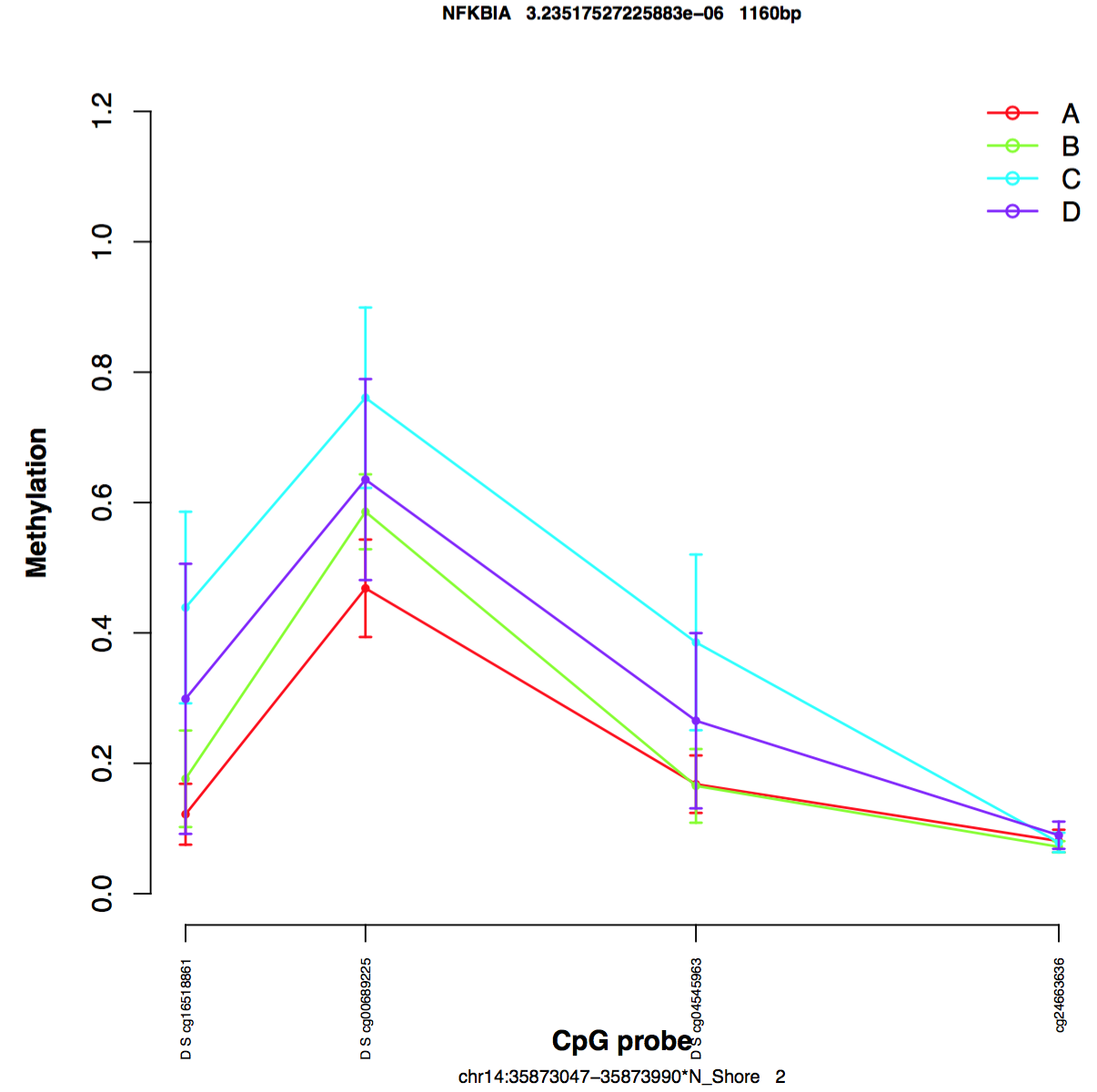

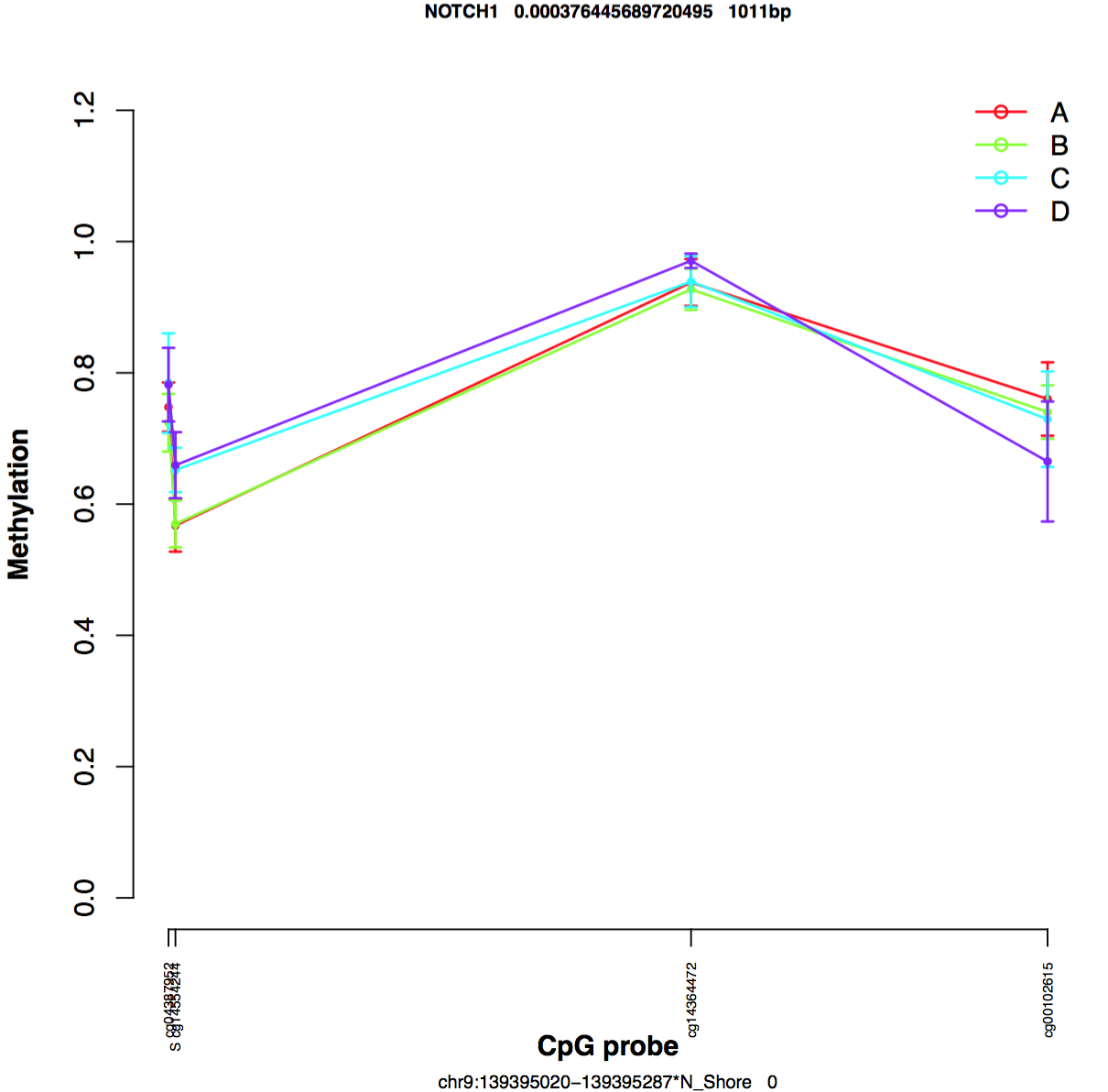

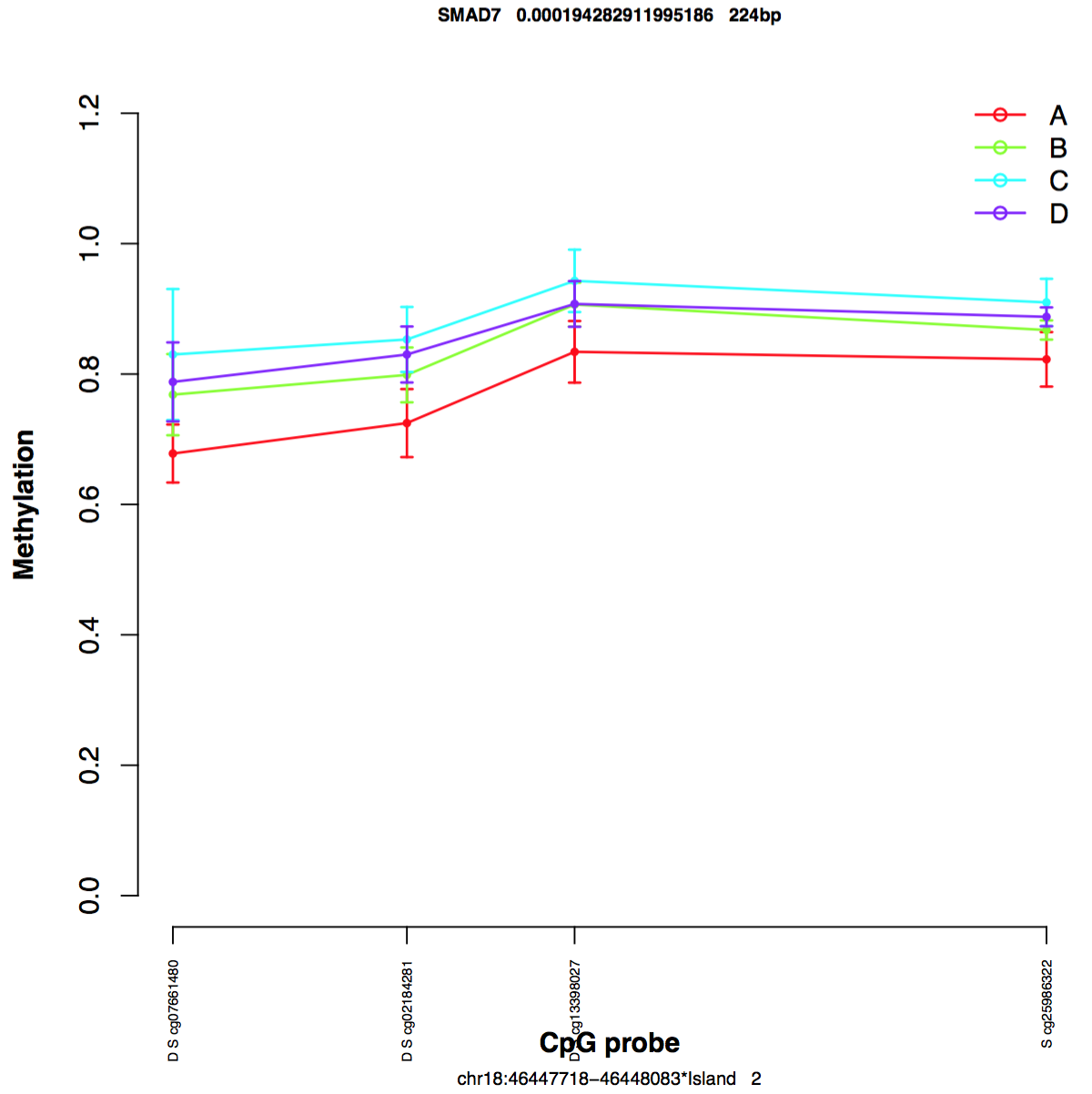

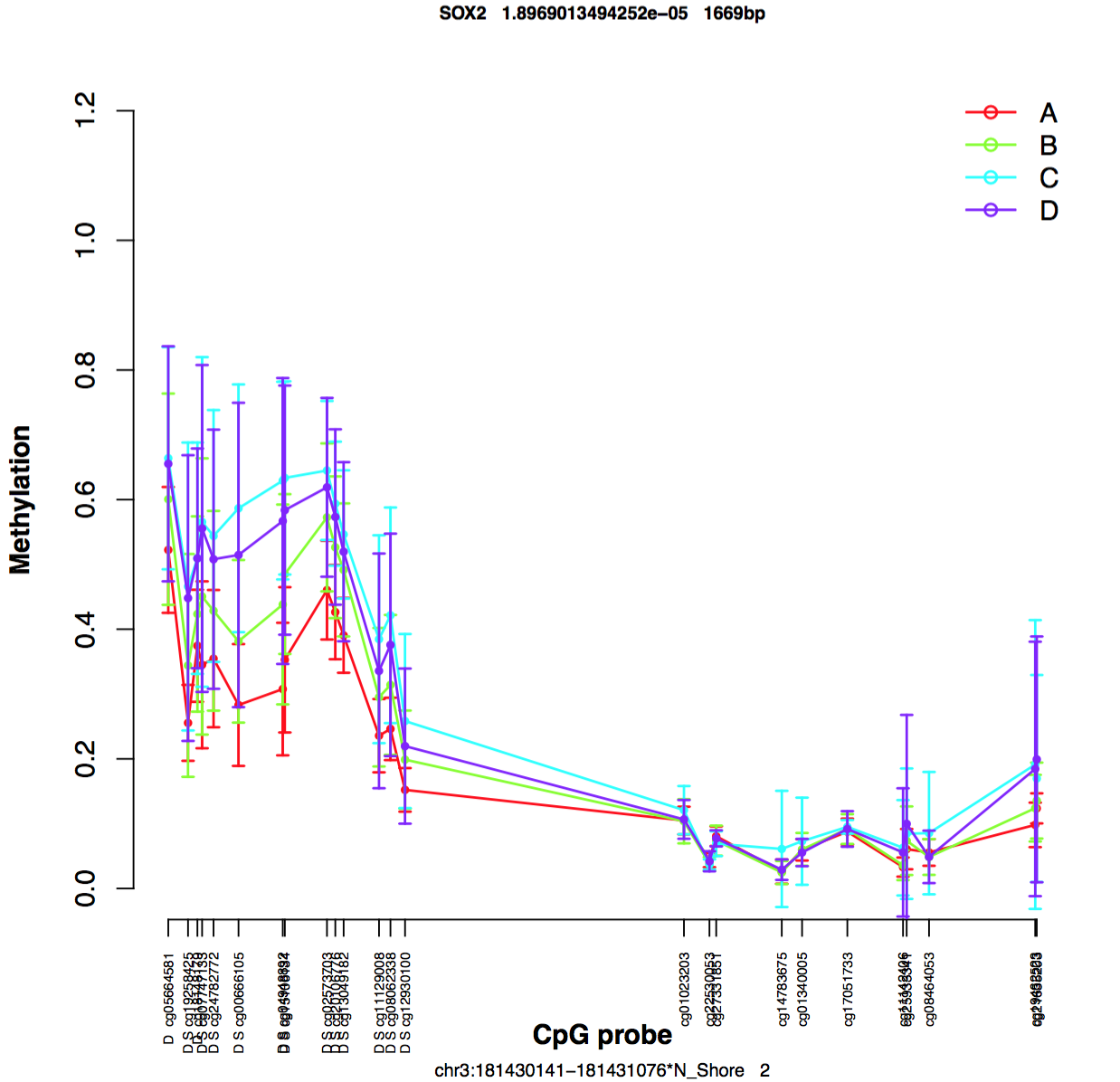

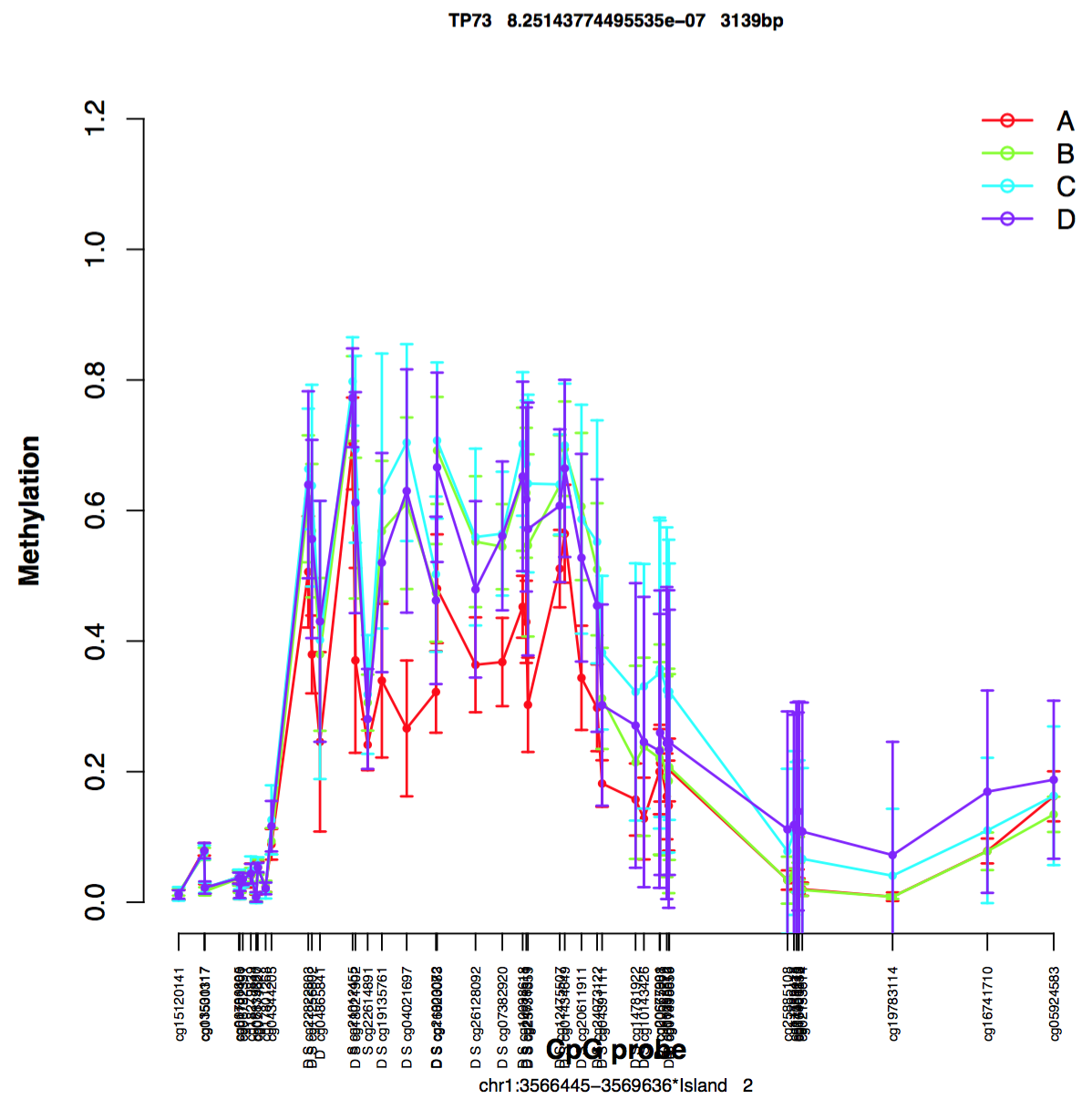

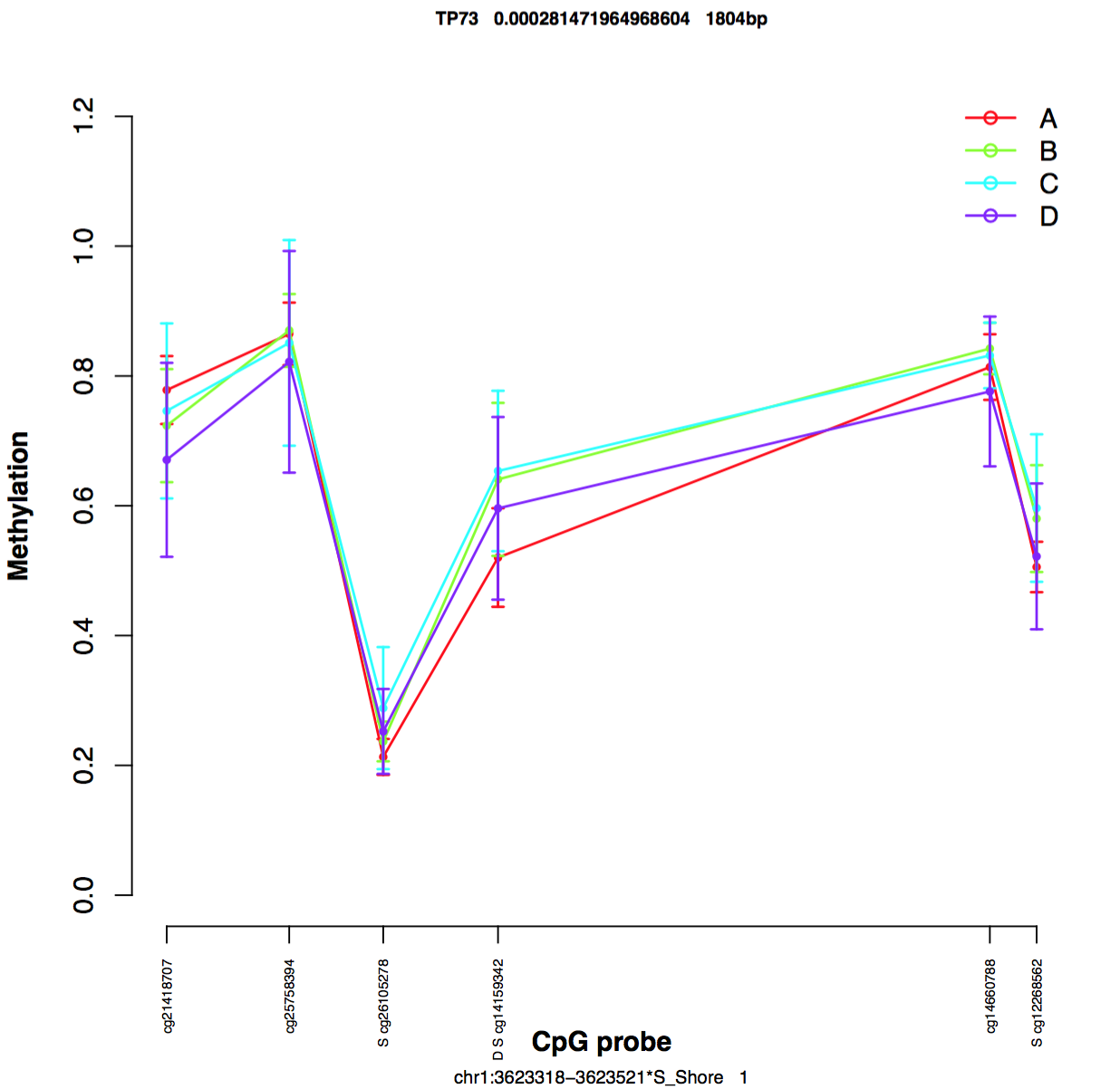

Supplement: Supplementary file 7 [file oncotarget-08-12820-s007.docx]
