# Supplementary material for: Aberrant methylation patterns in colorectal cancer: a meta-analysis
Source: Oncotarget. 2017 Jan 10;8(8):12820–30. doi: 10.18632/oncotarget.14590 (PMC5355058; doi:10.18632/oncotarget.14590)

**Supplementary File 8** – Scatter plots Age versus CpGs methylation levels; plus linear regression from TCGA dataset. Plots showing the DNA methylation value of the most significant CpG site within each BOP associated to the 10 DM hubs resulted from N1xCRC network meta-analysis (Table 3). The dots (for each 12 CpG probes), line an p-value in red color refer to CRC samples and cyan refers to normal samples (adjacent to tumor). We considered the and plotted each methylation value against the age of the subjects.


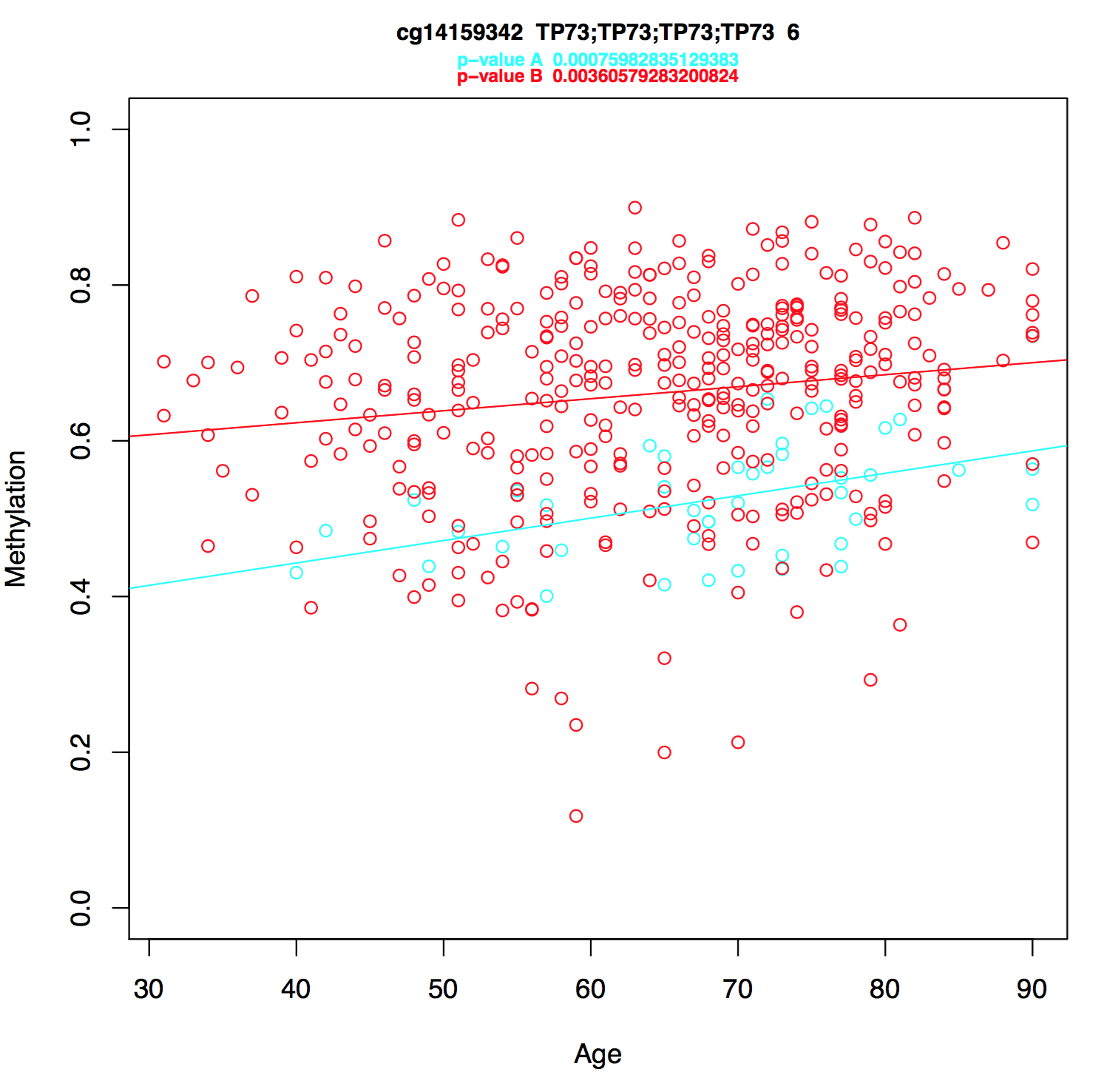

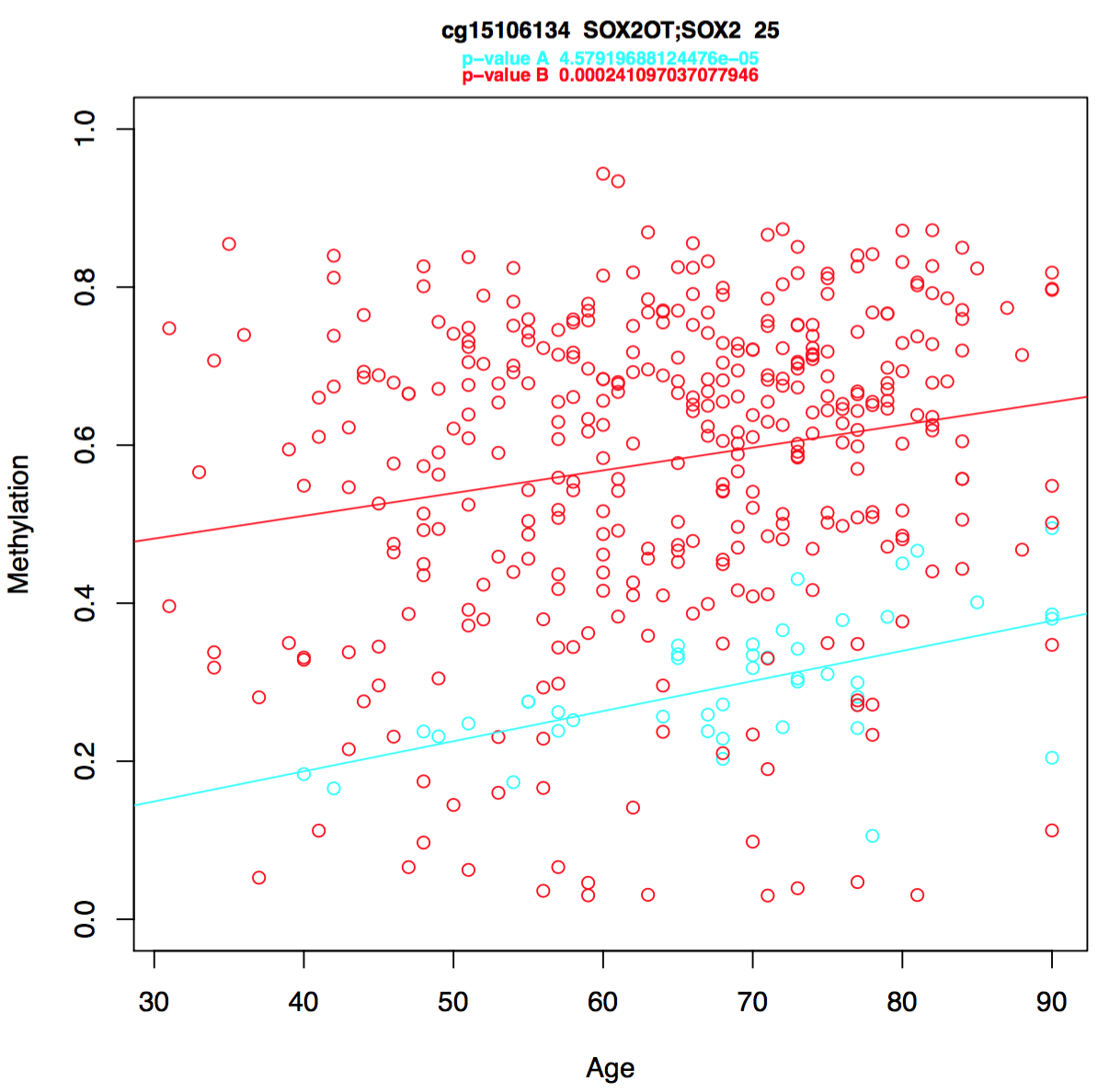

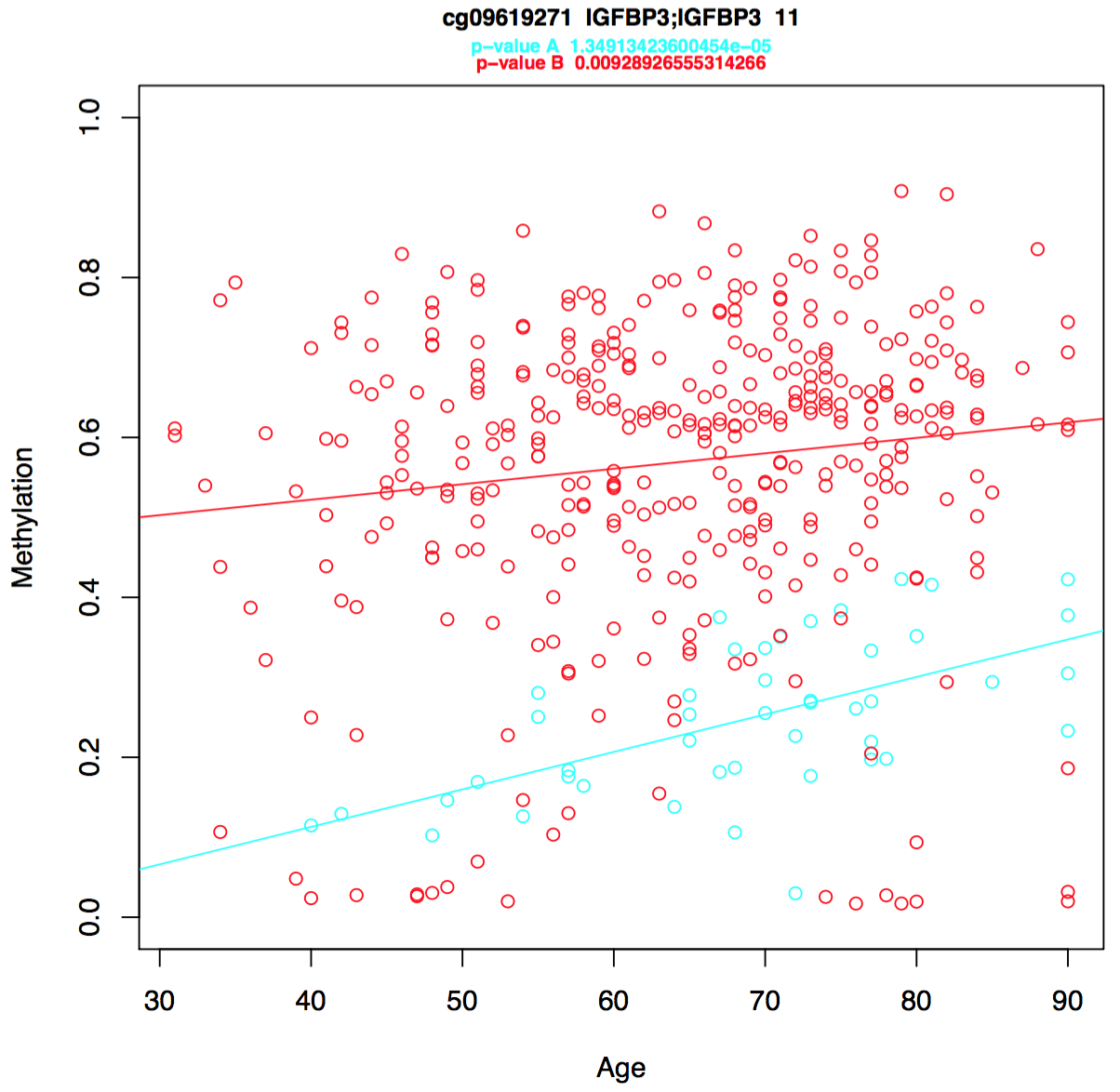

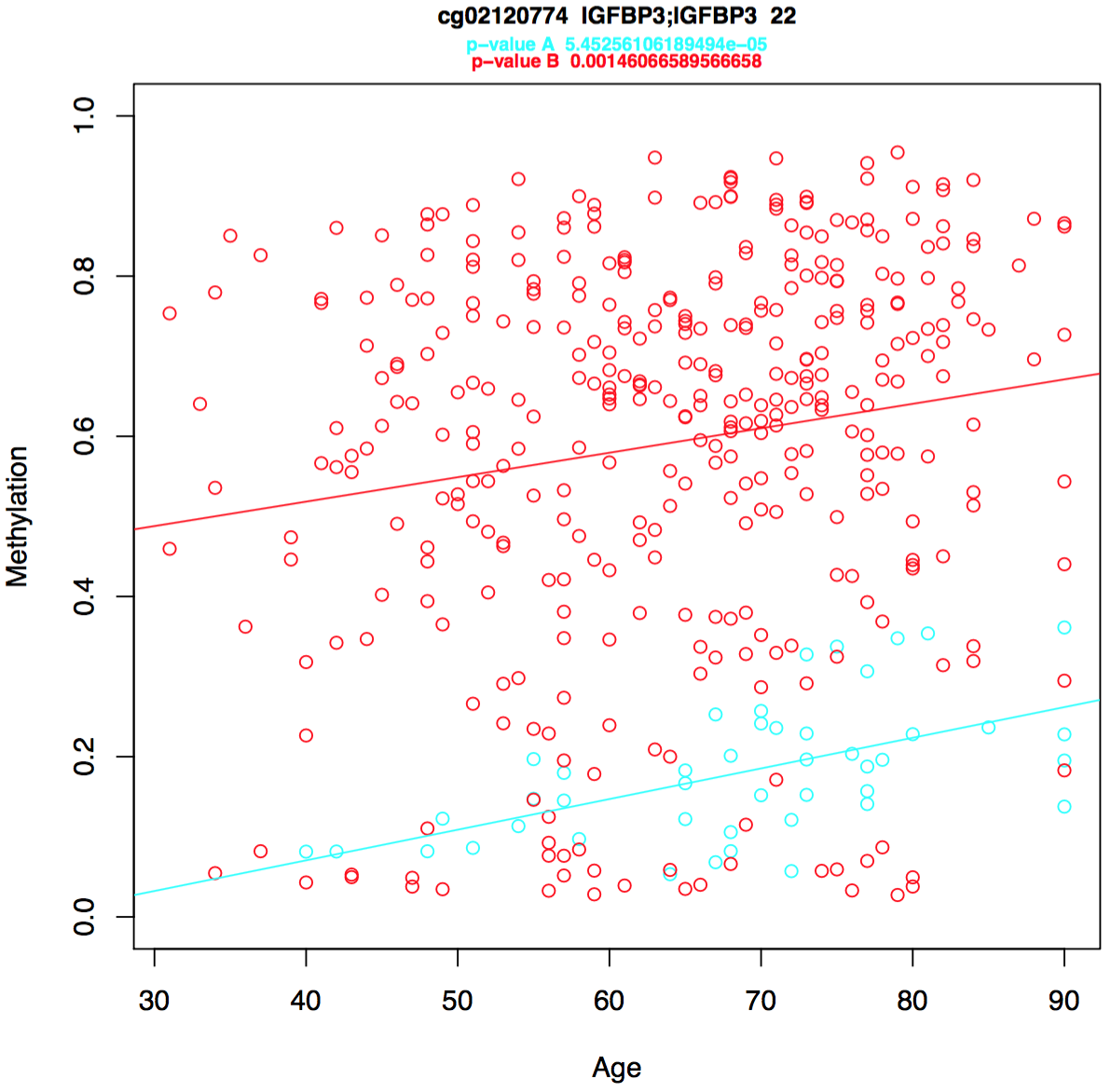

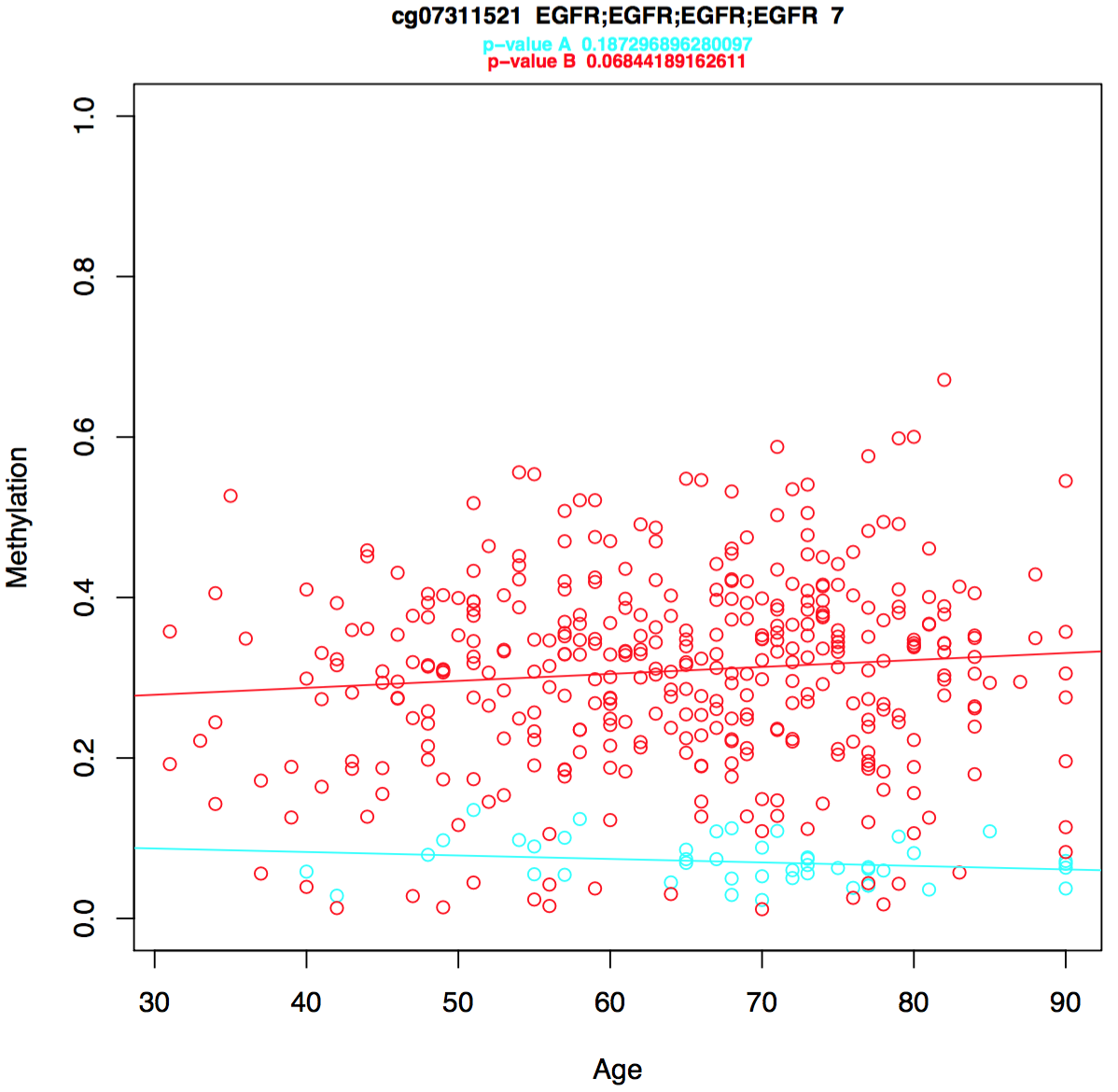

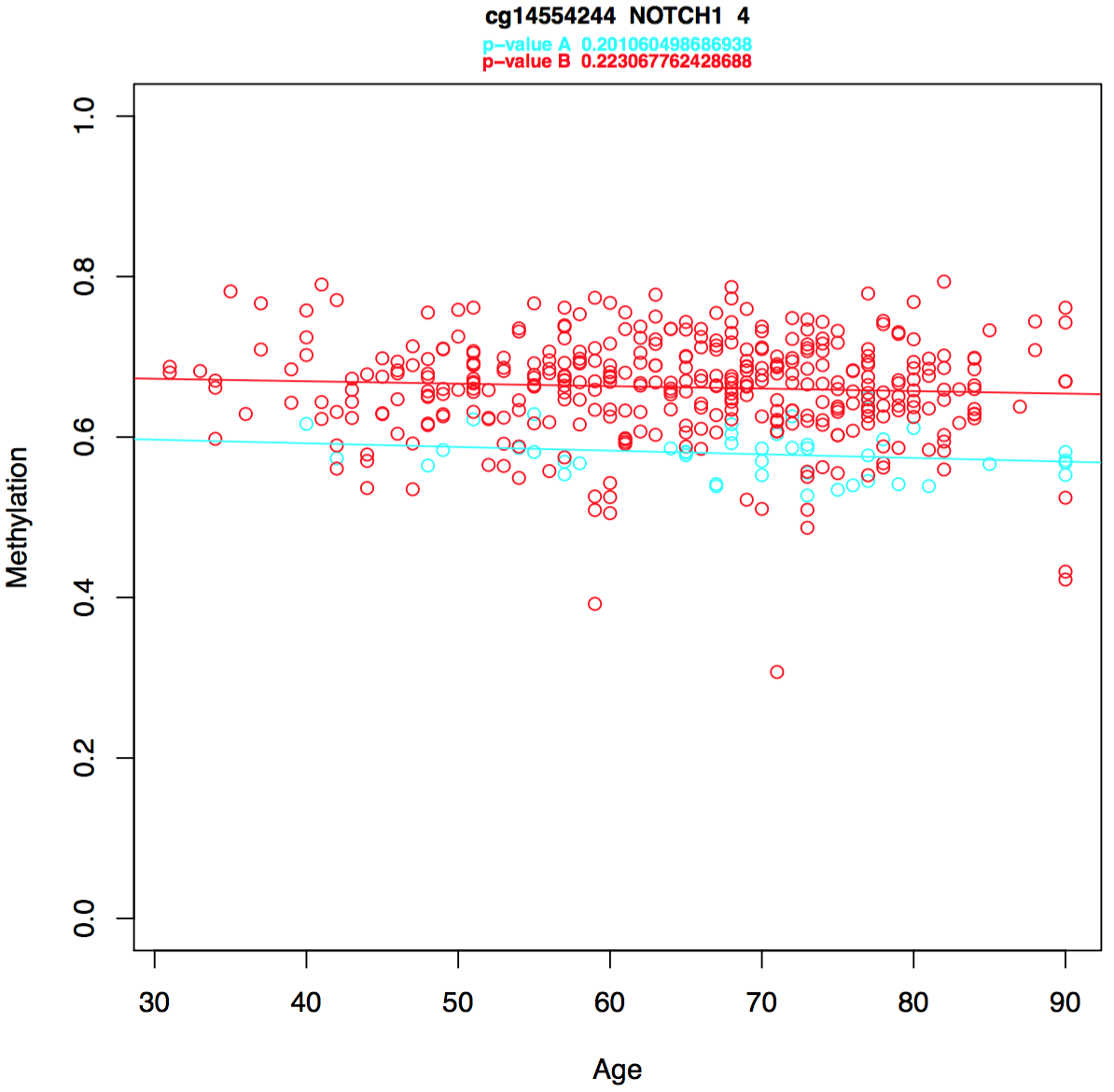

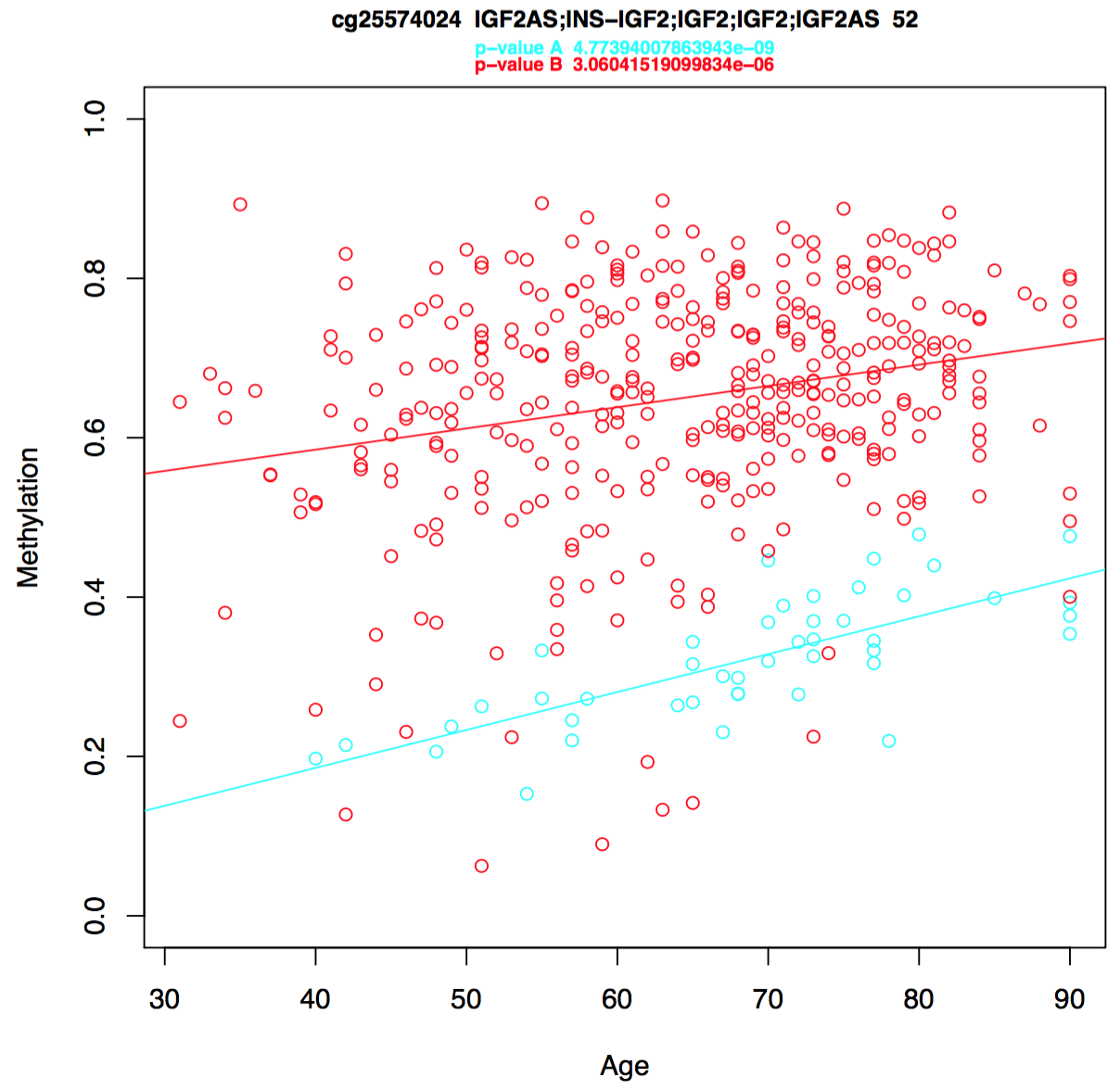

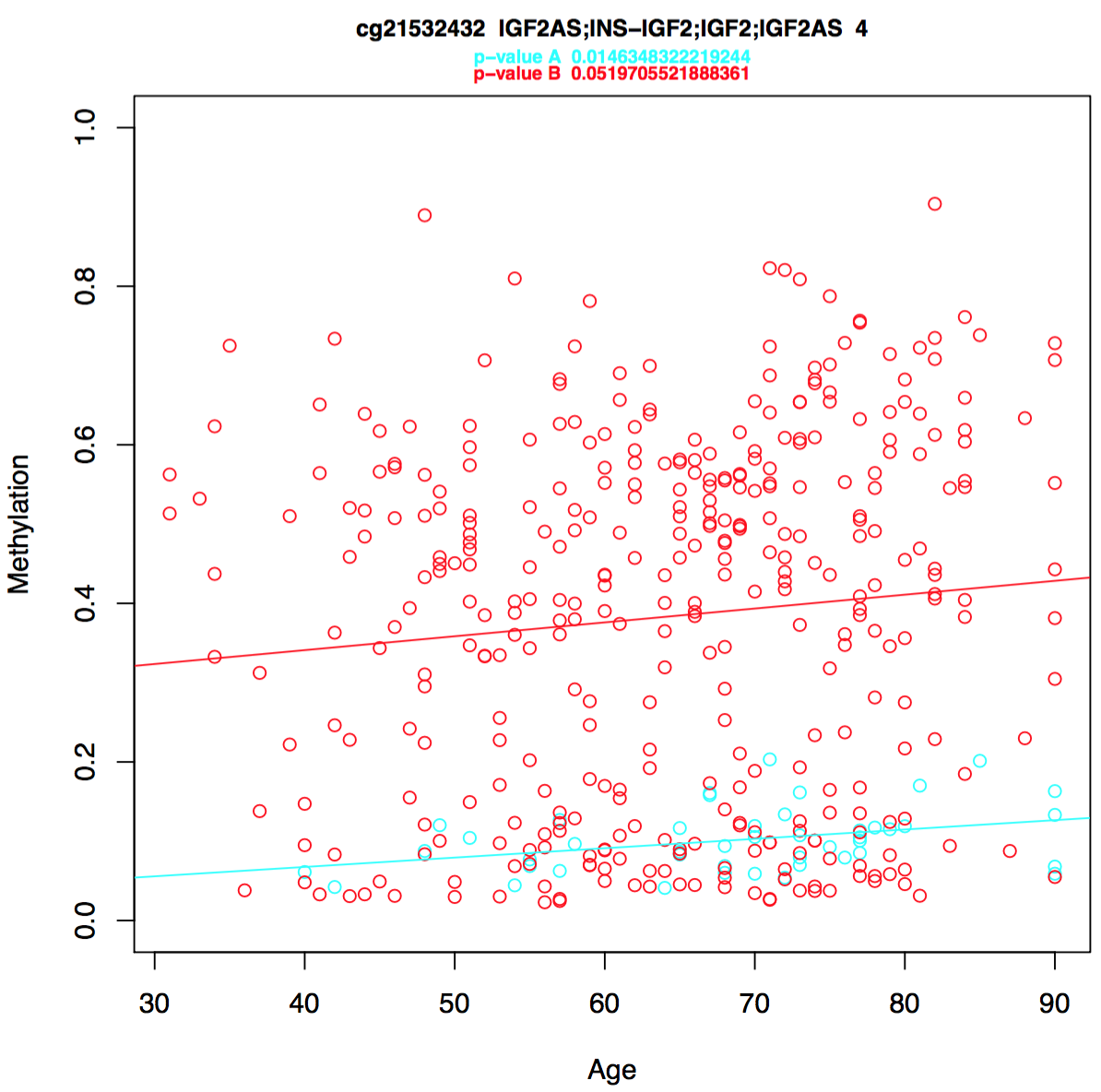

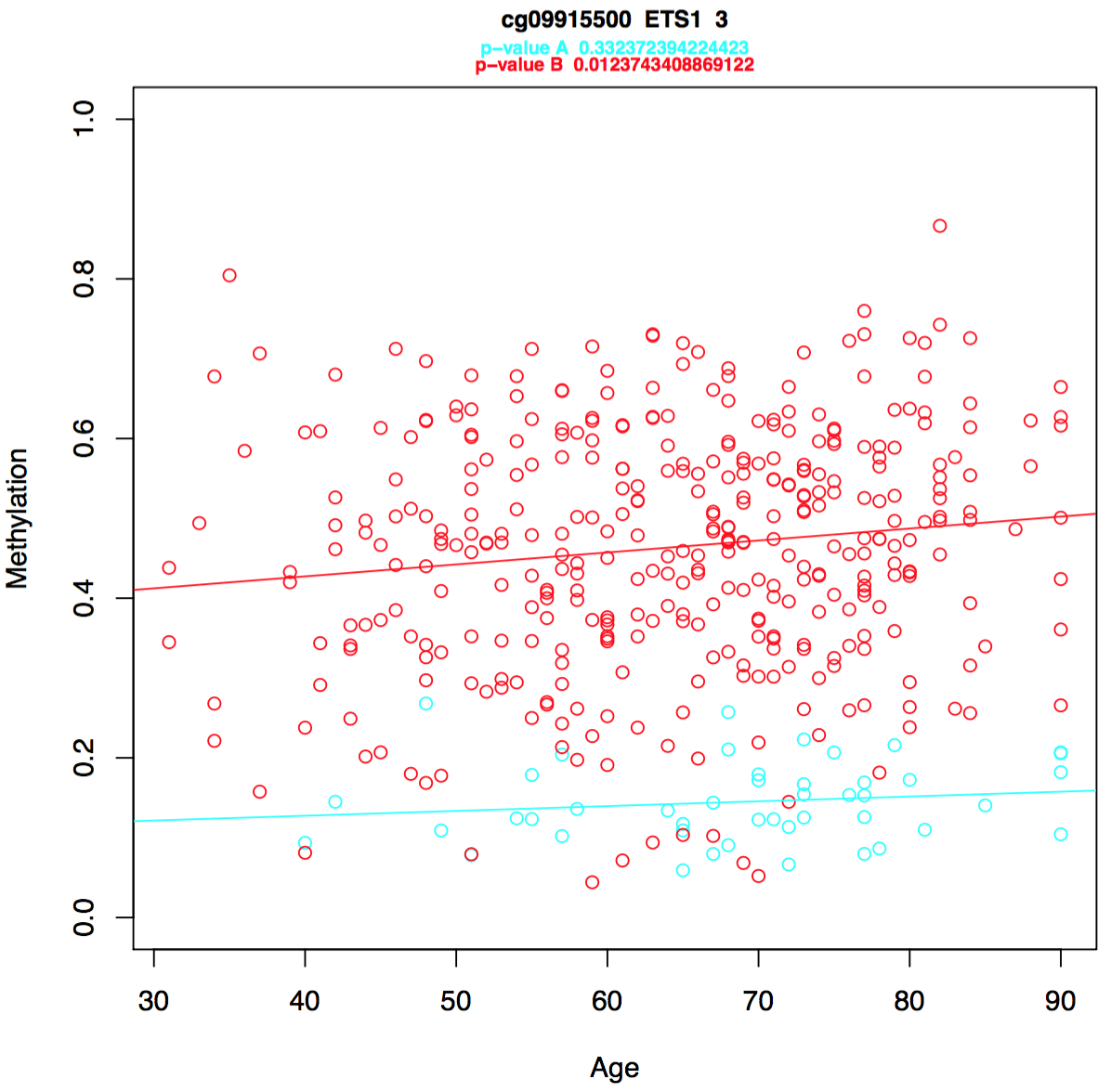

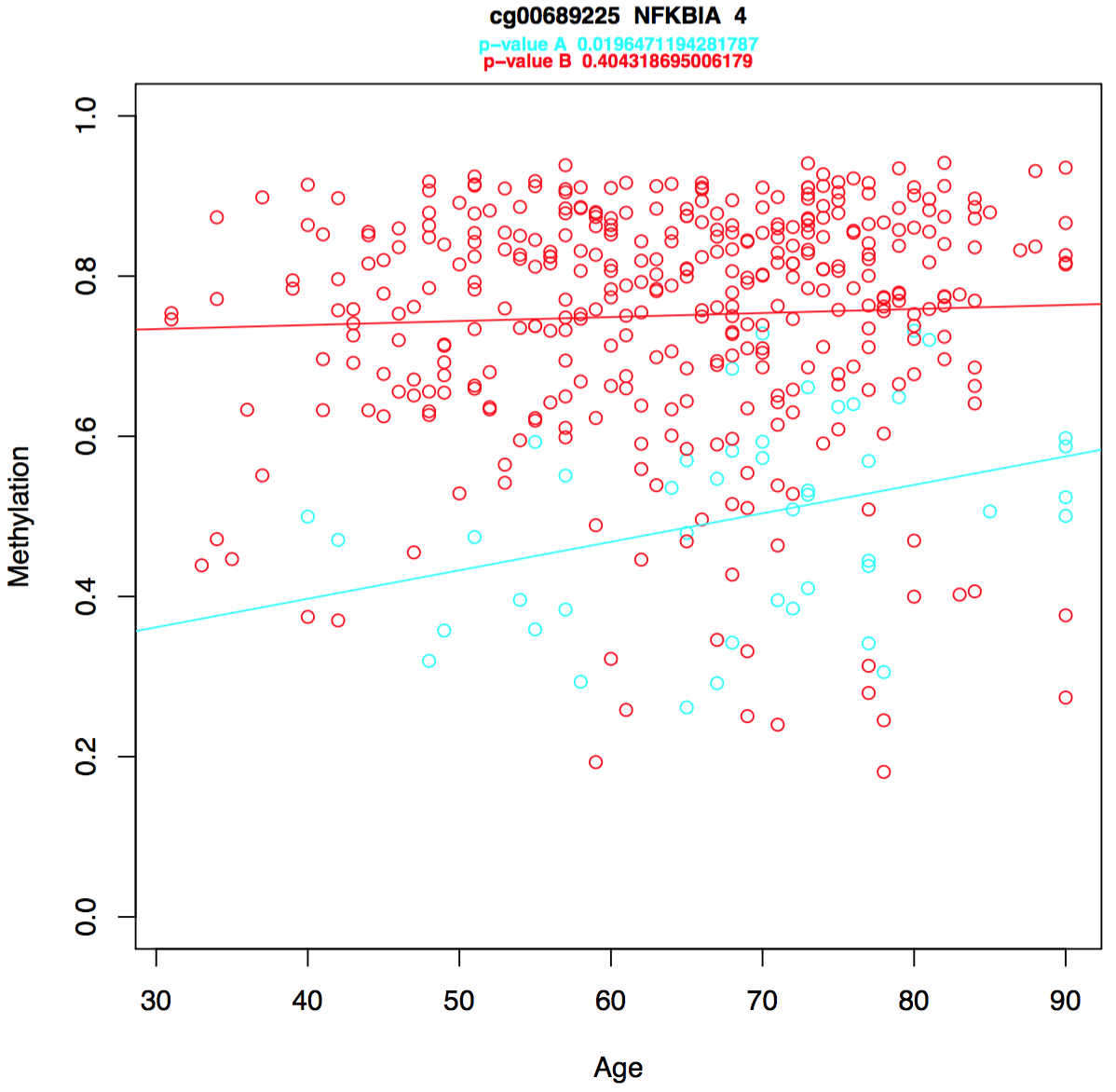

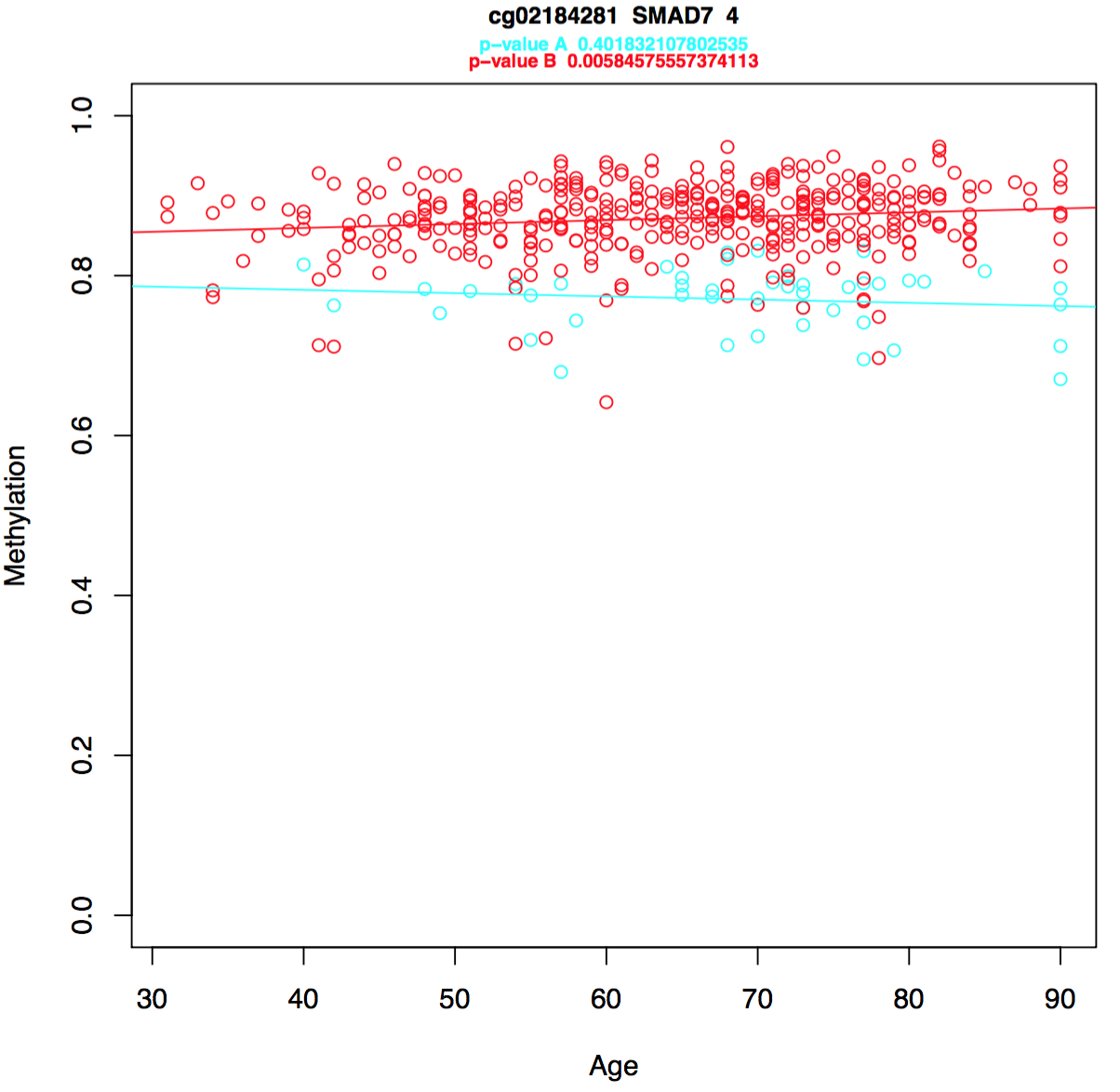

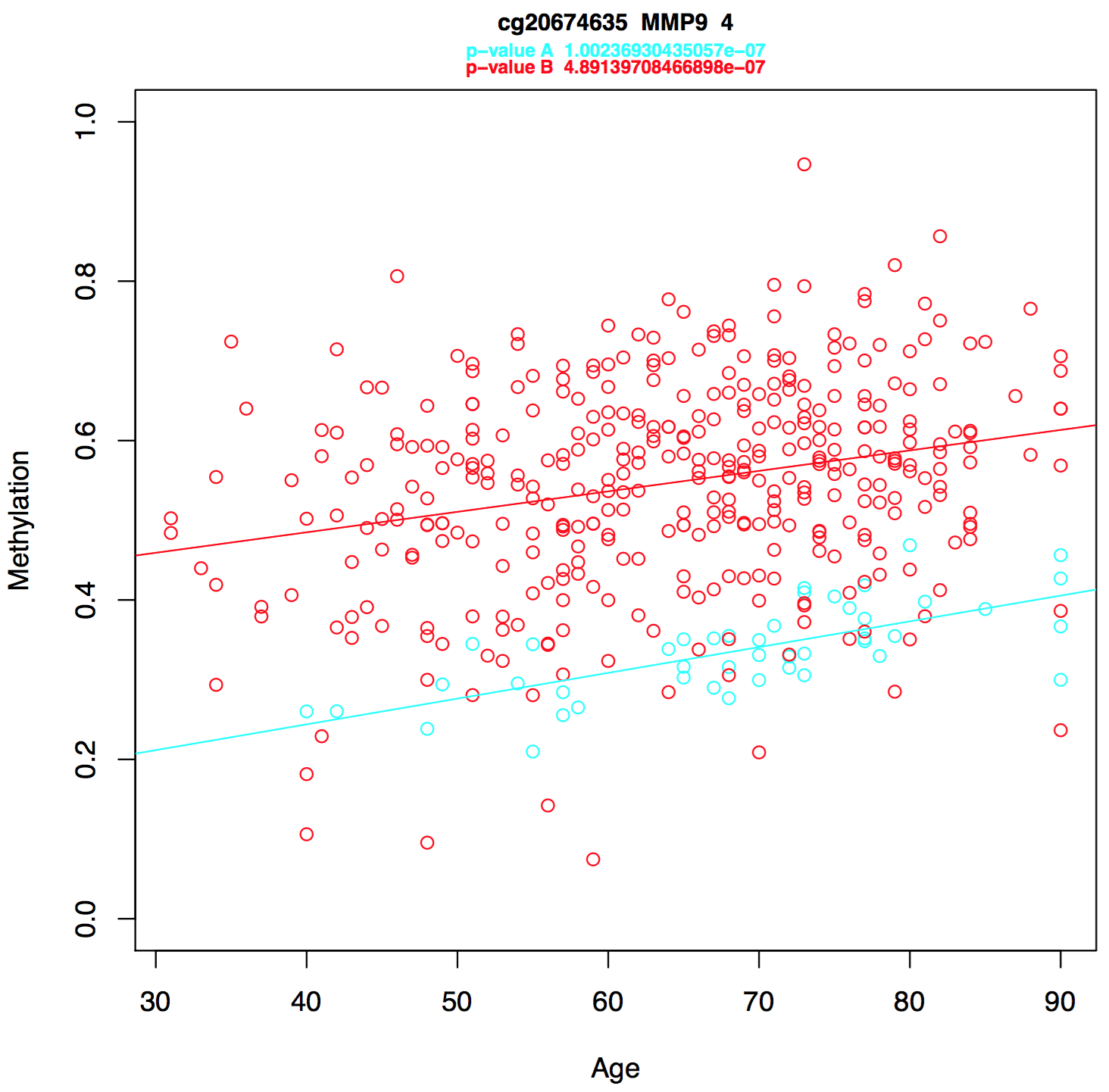

Supplement: Supplementary file 9 [file oncotarget-08-12820-s009.docx]
