# Supplementary material for: Aberrant methylation patterns in colorectal cancer: a meta-analysis
Source: Oncotarget. 2017 Jan 10;8(8):12820–30. doi: 10.18632/oncotarget.14590 (PMC5355058; doi:10.18632/oncotarget.14590)

**Supplementary File 9** - Boxplots showing methylation levels by BRAF and KRAS gene mutation status – abnormal and normal. We considered the DNA methylation value of the most significant CpG site within each BOP associated to the 10 N1xCRC DM hubs (Table 3) and plotted each methylation value against the age of the subjects. To each gene there are 12 plots showing the methylation levels of each CpG mapped in a DM N1xCRC hub.

:

**PAGES 2 to 13**

BRAF plots

**PAGES 14 to 25**

KRAS plots


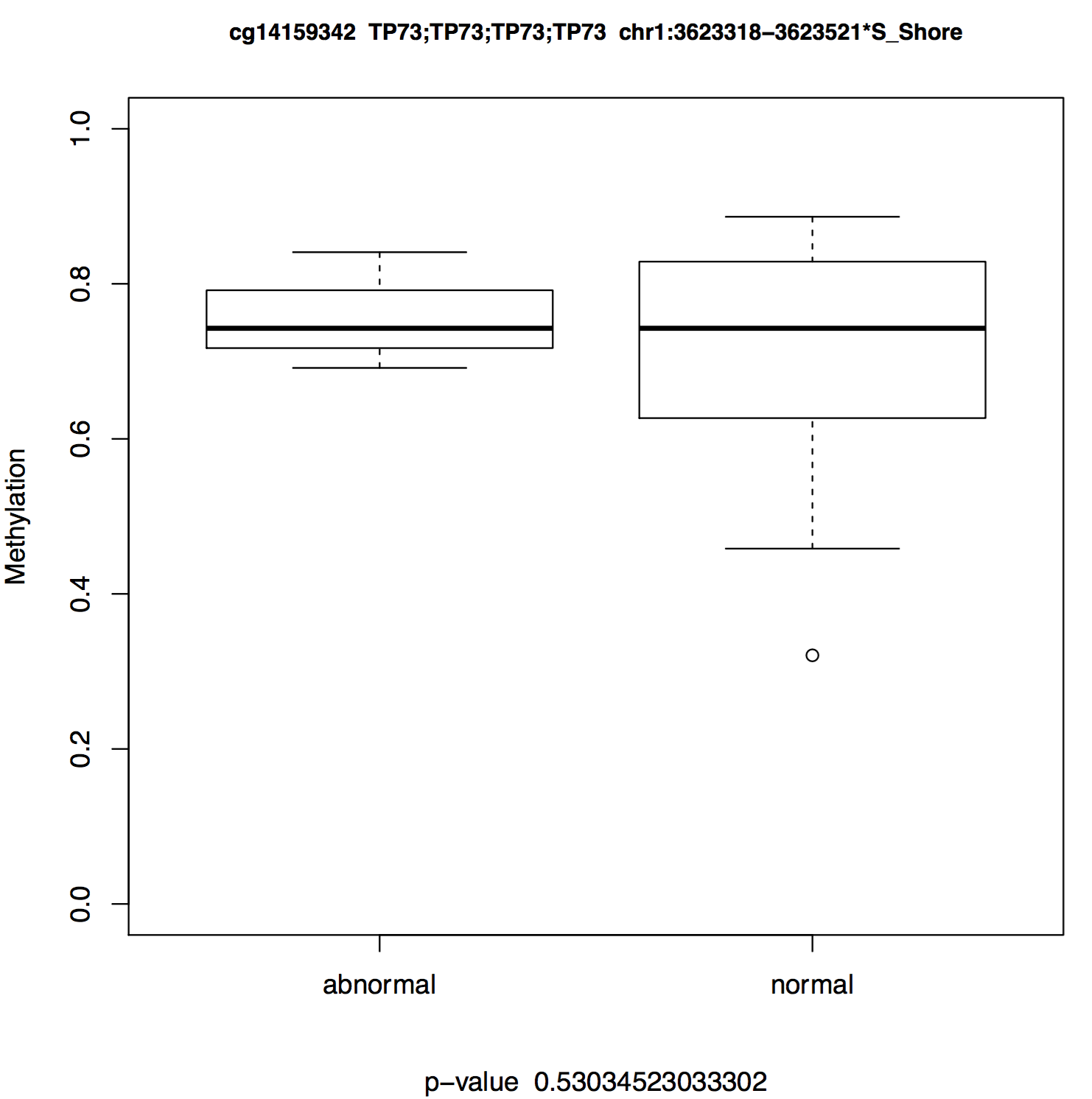

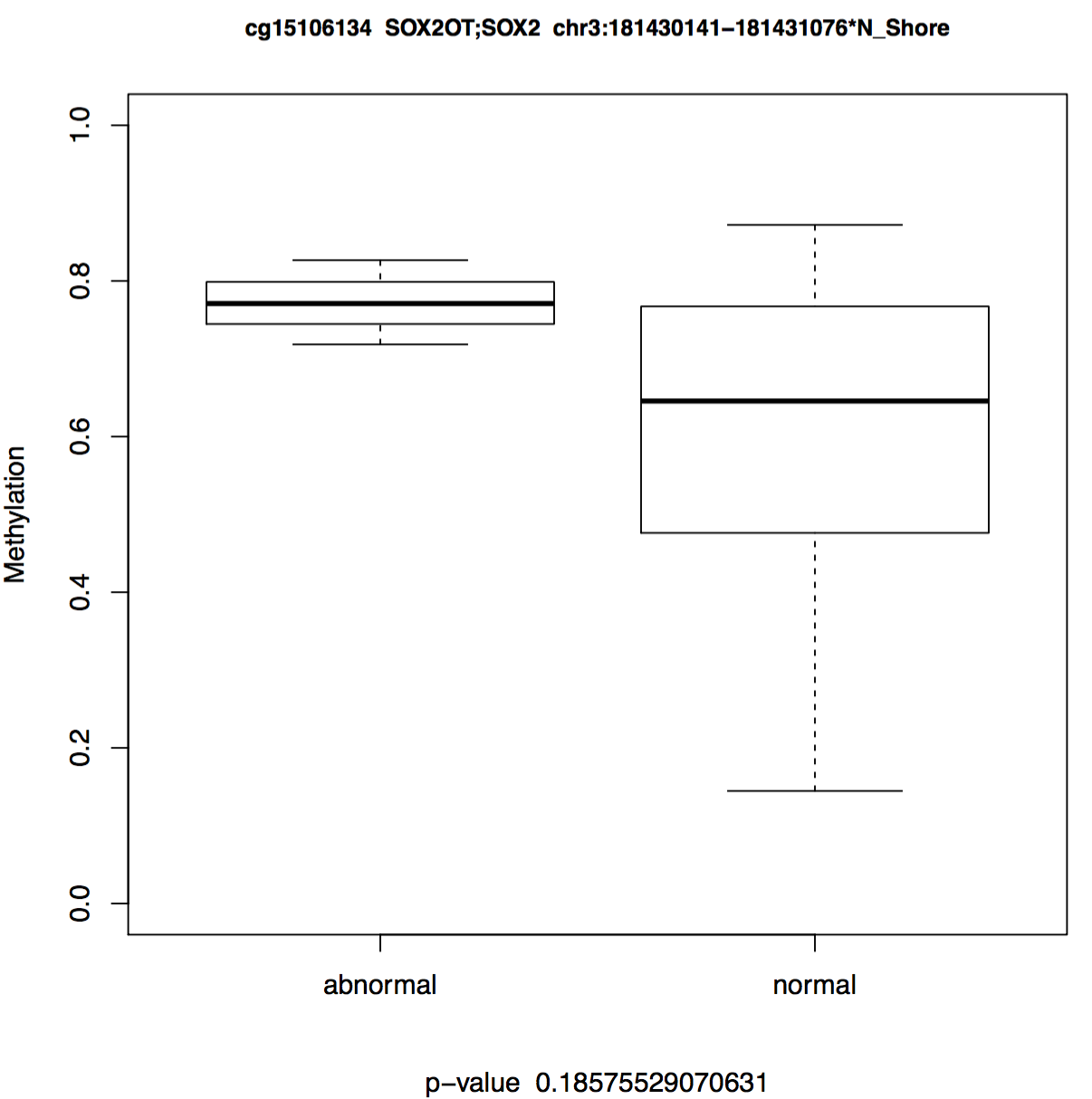

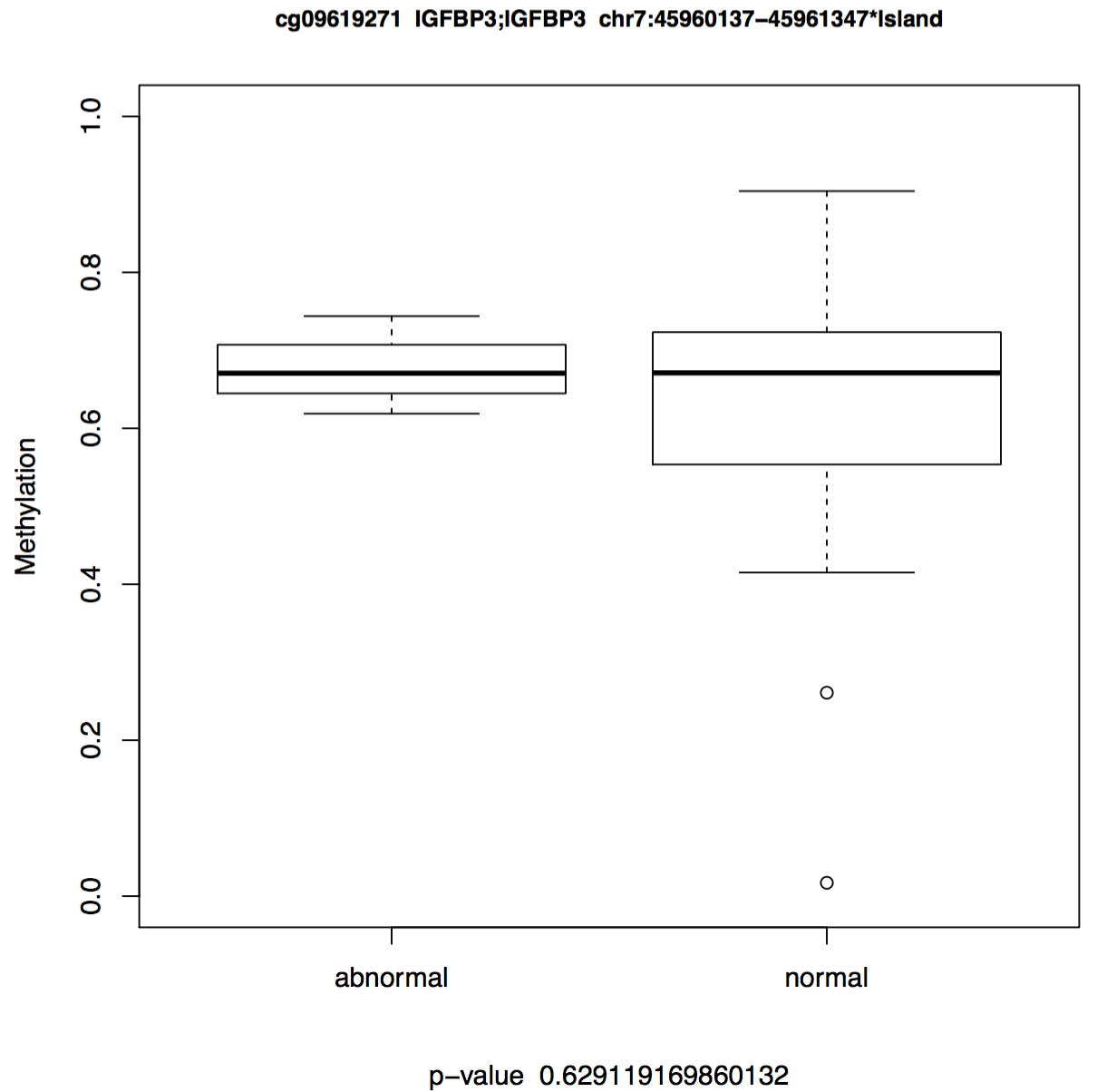

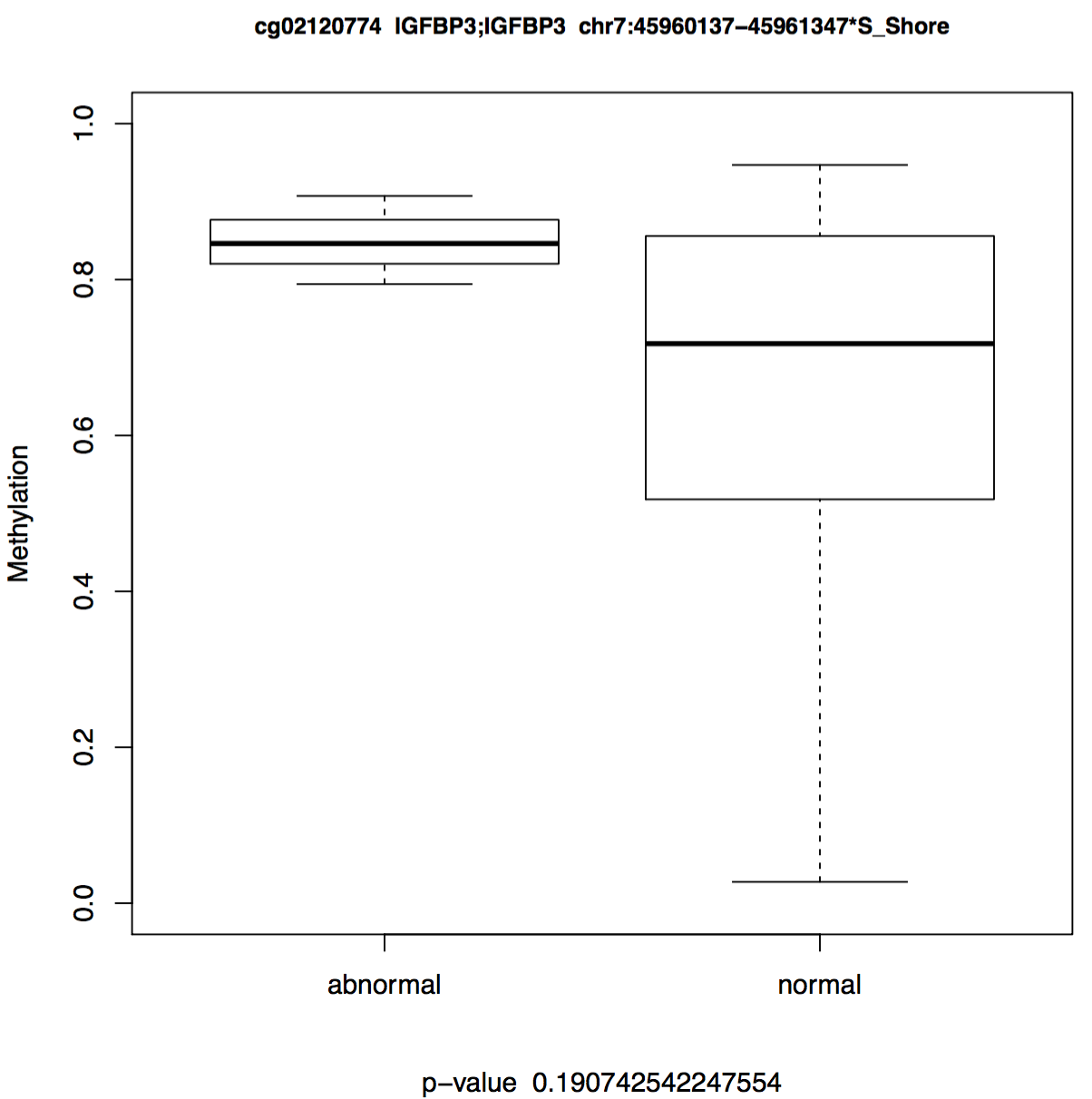

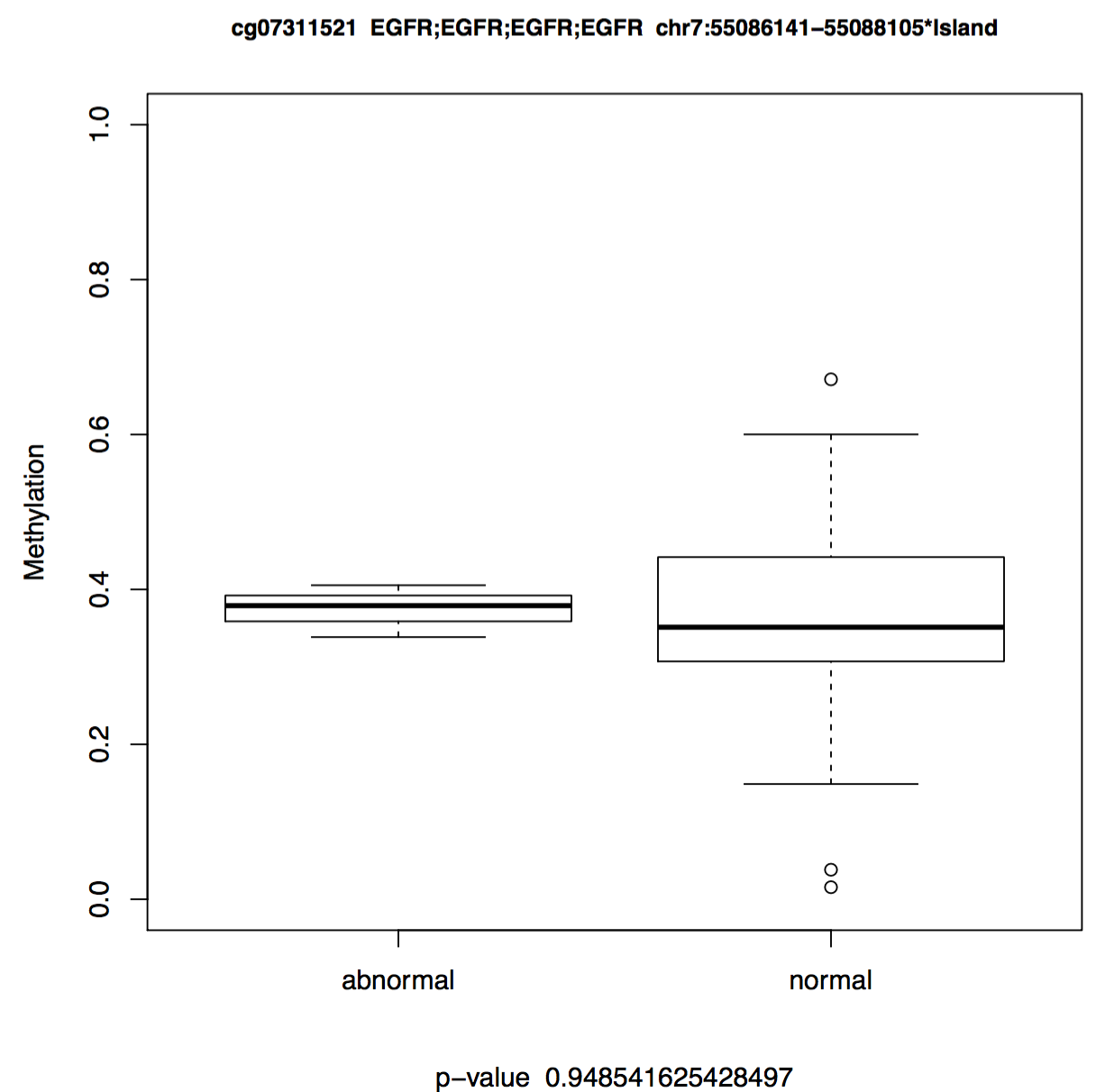

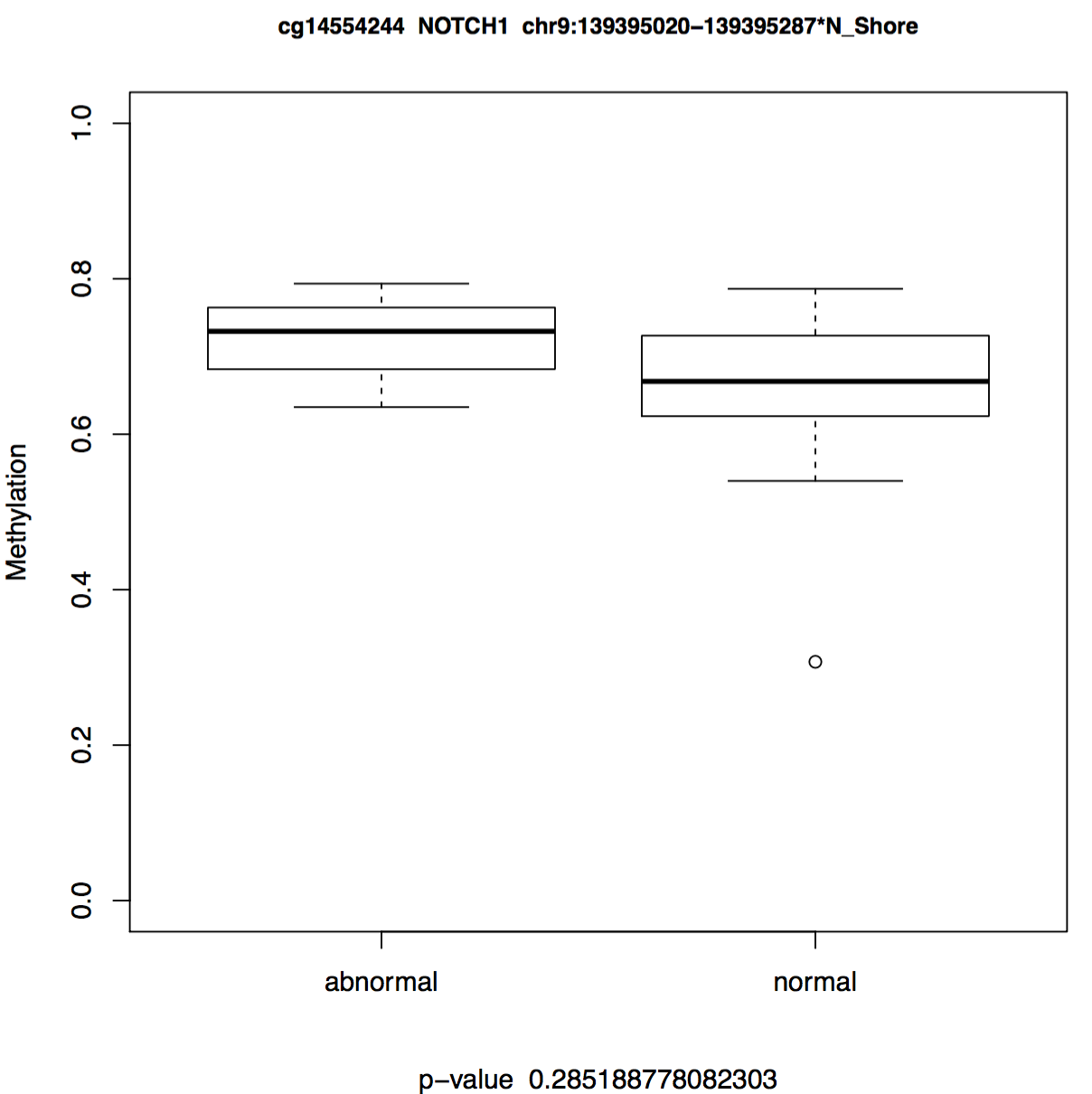

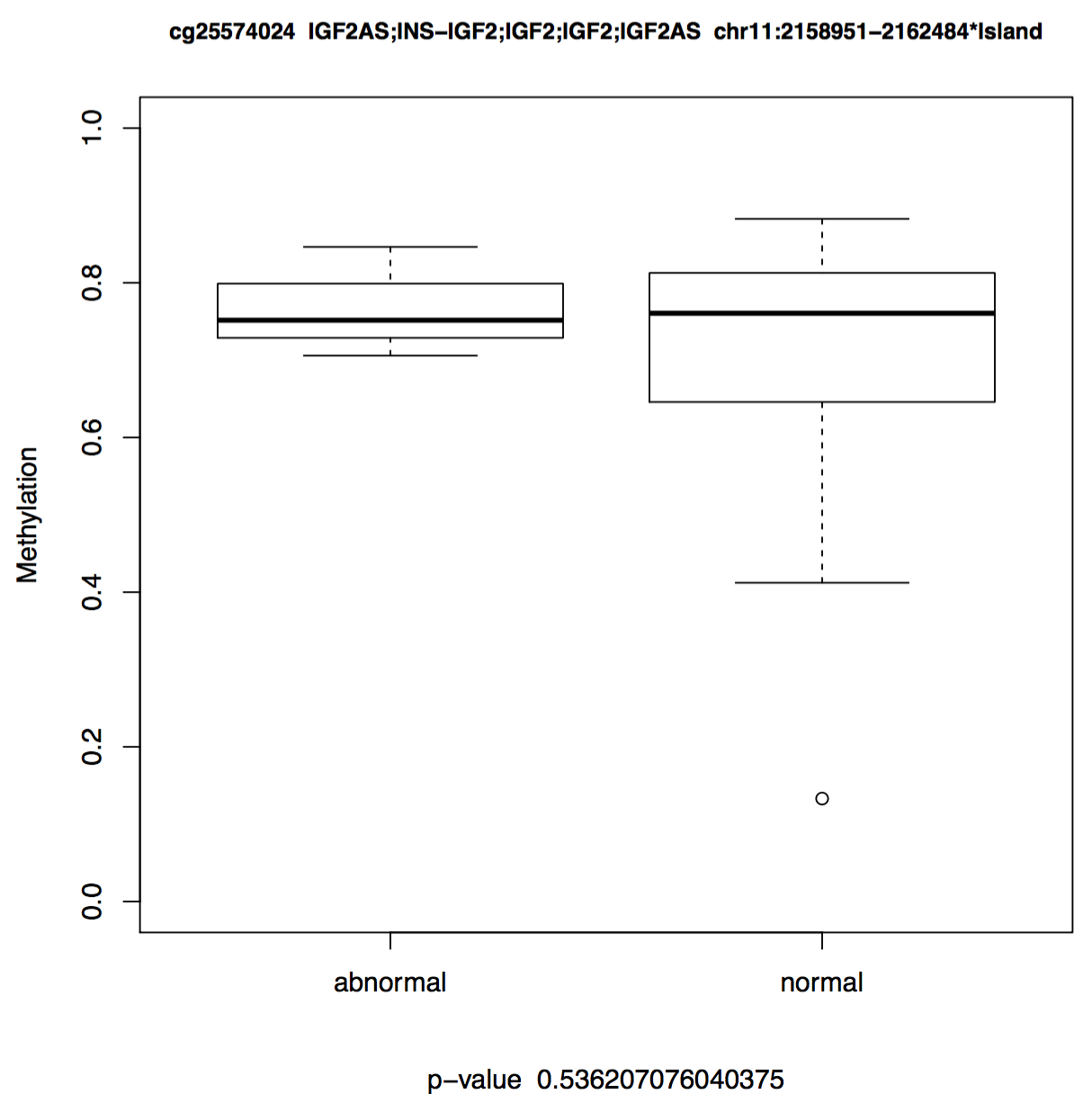

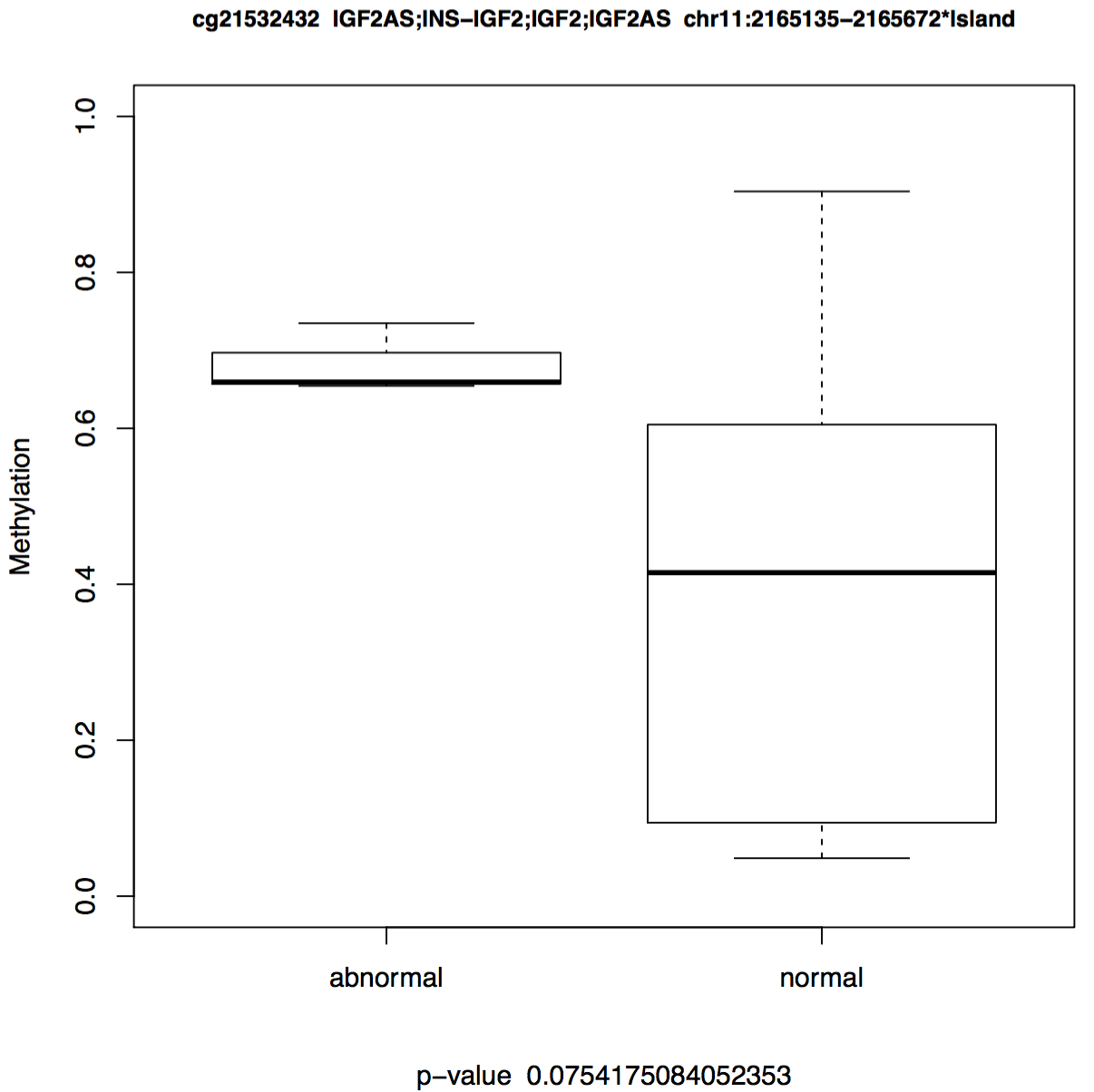

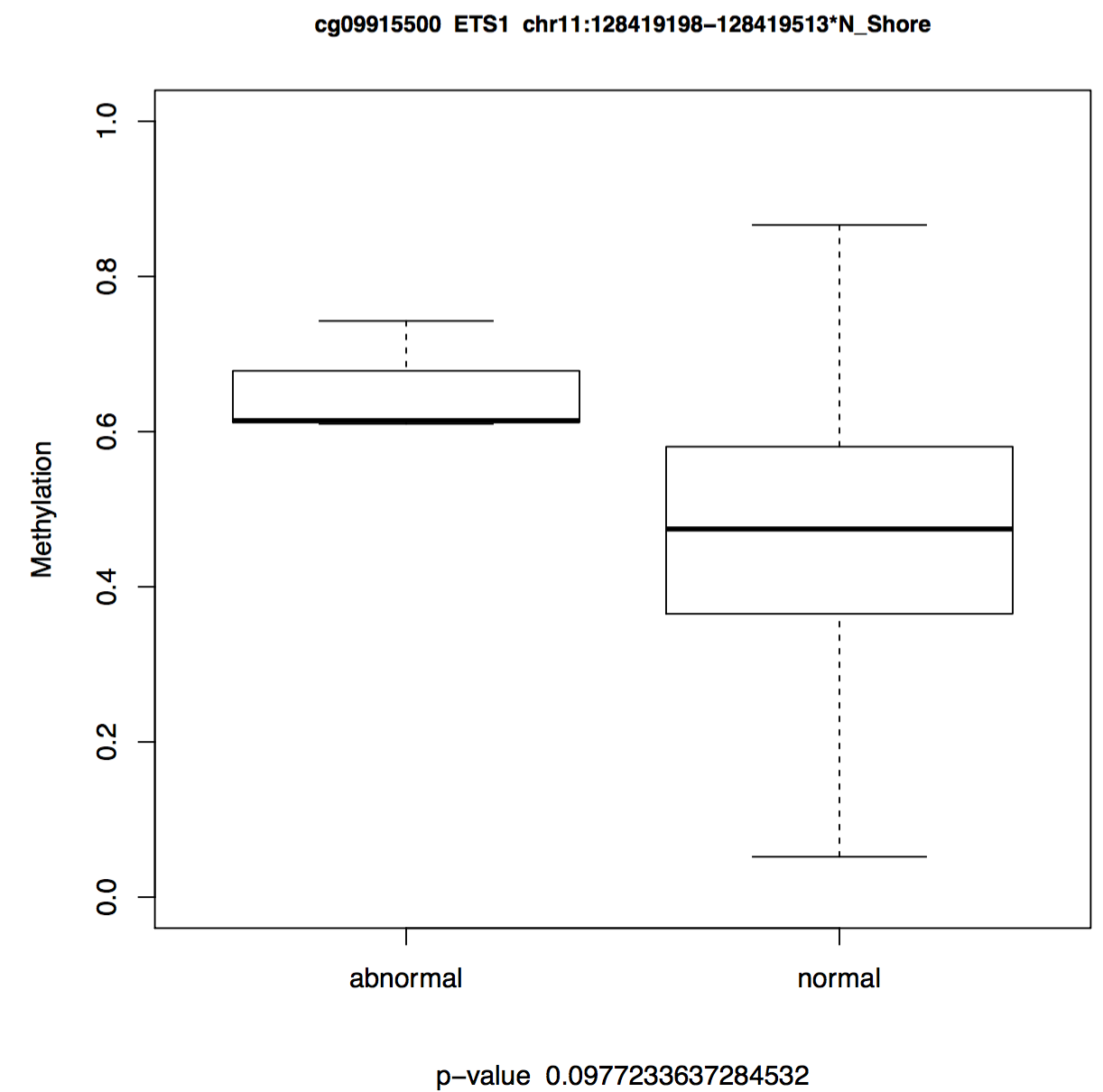

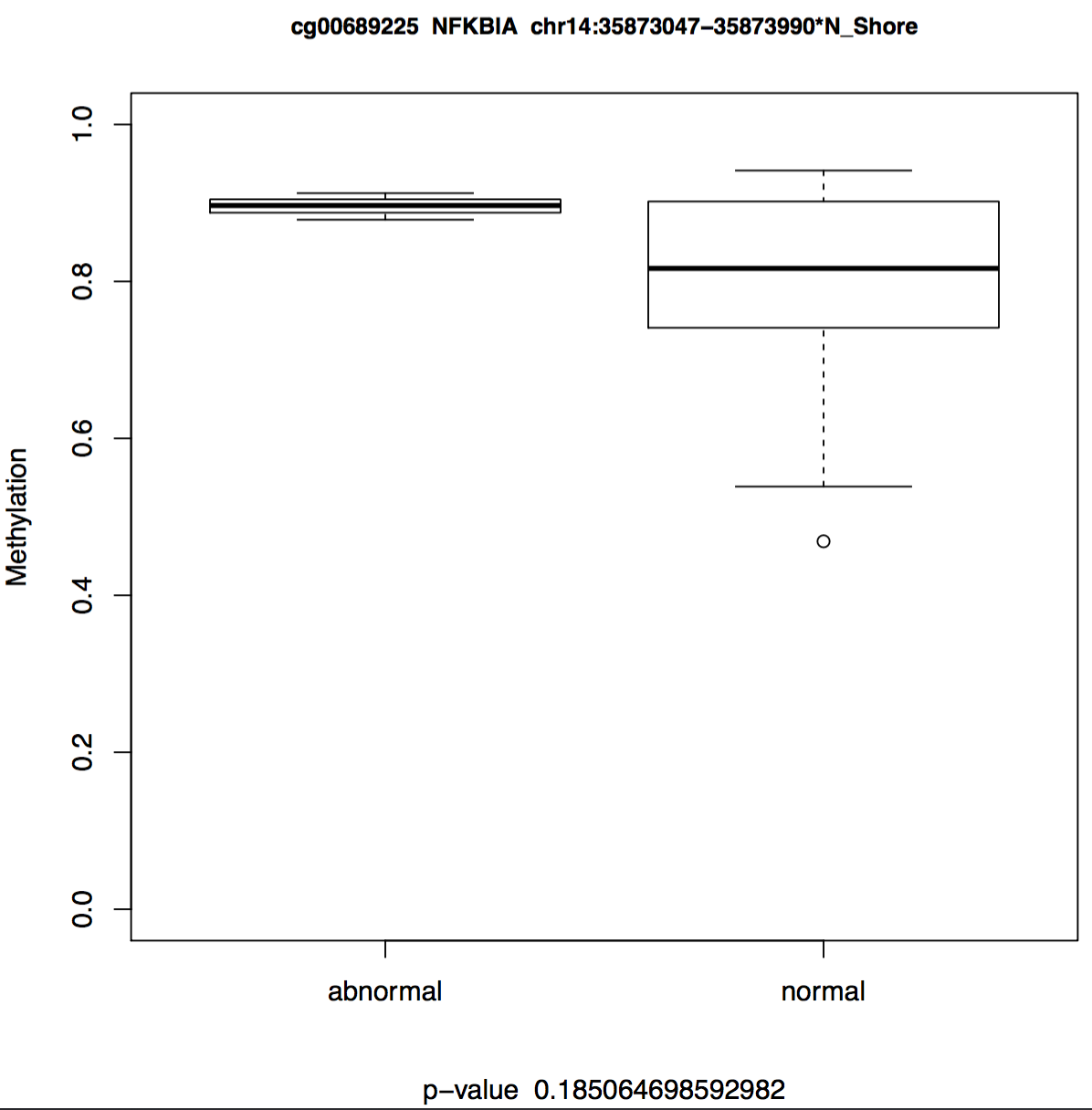

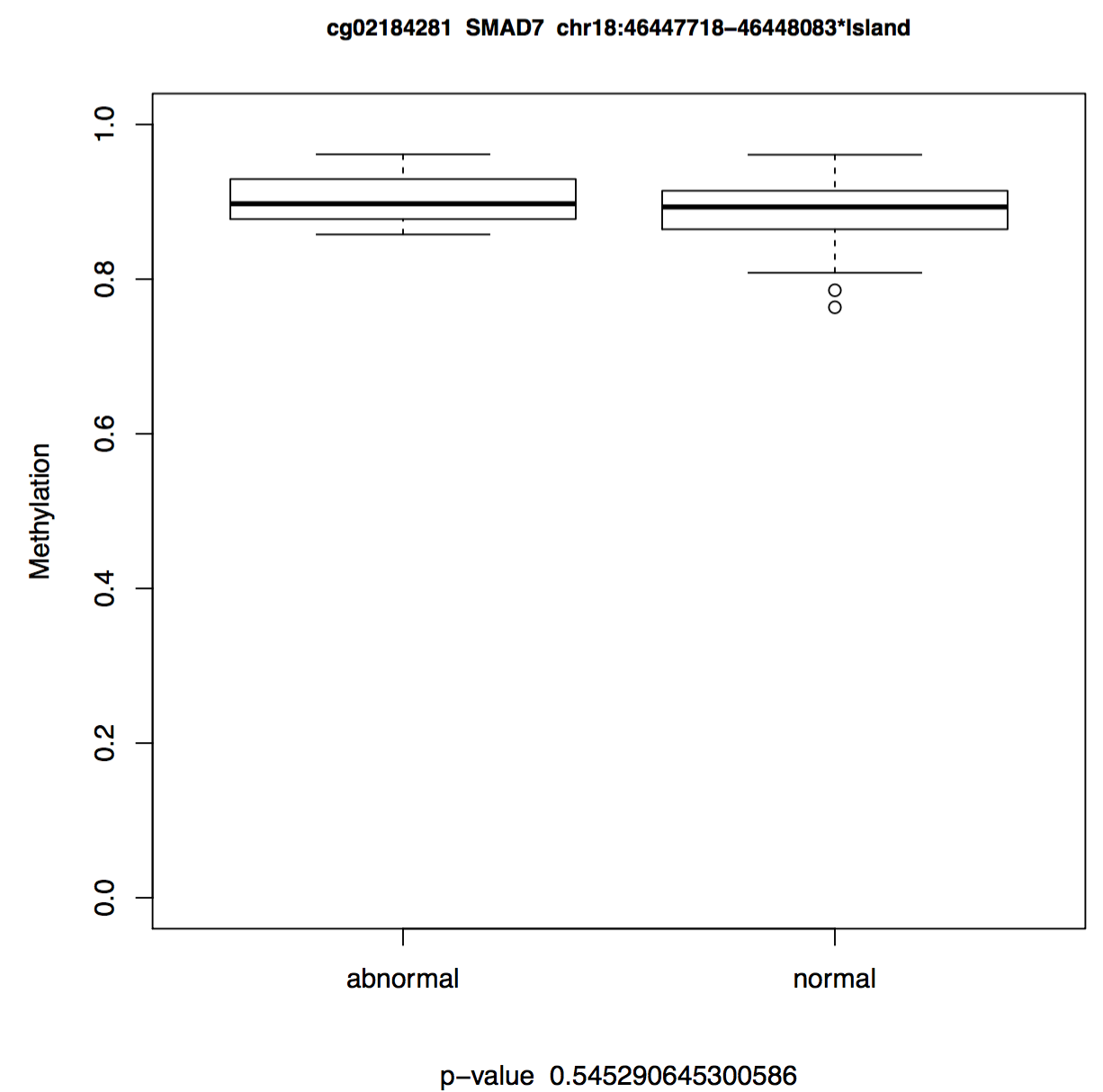

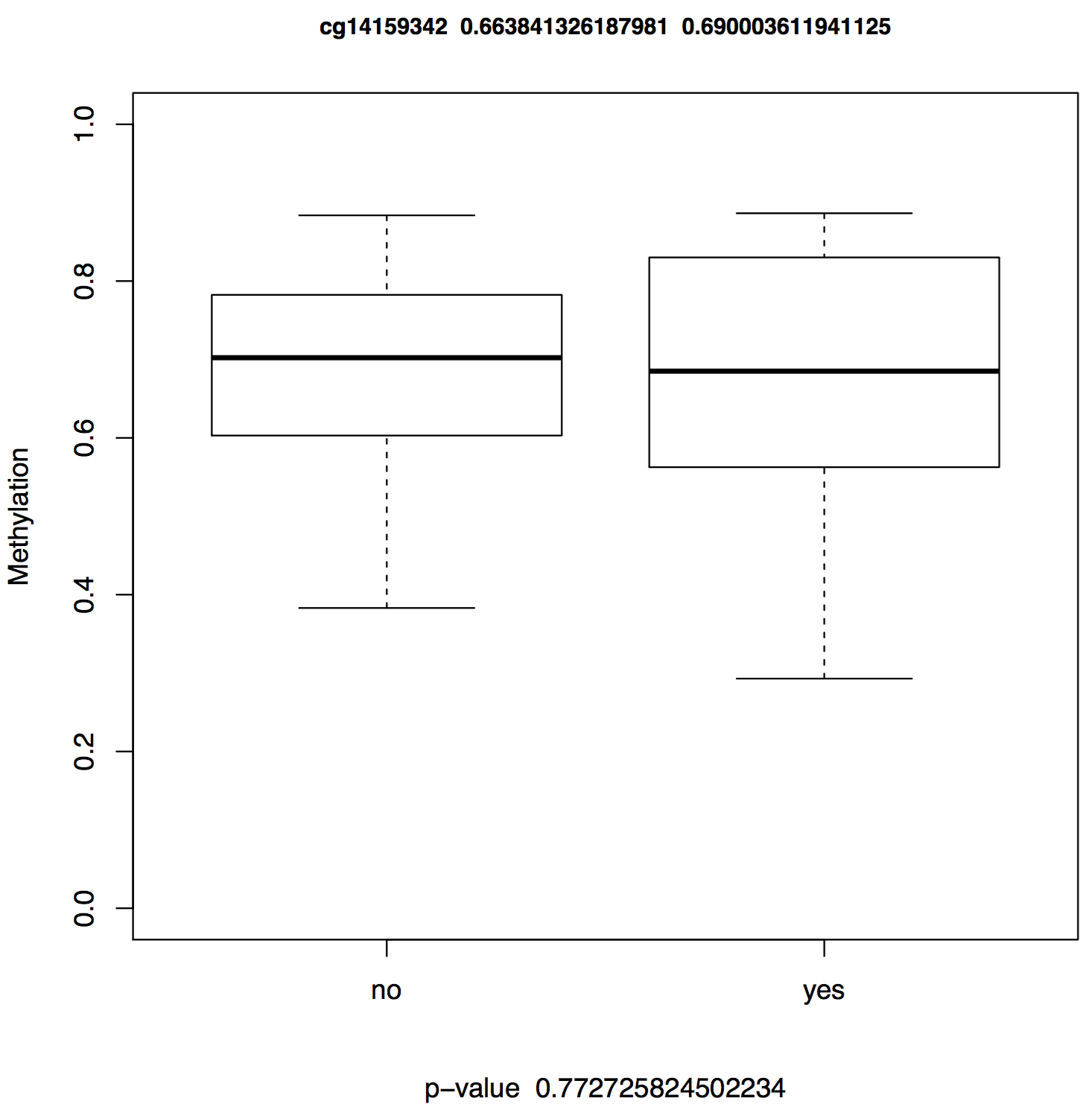


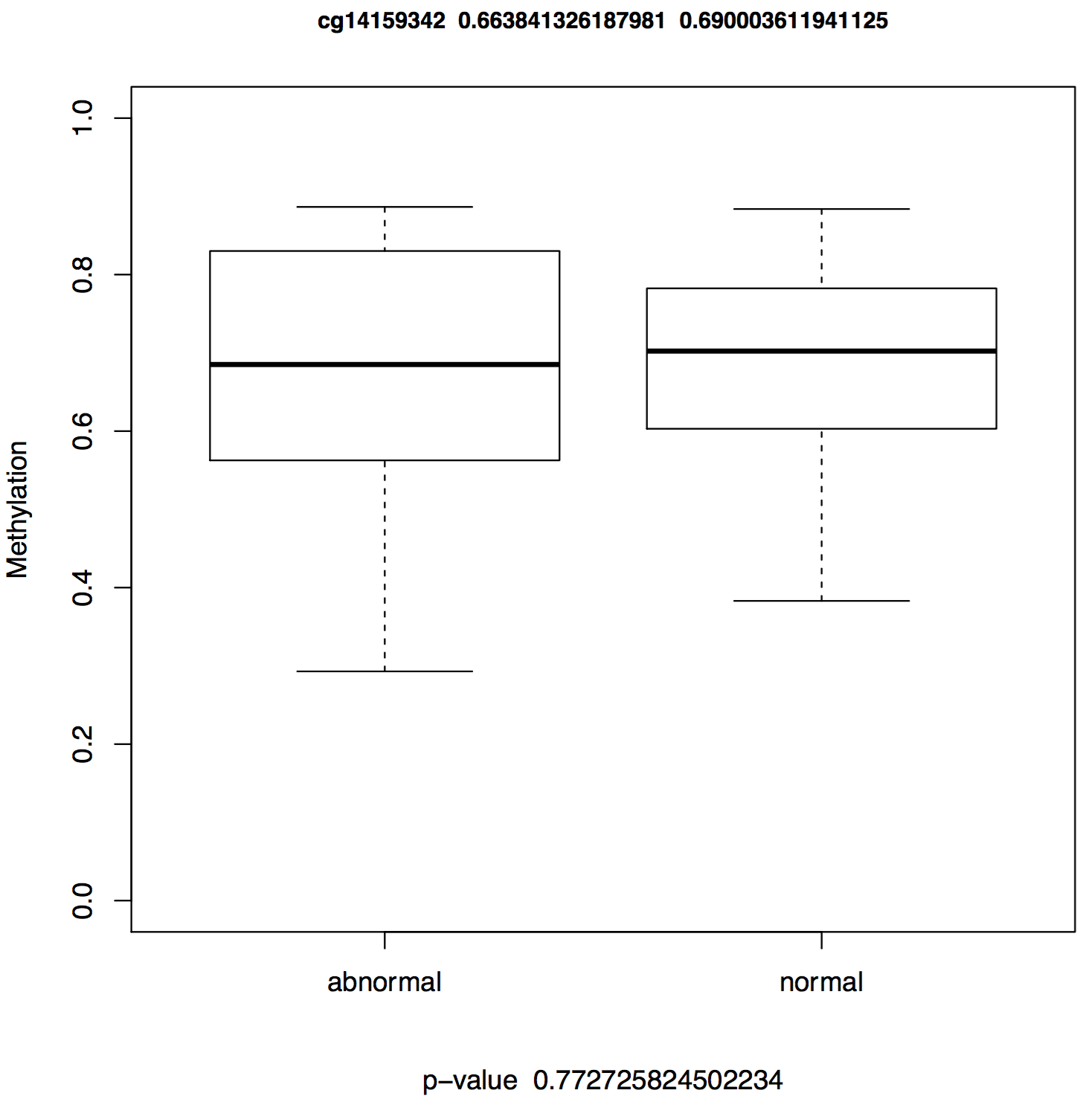

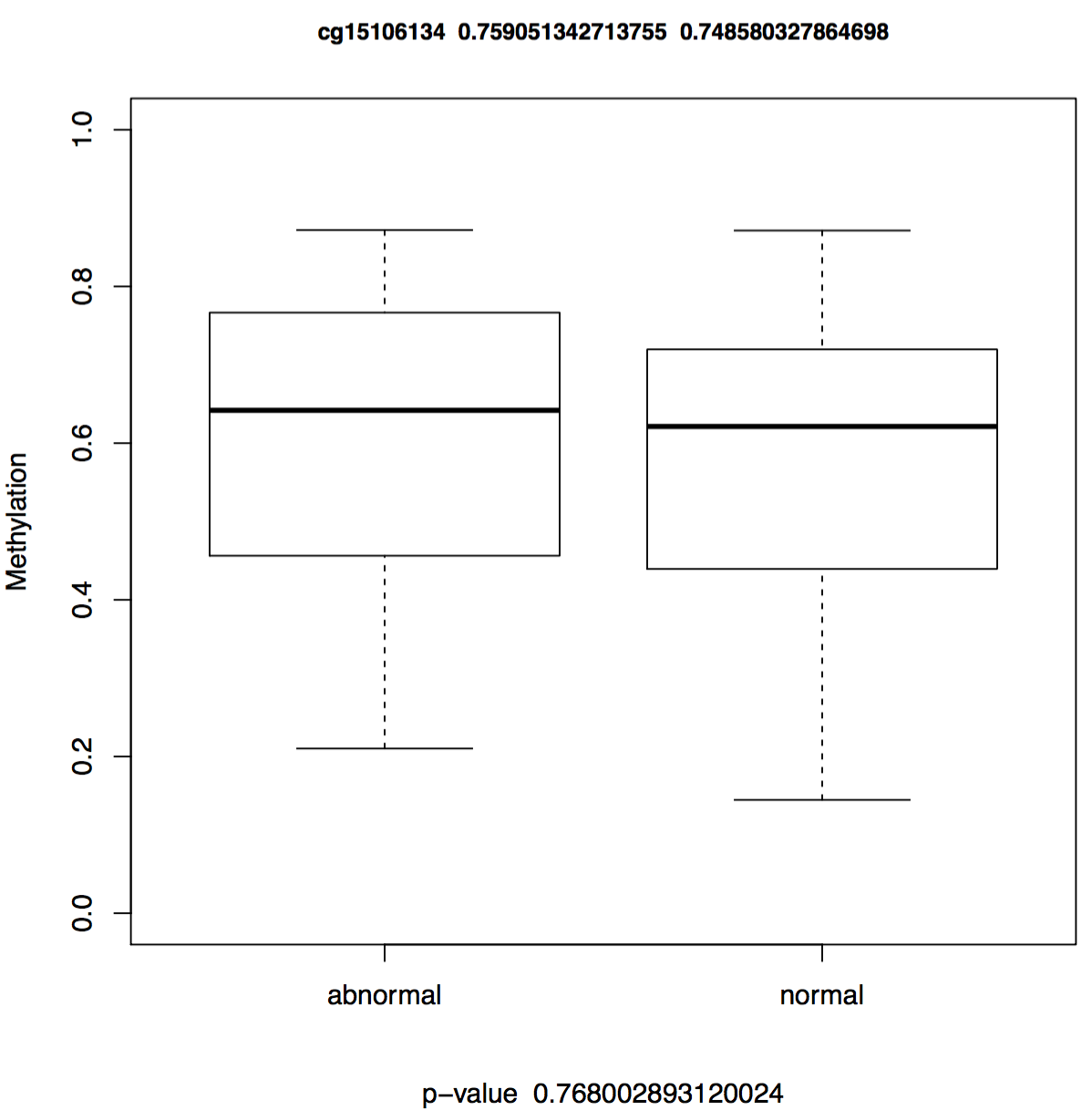

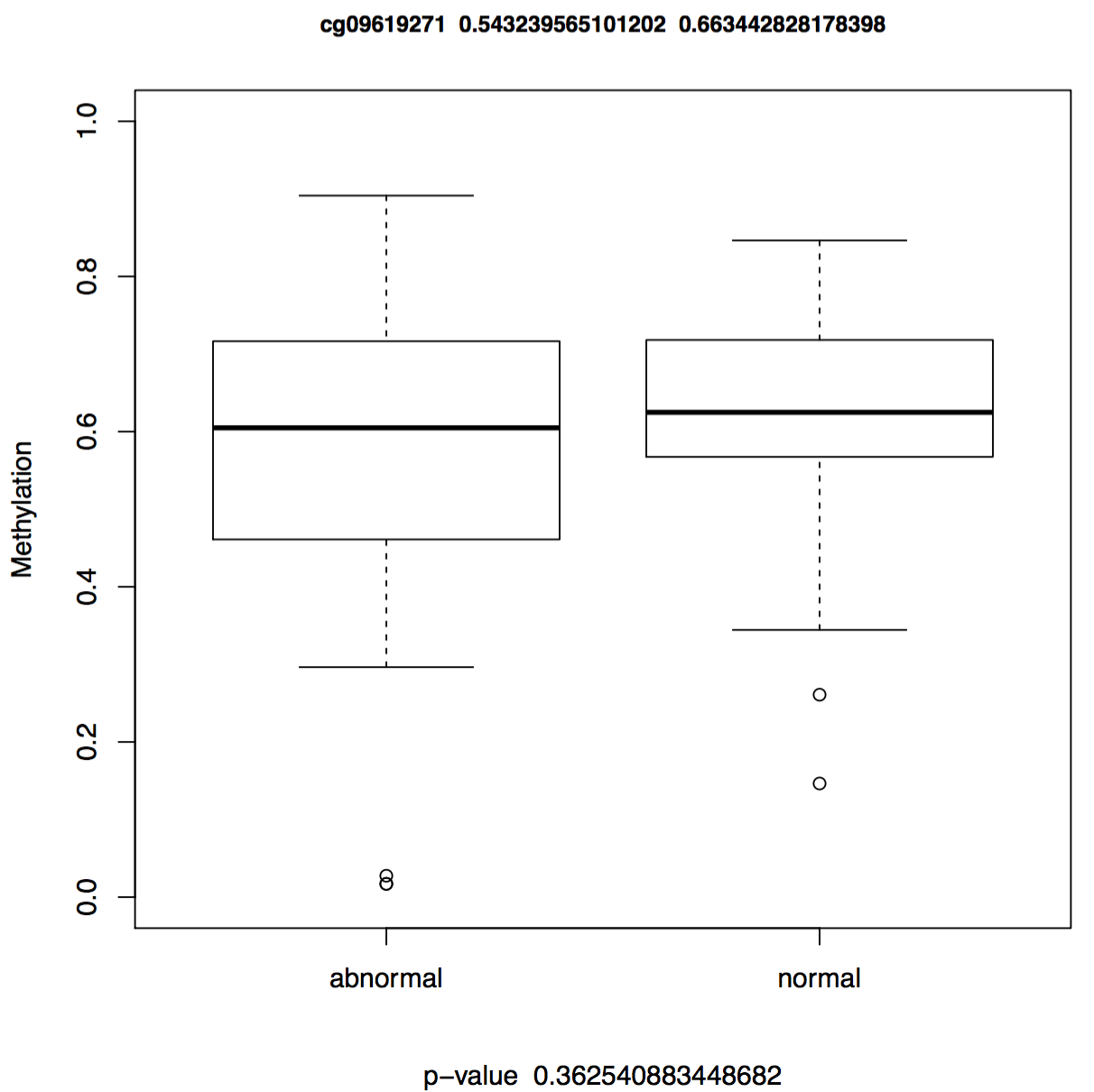

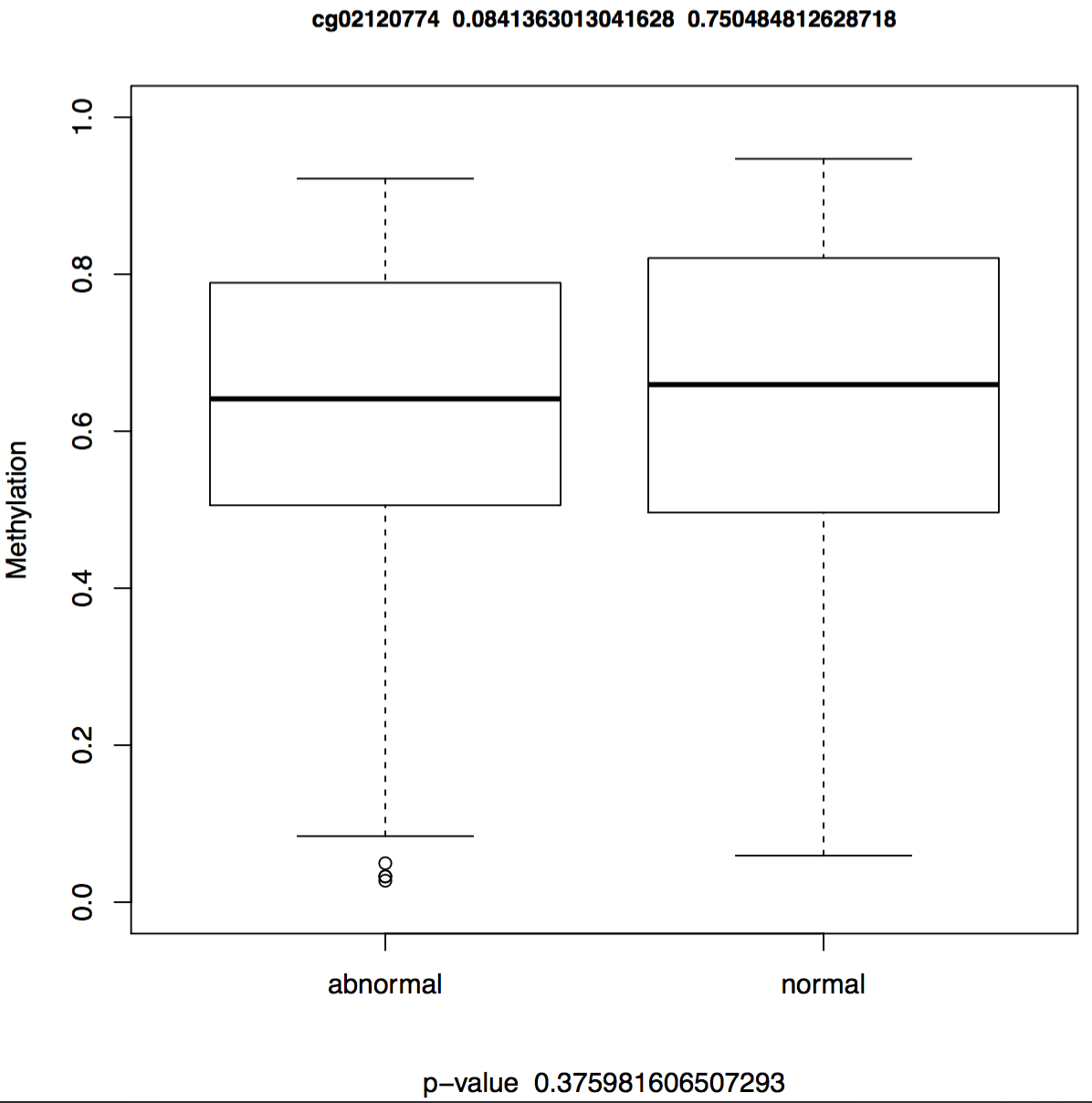

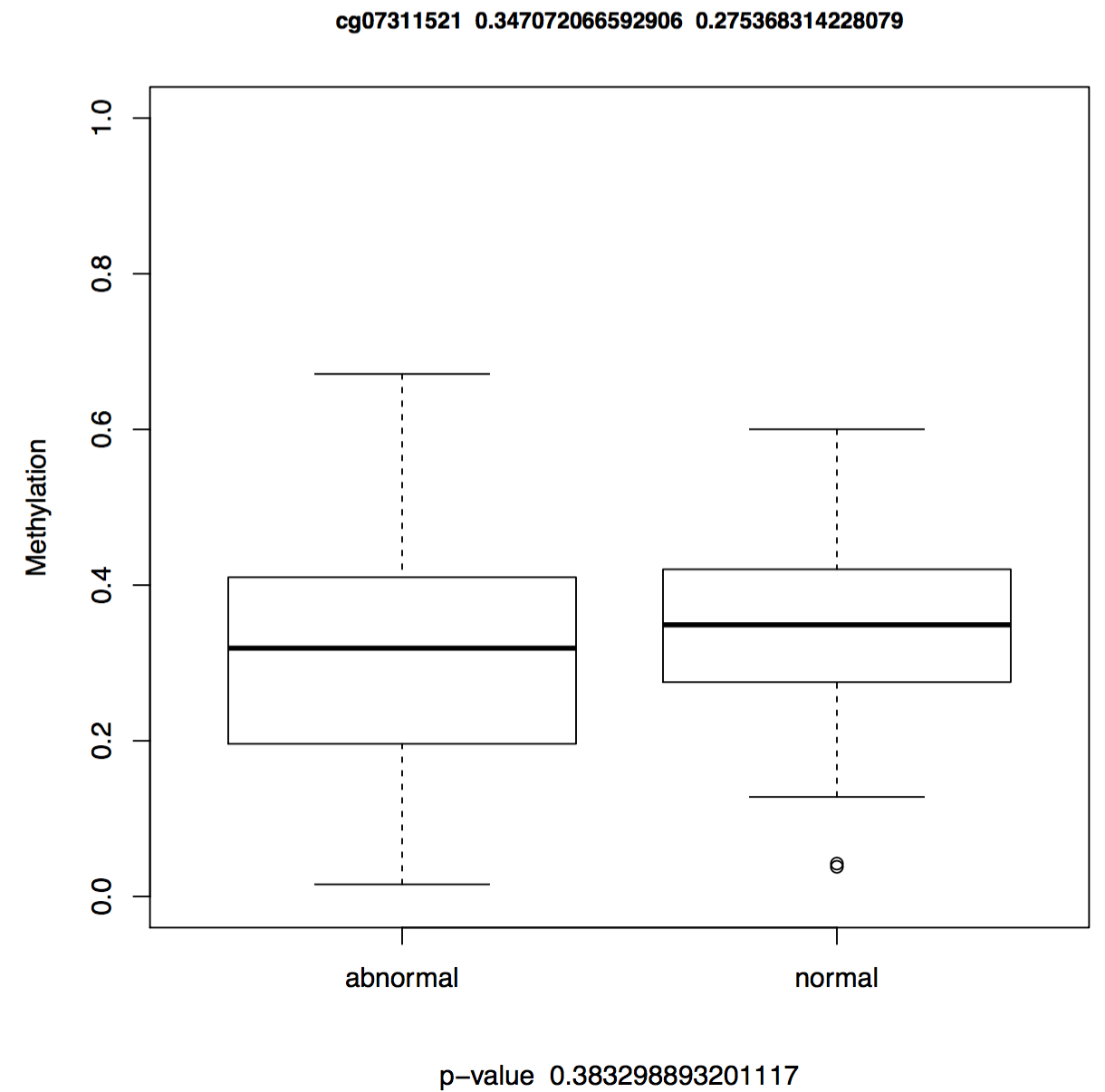

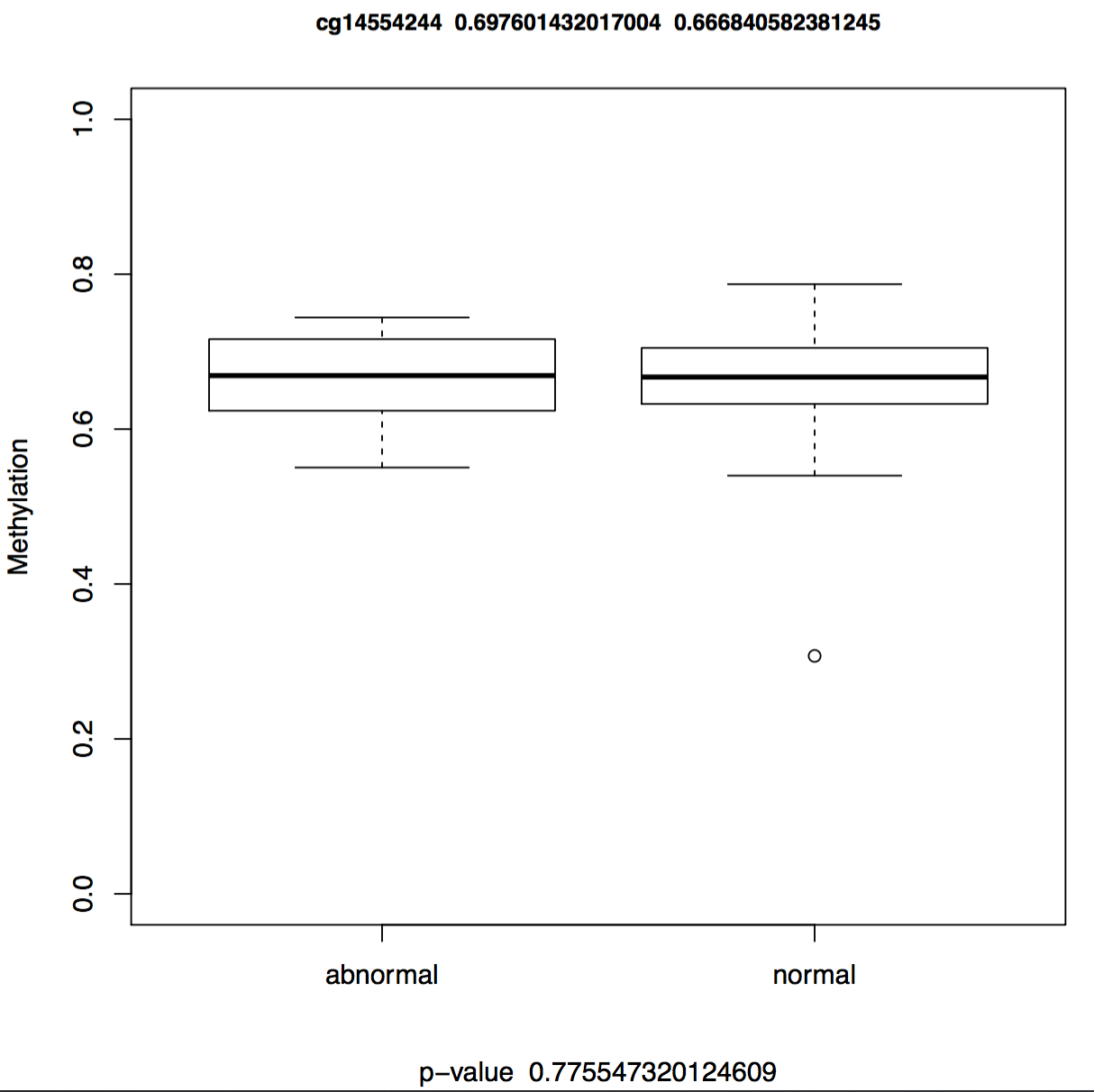

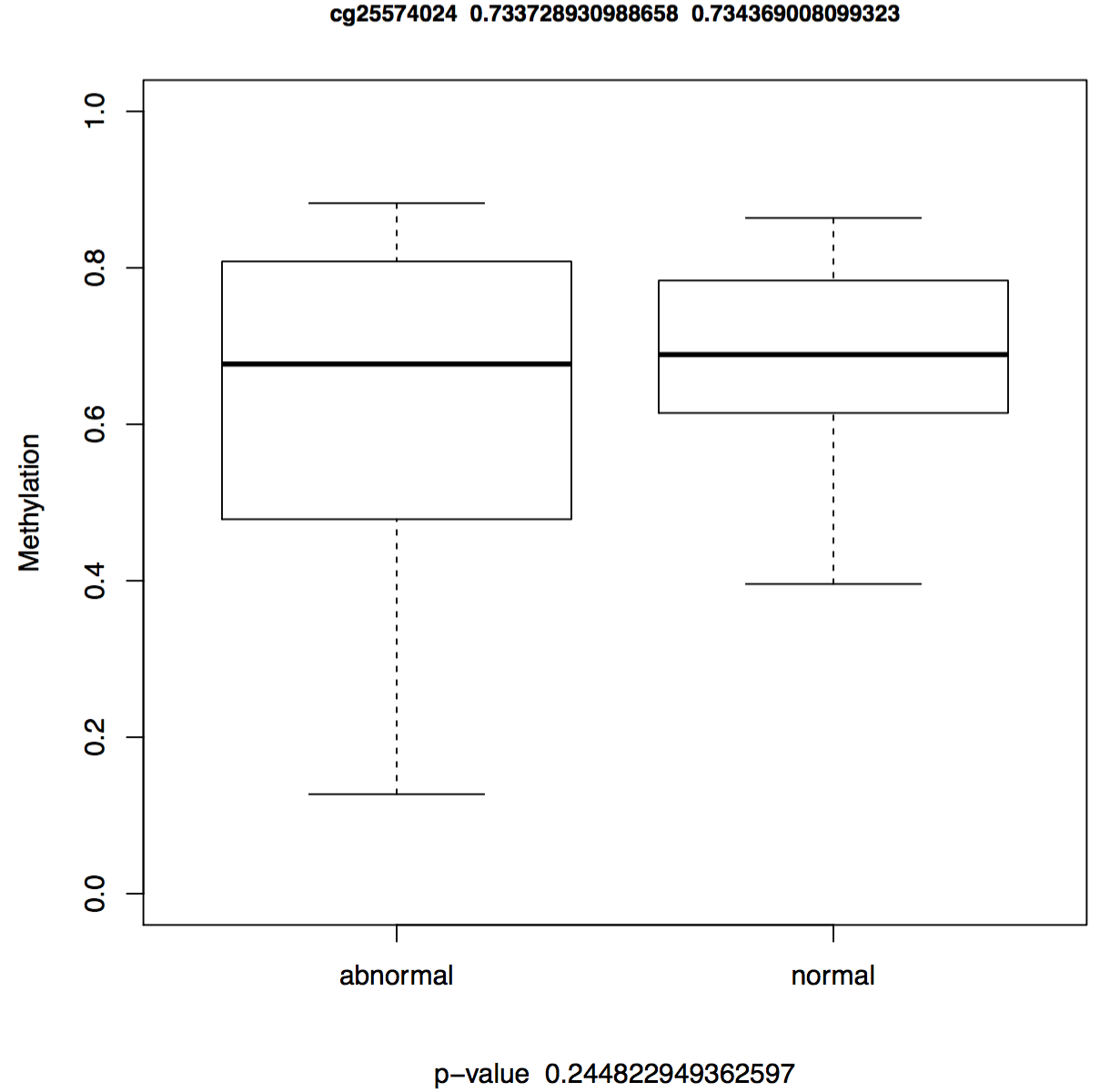

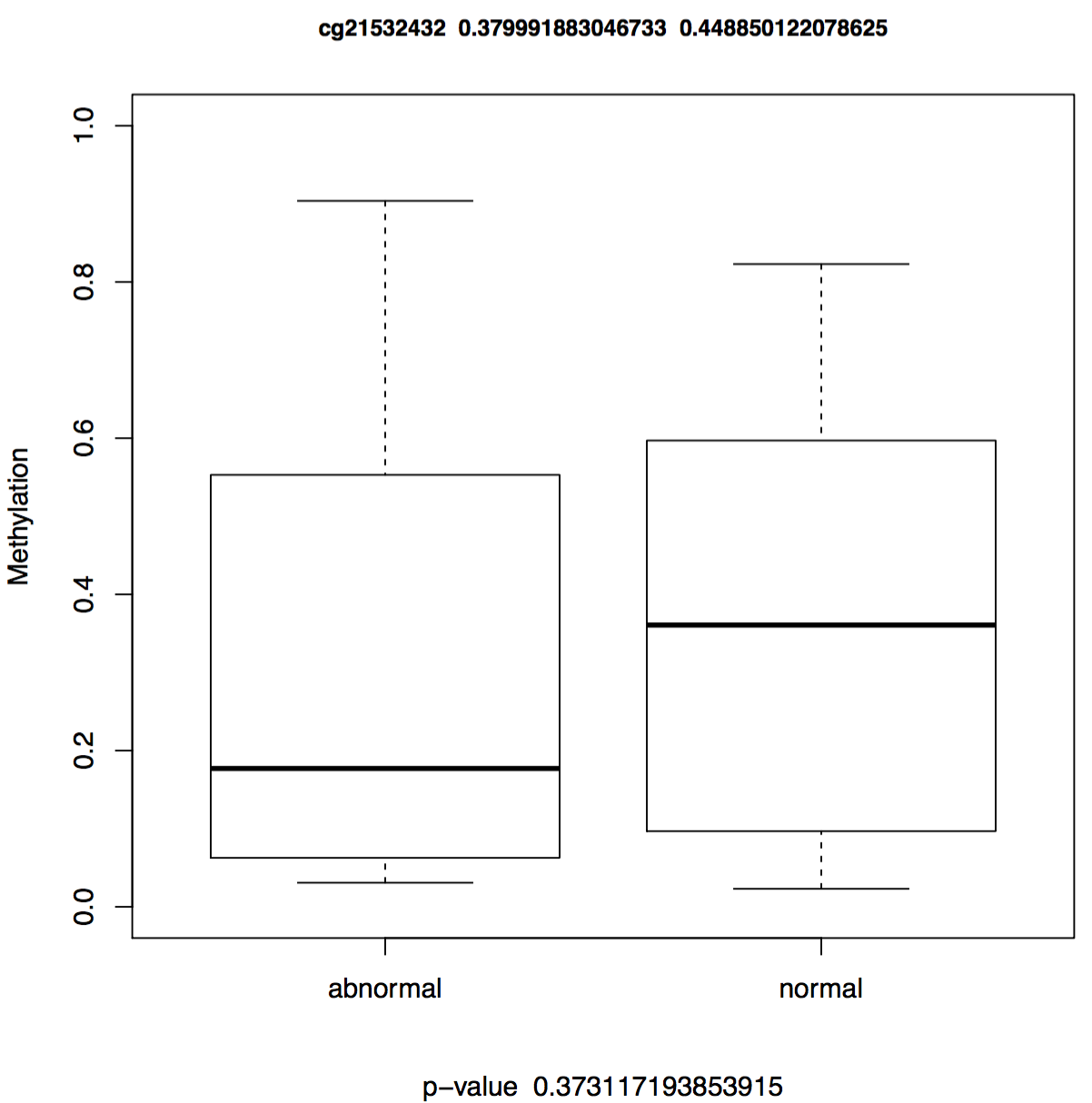

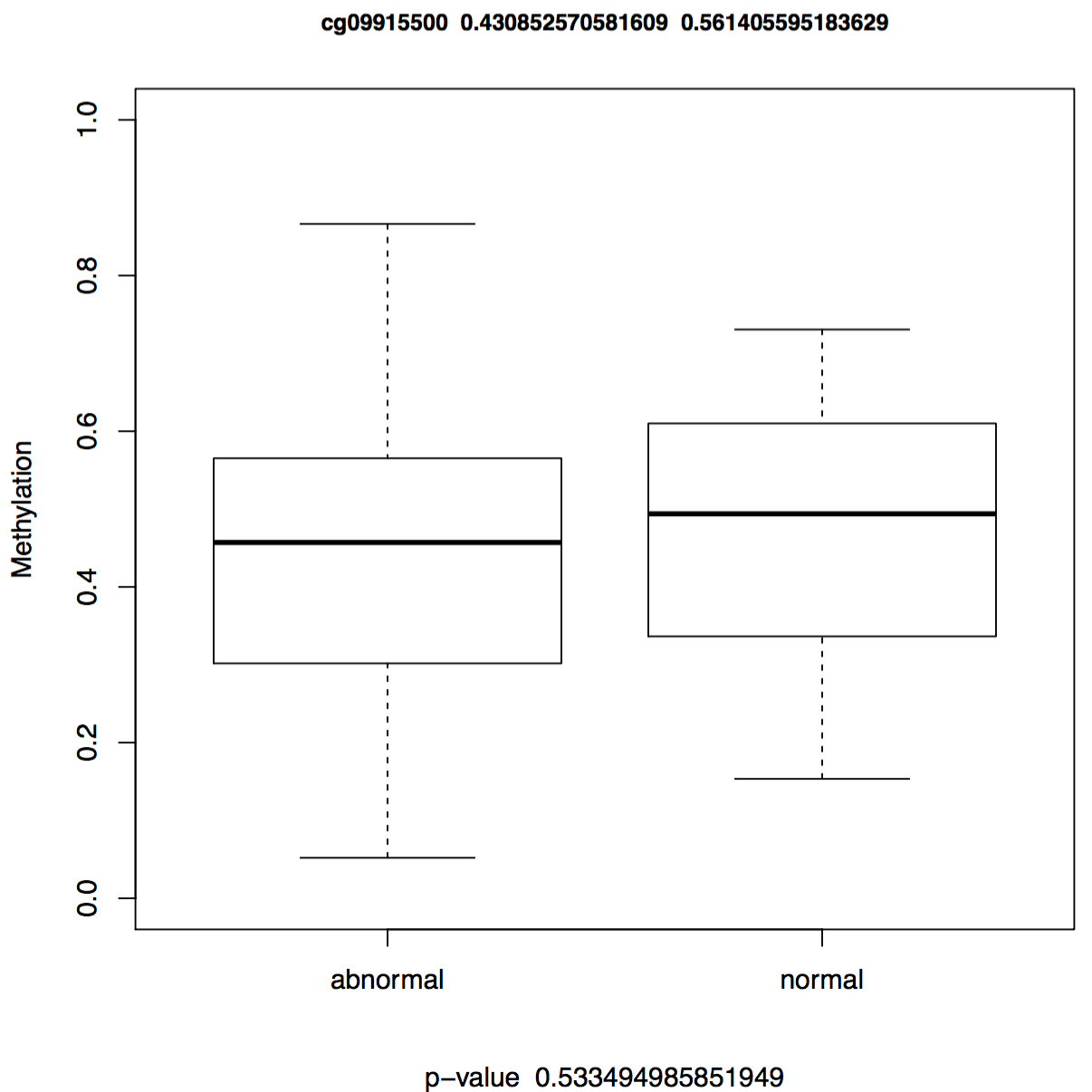

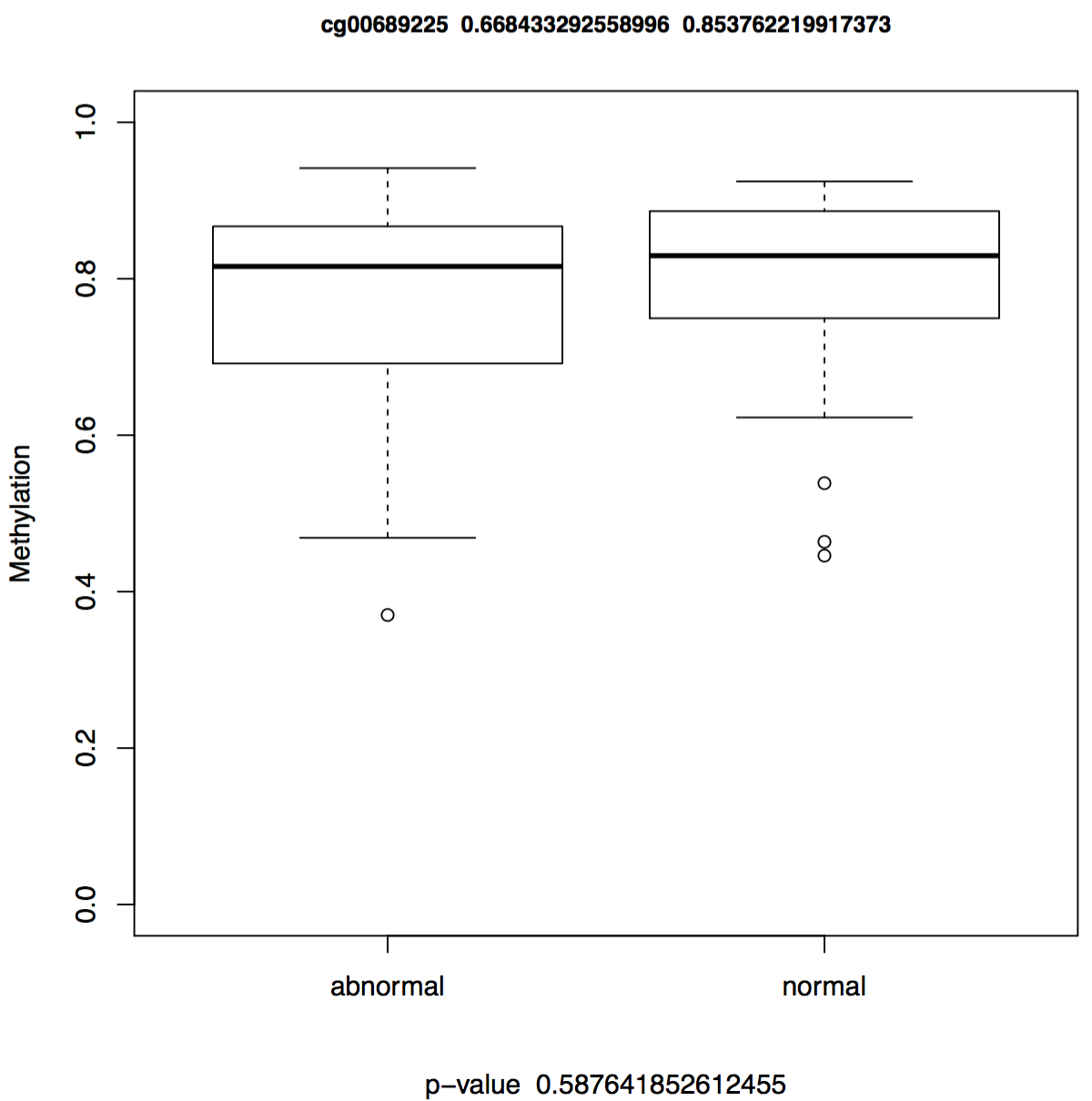

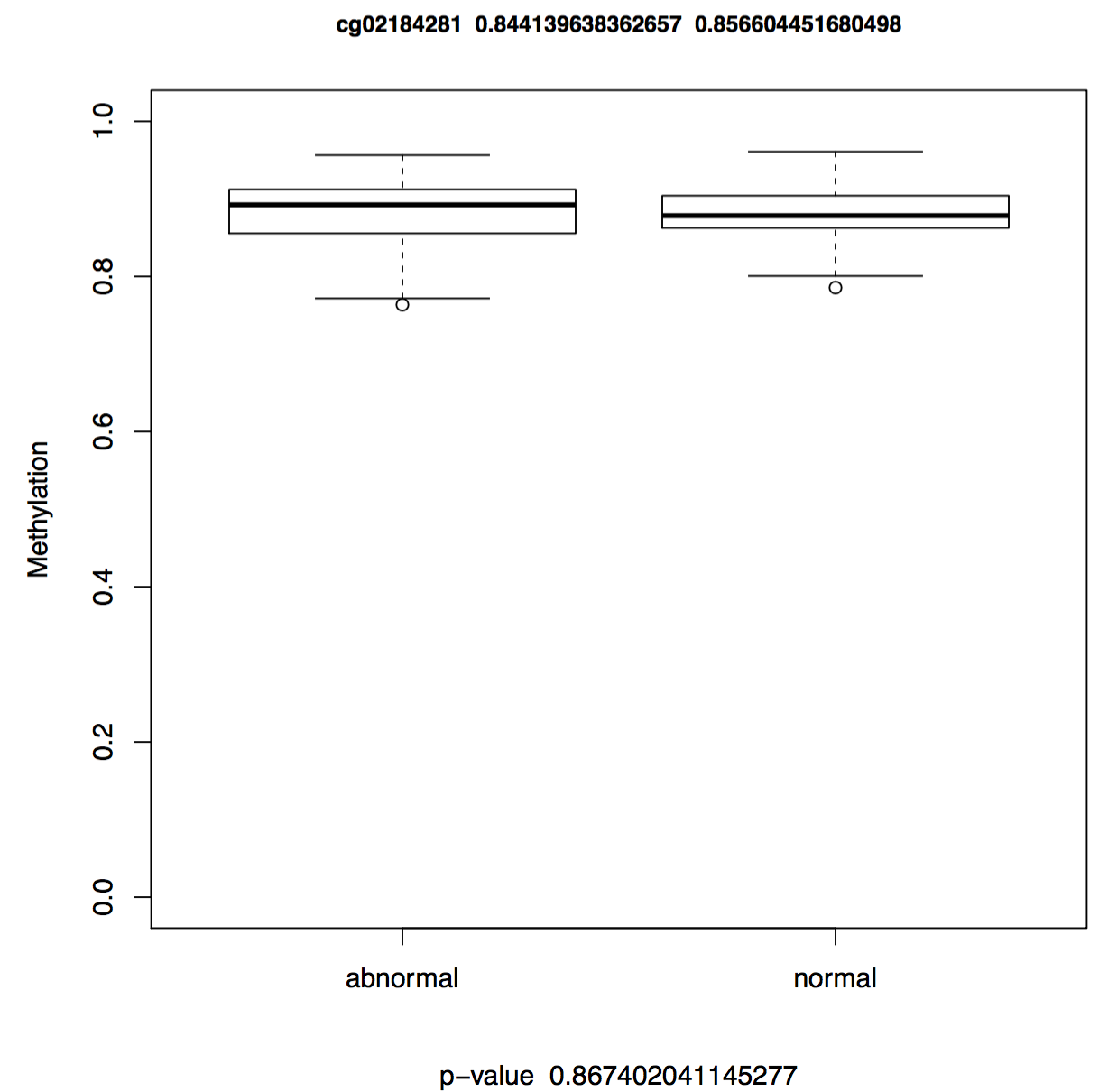

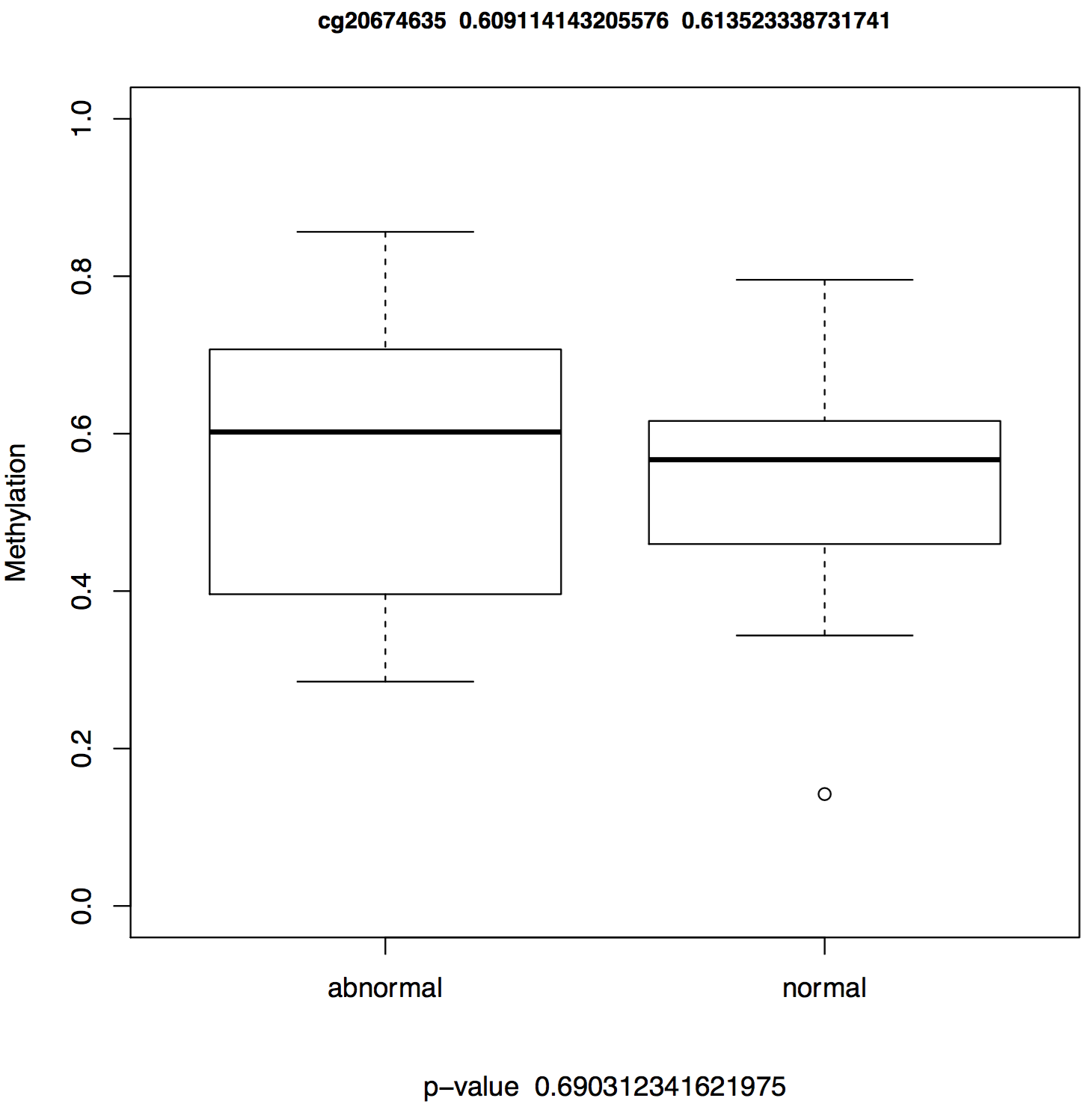

Supplement: Supplementary file 10 [file oncotarget-08-12820-s010.docx]
